# “On-Water” Synthesis of Quinazolinones and Dihydroquinazolinones Starting from *o*-Bromobenzonitrile

**DOI:** 10.3390/molecules23092325

**Published:** 2018-09-12

**Authors:** Zibin Liu, Li-Yan Zeng, Chao Li, Fubiao Yang, Fensheng Qiu, Shuwen Liu, Baomin Xi

**Affiliations:** Guangdong Provincial Key Laboratory of New Drug Screening, School of Pharmaceutical Sciences, Southern Medical University, Guangzhou 510515, China; liuzibin1007@126.com (Z.L.); Zengly2011@hotmail.com (L.-Y.Z.); LC151820cg@126.com (C.L.); 15625123630@163.com (F.Y.); qiufensheng1996@163.com (F.Q.)

**Keywords:** quinazolinones, dihydroquinazolinones, “on-water” reaction, selective synthesis

## Abstract

A versatile and practical “on-water” protocol was newly developed to synthesize quinazolinones using *o*-bromobenzonitrile as a novel starting material. Studies have found that air as well as water plays an important role in synthesis of quinazolinones. Further investigation indicated that dihydroquinazolinones can be prepared with this protocol under the protection of N_2_. The protocol can be extended to other substrates and various quinazolinones and dihydroquinazolinones were obtained. *o*-Bromobenzamide, *o*-aminobenzonitrile, and *o*-aminobenzamide were also evaluated as starting materials, and the results further proved the versatility of this protocol, especially towards dihydroquinazolinones.

## 1. Introduction

**Figure 1:**
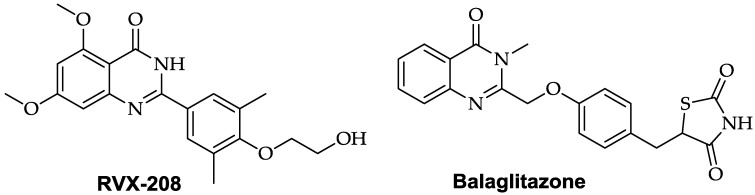
Structures of RVX-208 and balaglitazone.

Quinazolinones and dihydroquinazolinons are important classes of nitrogen-containing heterocycles with an array of biological activities such an antitumor [1,2], anti-inflammatory [3], antibacterial [4,5], anticonvulsant [6], etc. As explicit examples, RVX-208 and balaglitazone, structurally based on quinazolin-4(3*H*)-one (Figure 1), are now under phase III clinical trials. The compound of RVX-208 is developed for the treatment of cardiovascular diseases and lipid metabolism disorders [7], while balaglitazone is developed for the treatment of type 2 diabetes [8]. Recently, RVX-208 was found effective to reactivate HIV-1 in latent reservoirs [9], which stimulated our interest to synthesize the compounds bearing a quinazolione core.

**Scheme 1:**
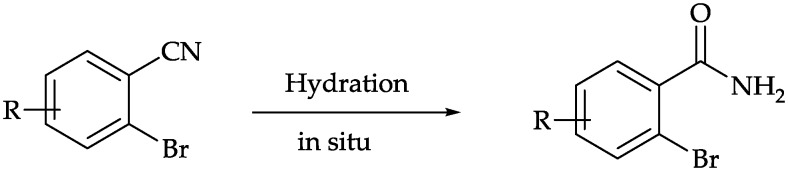
In situ transformation from *o*-bromobenzamide to *o*-bromobenzamide.

A variety of convenient methodologies have been developed for the synthesis of quinazolinones [10,11,12,13]. *o*-Aminobenzamides [14,15,16,17,18,19,20,21,22] and *o*-bromobenzamides [12,23,24,25,26,27,28,29,30,31] are the most frequently used starting materials by far, and *o*-Aminobenzoic acid [32], *o*-haloaniline [26,33], and isatoic anhydride [34] were also introduced. Alternatively, *o*-aminobenzonitrile [35,36,37] was applied to prepare quinazolinones by Wu and co-workers [35], considering that the variation of *o*-aminobenzonitriles is much more available than *o*-aminobenzamides. Similarly, *o*-bromobenzonitriles can also be transformed into *o*-bromobenzamides in situ, and as mentioned above, *o*-bromobenzamide is one of the most studied starting materials (Scheme 1) to synthesize quinazolinones. From a synthetic point of view, we strived to explore *o*-bromobenzonitriles as an alternative substrate to access quinazolinones for following reasons: (1) with *o*-bromobenzonitrile as an alternative starting material, the scope of substrates could be substantially extended, and thus increase the variety of quinazolinones; (2) in our methodology, transforming *o*-bromobenzonitrile into quinazolinones in one pot can save an extra step (benzonitrile into benzamide [38,39] thus the cost can be reduced; and (3) to the best of our knowledge, the substituted *o*-bromobenzonitriles were usually cheaper than the corresponding *o*-bromobenzamides.

Meanwhile, most of these reactions using DMSO or DMA as solvent, suffered from unpleasant smells in high temperature. In addition, it is hard to remove solvent after the reaction. Langer and co-workers [36] provided a green protocol so that dihydroquinazolinons can be prepared in H_2_O with good yields, whereas quinazolinones can only be obtained by adding oxidant TBHP(tert-butyl hydroperoxide). In the context of green chemistry, water was widely explored as a solvent in various reactions [40,41,42,43], not only due to its low cost, non-toxicity, easy availability, and eco-benign features, but also because the theoretical and practical advantages of water substantially improved the “on-water” reaction [44,45,46]. To date, water has never been used as media to synthesize quinazolinones directly without adding any oxidant. Cognizant of these challenges, we tried to introduce water as reaction media into the synthesis of quinazolinones. To our delight, we finally explored a versatile and practical “on-water” protocol for the quinazolinone preparation from *o*-bromobenzonitriles with comparable yields. Surprisingly, with this protocol, a dihydroquinazolinone skeleton could also be built just under the protection of N_2_. Finally, we successfully synthesized 31 compounds including quinazolinones and dihydroquinazolinons, and 10 (**4aa**, **4ee**, **4eq**, **4ff**, **5aa**, **5ea**, **5eb**, **5ee**, **5eq**, **5fe**) of them were novel compounds. Herein, we would like to present our research towards quinazolinones synthesis in detail.

## 2. Discussion and Results

Preliminary investigation started with the reaction of *o*-bromobenzonitrile **1a** (instead of *o*-bromobenzamide) [27], benzaldehyde **2** and ammonia **3** under 100 °C in the present of CuBr, l-proline, and Cs_2_CO_3_ for 24 h. The expected product 2-phenylquinazolin-4(3*H*)-one **4aa** was obtained (Entry 1, Table 1). The reaction conditions could further be optimized. The results collected in Table 1 showed that the reaction performed under 100 °C using Cs_2_CO_3_ as a base provided the highest yield (Entries 1–8, Table 1). The further screening of the catalyst (Entries 9–15, Table 1) showed that copper (Cu (II)) salts gave higher yields of product **4aa** (Entries 12 and 14, Table 1) than Cu (I) in this case, being totally different from the reported results [11,12,25,29,31].

With the catalyst CuCl_2_, solvent effects were examined (Entries 16–21, Table 1) and H_2_O was successfully introduced. Without any oxidant, the “on-water” reaction provided compound 4aa with yield of 75% (Entry 18, Table 1), much higher than using DMF and DMA media. Further attempts to increase the yield by adding DMSO or PEG (polyethylene glycol) in water as a co-solvent failed (Entries 19–20, Table 1). Additionally, air was found to be important for the fusion of quinazolinone core, as only traces of the product formed when the transformation was conducted under the protection of N_2_ (Entry 22, Table 1). Based on these results, we can conclude that air and H_2_O are vital to promote the formation of quinazolinone. Therefore, we obtained the optimal reaction conditions (CuCl_2_ (0.1 mmol), Cs_2_CO_3_ (2 mmol), l-proline (0.2 mmol), H_2_O (2 mL)) for the condensation of *o*-bromobenzonitriles, aldehydes, and aqueous ammonia toward quinazolinones, which are highlighted in Table 1 (Entry 18).

With such an eco-friendly protocol in hand, the workup procedure was simple on account of water. Thereupon, it is necessary to investigate the scope and limitation to explore the versatility of this protocol. Before that, other *o*-halobenzonitriles (Entries 2–4, Table 2) were tested and the results collected in Table 2 showed that *o*-bromobenzonitrile is the most active substrate for this reaction. Various aryl aldehydes **2** were firstly evaluated under standard reaction conditions (Table 2). Generally, most of them were well tolerated and successfully transformed into the corresponding products with moderate to good yields. The electro-donating groups (-Me, -OMe) made a positive influence for the reaction and resulted in higher yields (**4ad**, **4ae**, **4af**, Table 2) than electro-withdrawing counterparts (**4ab**, **4ac**, **4ai**, Table 2). It is noteworthy that almost no steric effect was observed for benzaldehyde, and *o*-methoxybenzaldehyde **2f** even gave a higher yield up to 83% (**4af**, Table 2) than *p*-methoxyone (**4ae**, Table 2). Then we extended this protocol to substituted *o*-bromobenzonitriles.

The electro-withdrawing group on *o*-bromobenzonitrile (Entries 20–23, Table 2) was beneficial to the transformation and gave higher yields of corresponding products than those electro-donating group on *o*-bromobenzonitrile (Entries 24–28, Table 2). Therefore, we got an excellent yield of 92% (**4ef**, Table 2) when 2-bromo-5-fluorobenzonitrile reacted with 2-methoxylbenzaldehyde, which further proved that electro-donating benzaldehydes, especially 2-methoxylbenzaldehyde, were more active than electro-withdrawing benzaldehydes.

Meanwhile, dihydroquinazolinones **5** were isolated as the side products in these reactions. We then repeated the model reaction under the protection of N_2_ (Entry 22, Table 1), after which it failed to produce the compound **4aa**. 2,3-Dihydro-2-phenylquinazolin-4(1*H*)-one **5aa** was obtained and yielded 74%. This discovery suggested that 2-arylquinazolin-4(3*H*)-one **4** and 2,3-dihydro-2-arylquinazolin-4(1*H*)-one 5 can be selectively prepared from o-bromobenzonitrile by controlling the air.

With this result, other substituted benzaldehydes were subsequently employed to prepare 2,3-dihydro-2-arylquinazolin-4(1*H*)-ones **5**. The selected substituted benzaldehydes were smoothly transformed into corresponding expected products with good to excellent yields (Table 3). Unexpectedly, an opposite result was observed compared to the synthesis of 2-arylquinazolin-4(3*H*)-ones **4**. The electro-withdrawing group (4-Cl) helped to improve the yield up to 96% (5ab), while the electro-donated group substituted benzaldehyde gave relatively lower yields (**5ae**, **5af**, Table 3). Especially, *o*-methoxylbenzaldehyde **2f**, which have provided 2-(2-methoxyphenyl)quinazolin-4(3*H*)-one 4af in high yield up to 83%, resulted in the lowest yield of 2,3-dihydro-2-(2-methoxyphenyl)-quinazolin-4(1*H*)-one 5af (54%) under N_2_ in this case. This interesting observation partially manifested that the electron-rich benzaldehyde is good to form 2-arylquinazolin-4(3*H*)-ones **4**, while electron-deficient benzaldehyde tends to produce 2,3-dihydro-2-aryl quinazolin-4(1*H*)-ones **5**.

Therefore, 2-bromo-5-fluorobenzonitrile as well as 2-bromo-5-methylbenzonitrile was treated with benzaldehydes and aqueous ammonia under the the protection of N_2_ with standard reaction conditions (**5ea**–**5fe**, Table 3). Most of them were smoothly transformed into the expected dihydroxyl- products in good yields.

**Scheme 2:**
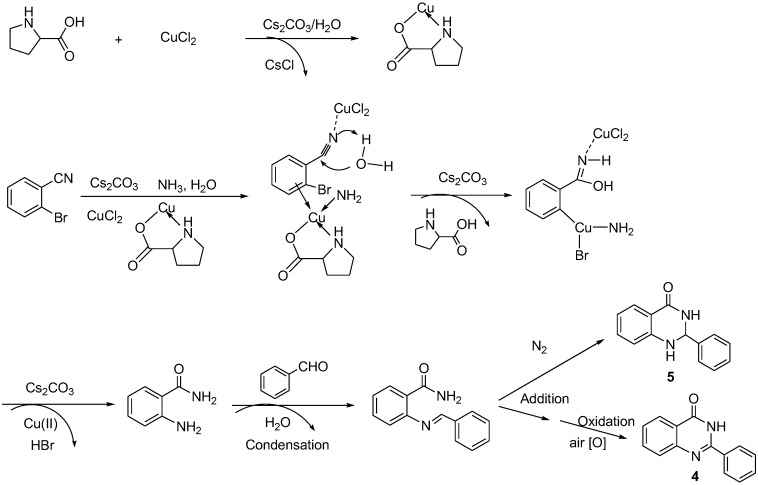
Proposed mechanism.

According to the results and similar reactions [12,13,24,32], a possible mechanism was proposed and outlined in Scheme 2. In the presence of Cs_2_CO_3_, the coordination of L-proline with CuCl_2_ helped to promote the subsequent Ullmann-type reaction to form 2-amino, while oxidation from -CN to -CONH_2_ occurred simultaneously in the presence of CuCl_2_, base, and H_2_O, so that intermediate 2-aminobenzamide was generated. Actually, 2-aminobenzamide was detected as intermediate during the reaction, andthen condensation and addition occurred on 2-aminobenzamide and benzaldehyde. 2,3-Dihydro-2-aryl quinazolin-4(1*H*)-one 5 was produced under the protection of N_2_, and when the reaction was exposed to air after the starting materials were stirred in sealed tubes at 100 °C for 12 h, oxidation occurred further to afford products 2-aryl quinazolin-4(3*H*)-one 4.

**Scheme 3:**
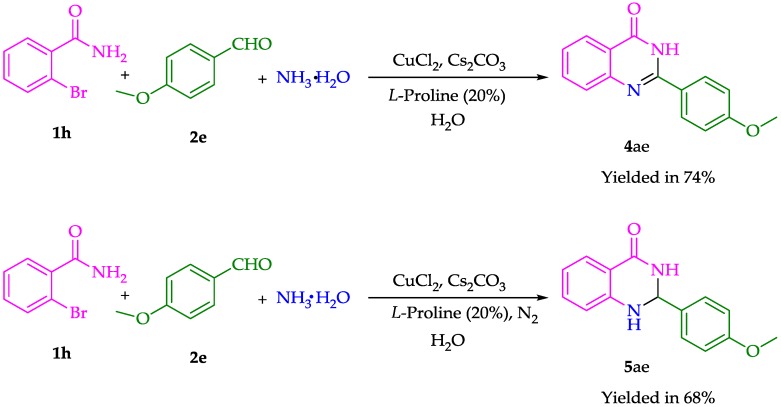
The “on-water” reaction starting from *o*-bromobenzamide.

Although at the outset of this work we only expected to develop an “on-water” protocol towards quinazolinones from *o*-bromobenzonitrile **1a**, the explicit outcome above proved the versatility of this protocol. Then other commonly used substrates were evaluated for this protocol. *o*-Bromobenzamide **1h** was first reacted with *p*-methoxylbenzaldehyde and ammonia under the standard conditions. As outlined in Scheme 3, 2-(4-methoxyphenyl)-quinazolin-4(3*H*)-one **4ae** and 2,3-dihydro-2-phenylquinazolin-4(1*H*)-one **5ae** were produced smoothly at 74% and 68%, respectively, almost having the same yields with *o*-bromobenzonitrile (73% for **4ae** in Table 2, and 71% for **5ae** in Table 3).

**Scheme 4:**
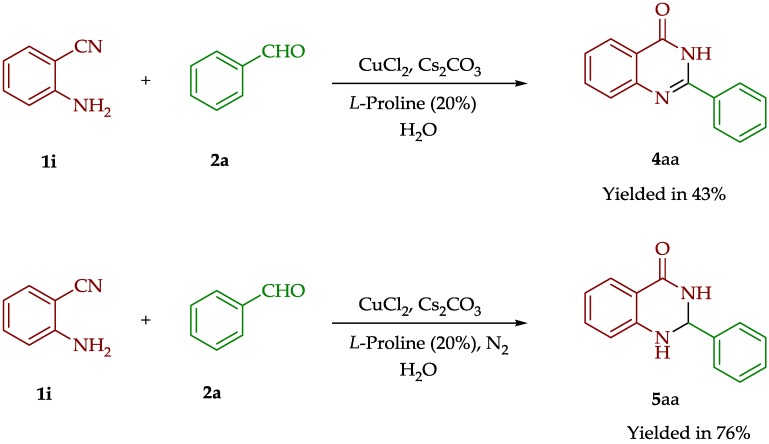
The “on-water” reaction starting from o-aminobenzanitrile.

Subsequently, *o*-aminobenzonitrile **1i** was tested and the results in Scheme 4 showed that the yield of 2-phenylquinazolin-4(3*H*)-one **4aa** was only 43%, while *o*-bromobenzonitrile **1a** yielded in 75% (**4aa**, Table 2). But, when under the protection of N_2_, 76% of the yield of 2,3-dihydro-2-phenylquinazolin-4(1*H*)-one **5aa** was obtained, almost the same with the result from *o*-bromobenzonitrile **1a** (**5aa**, Table 3). Obviously, *o*-aminobenzonitrile **1i** was less active than o-bromobenzonitrile **1a** for the synthesis of quinazolin-4(3*H*)-one, which explained why 2,3-dihydro-quinazolin-4(1*H*)-ones were the only products listed in the literature reported by Langer and co-workers [36], wherein oxidant TBHP had to be added in addition to help the formation of quinazolin-4(3*H*)-one.

**Scheme 5:**
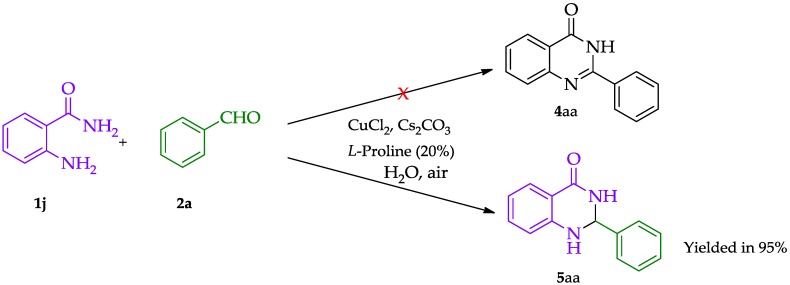
“On-water” reaction starting from o-aminobenzamide.

This interesting finding prompted us to undertake the reaction starting from *o*-aminobenzamide **1j** immediately, as shown in Scheme 5. Unexpectedly, oxidized product 2-phenylquinazolin-4(3*H*)-one **4aa** failed to produce, while 2,3-dihydro-2-phenylquinazolin-4(1*H*)-one **5aa** yielded up to 95% without the protection of N_2_. It implies that *o*-aminobenzamide **1j** tends to be transformed into 2,3-dihydro-quinazolin-4(1*H*)-ones **5** with this protocol.

With these results from the extended substrates, we found the newly developed “on-water” protocol was much more flexible for the construction of 2,3-dihydro-quinazolin-4(1*H*)-ones **5** than its oxidized products quinazolin-4(3*H*)-ones **4**. In addition, for the preparation of quinazolin-4(3*H*)-ones **4**, the priority of the commonly used substrates is: *o*-bromobenzonitrile **1a** or *o*-bromobenzamide **1h** > *o*-amino- benzonitrile *1i* > *o*-aminobenzamide **1j**.

## 3. Conclusions

In summary, we have newly developed a versatile and practical “on-water” protocol towards compounds containing a quinazolinone core, and *o*-bromobenzonitrile was explored as an alternative starting material. Water and air were found to be of importance for the formation of oxidized products quinazolin-4(3*H*)-ones **4**, and various aryl aldehydes as well as several substituted *o*-bromobenzonitriles were successfully applied to this protocol and provided the expected quinazolin-4(3*H*)-ones **4**. Moreover, we found that 2,3-dihydro-quinazolin-4(1*H*)-ones **5** could also be obtained when the reaction was carried out under the protection of N_2_, and the subsequent scope investigation resulted in the successful synthesis of various 2,3-dihydro-quinazolin-4(1*H*)-ones **5**. Therefore, we can selectively produce the compounds 2,3-dihydro-quinazolin-4(1*H*)-ones **5** or their oxidized products **4** from *o*-bromobenzonitrile by controlling air. In addition, *o*-bromobenzonitrile, *o*-aminobenzonitrile, and *o*-aminobenzamide were evaluated and all of them could be transformed into 2,3-dihydro-quinazolin-4(1*H*)-ones **5** with good to excellent yields, but *o*-bromobenzonitrile or *o*-bromobenzamide was proved to be the best substrate for the synthesis of the oxidized products quinazolin-4(3*H*)-ones compared with *o*-aminobenzonitrile and *o*-aminobenzamide. With these new findings in mind, further work involving the synthesis of RVX-208 and related structural modifications are ongoing in our group.

## 4. Materials and Methods

### 4.1. General

All chemicals were purchased from commercial suppliers Shanghai Energy Chemical Co., Ltd. (Shanghai, China), Adamas Reagent, Ltd. (Shanghai, China), TCI Industry Co., Ltd. (Shanghai, China), without further purification. All reactions were monitored by TLC (thin-layer chromatography) which was performed on GF254 silica gel glass plates (Qingdao Haiyang Chemical Co. Ltd., Qingdao, Shandong, China). Column chromatography was performed with silica gel (200–300 mesh). All unknown compounds were structurally verified by ^1^H-NMR, ^13^C-NMR and MS, and ^1^H-, and ^13^C-NMR spectra were recorded on a Bruker Advance drx 400 spectrometer (Bruker Bioscience, Billerica, MA, USA) operating at 400 MHz and 100 MHz, respectively. The chemical shifts were reported in ppm and the coupling constant in Hz. Mass Spectrometry analysed for the known compounds by Waters HPLC/ZQ 4000 Thermo Fisher Scientific (Waltham, MA, USA). 

### 4.2. General Procedure for the Synthesis of 2-Phenylquinazolin-4(3H)-one (**4aa**)

To a mixture of 2-Bromobenzonitrile (183.4 mg, 1 mmol), benzaldehyde (210.5 mg, 2 mmol), CuCl_2_ (17.2 mg, 0.1 mmol), Cs_2_CO_3_ (652.2 mg, 2 mmol), and l-proline (23.2 mg, 0.2 mmol) in H_2_O (2 mL) was added 27% aqueous ammonia (1 mL) in a tube under air atmosphere. Then the tube was sealed, and the mixture was stirred at 100 °C for 12 h. Next, the tube was opened to air and the mixture was stirred at 100 °C for another 12 h. After being cooled to room temperature, the resulting mixture was quenched with NH_4_Cl solution and extracted with ethyl acetate. The combined organic layer was washed with brine, and then dried over anhydrous Na_2_SO_4_. The solvent was evaporated under reduced pressure and the crude product was purified by chromatography on silica-gel to afford 2-phenylquinazolin-4(3*H*)-one (**4aa**) in 75% isolated yield. ^1^H-NMR (400 MHz, Chloroform-d) δ11.24 (s, 1H, -NH-), 8.27 (d, *J* = 7.8 Hz, 1H, Ar-H), 8.16 (**dd**, *J* = 6.6, 3.0 Hz, 2H, Ar-H), 7.82–7.71 (m, 2H, Ar-H), 7.57–7.49 (m, 3H, Ar-H), 7.48–7.41 (m, 1H, Ar-H). ^13^C-NMR (100 MHz, Chloroform-d) δ151.60, 134.87, 132.77, 131.64, 129.05, 127.97, 127.25, 126.79, 126.34, 120.84. HRMS (ESI) calcd for C_14_H_11_N_2_O [M + H]^+^: 223.0866. Found: 223.0865.

### 4.3. General Procedure for the Synthesis of 2-Phenyl-2,3-dihydroquinazolin-4(1H)-one (**5aa**)

2-bromobenzonitrile (182.3 mg, 1 mmol), benzaldehyde (213.6 mg, 2 mmol), CuCl_2_ (17.1 mg, 0.1 mmol), Cs_2_CO_3_ (651.3 mg, 2 mmol) and l-proline 23.4 mg, 0.2 mmol) in H_2_O (2 mL) were added into a tube and stirred. Remove the air inside the tube under the reduced pressure and flush with N_2_, and protected the starting materials under N_2_. 27 % aqueous ammonia (1 mL) was added into the reaction mixture. The tube was then sealed and the mixture stirred at 100 °C for 24 h. After cooling to room temperature, the resulting mixture was quenched with NH_4_Cl solution and extracted with ethyl acetate. The combined organic layers were washed with brine and then dried over anhydrous Na_2_SO_4_. The solvent was evaporated under reduced pressure and the crude product was purified by chromatography on silica-gel to afford 2-phenyl-2,3-dihydroquinazolin-4(1*H*)-one(5aa) in 74% isolated yield. ^1^H-NMR (400 MHz, Chloroform-d) *δ* 7.97 (d, *J* = 7.8 Hz, 1H, Ar-H), 7.67–7.56 (m, 2H, Ar-H), 7.55–7.41 (m, 3H, Ar-H), 7.36 (t, *J* = 7.7 Hz, 1H, Ar-H), 6.93 (t, *J* = 7.5 Hz, 1H, Ar-H), 6.70 (d, *J* = 8.0 Hz, 1H, Ar-H), 5.93 (s, 1H, -CH-), 5.80 (s, 1H, -NH-), 4.42 (s, 1H, -NH-). ^13^C-NMR (101 MHz, DMSO) *δ* 163.98, 148.27, 142.04, 133.70, 128.85, 128.72, 127.75, 127.26, 117.51, 115.36, 114.80, 66.96. HRMS (ESI) calcd for C_14_H_13_N_2_O [M + H]^+^: 225.1022. Found: 225.1021.

### 4.4. General Procedure for the Synthesis of 2-Phenylquinazolin-4(3H)-one (**4aa**) with Scheme 3

*o*-Aminobenzanitrile (119.8 mg, 1 mmol), benzaldehyde (217.8 mg, 2 mmol), CuCl_2_ (17.6 mg, 0.1 mmol), Cs_2_CO_3_ (652.3 mg, 2 mmol), and l-proline 23.6 mg, 0.2 mmol) in H_2_O (2 mL) was added in a 5 mL reaction bottle. Then the mixture was stirred at 100 °C for 48 h. After cooling to room temperature, the resulting mixture was quenched with NH_4_Cl solution and extracted with ethyl acetate. The combined organic layers were washed with brine and then dried over anhydrous Na_2_SO_4_. The solvent was evaporated under reduced pressure and the crude product was purified by chromatography on silica-gel to afford 2-phenylquinazolin-4(3*H*)-one (**4aa**) in 43% isolated yield.

### 4.5. General Procedure for the Synthesis of 2-Phenyl-2,3-dihydroquinazolin-4(1H)-one (**5aa**) with Scheme 3

*o*-Aminobenzanitrile (120.8 mg, 1 mmol), benzaldehyde (216.7 mg, 2 mmol), CuCl_2_ (17.3 mg, 0.1 mmol), Cs_2_CO_3_ (653.7 mg, 2 mmol), and l-proline 23.3 mg, 0.2 mmol) in H_2_O (2 mL) was added in a 5 mL reaction bottle under nitrogen. Then the mixture was stirred at 100 °C for 24 h. After cooling to room temperature, the resulting mixture was quenched with NH_4_Cl solution and extracted with ethyl acetate. The combined organic layers were washed with brine and then dried over anhydrous Na_2_SO_4_. The solvent was evaporated under reduced pressure and the crude product was purified by chromatography on silica-gel to afford 2-phenyl-2,3-dihydroquinazolin-4(1*H*)-one (**5aa**) in 76% isolated yield.

### 4.6. General Procedure for the Synthesis of 2-Phenyl-2,3-dihydroquinazolin-4(1H)-one (**5aa**) with Scheme 4

*o*-Aminobenzamide (139.1 mg, 1 mmol), benzaldehyde (209.9 mg, 2 mmol), CuCl_2_ (17.6 mg, 0.1 mmol), Cs_2_CO_3_ (657.0 mg, 2 mmol) and l-proline 23.6 mg, 0.2 mmol) in H_2_O (2 mL) was added in a 5 mL reaction bottle. Then the mixture was stirred at 100 °C for 14 h. After cooling to room temperature, the resulting mixture was quenched with NH_4_Cl solution and extracted with ethyl acetate. The combined organic layers were washed with brine and then dried over anhydrous Na_2_SO_4_. The solvent was evaporated under reduced pressure and the crude product was purified by chromatography on silica-gel to afford 2-phenyl-2,3-dihydroquinazolin-4(1*H*)-one (**5aa**) in 95% isolated yield.

Experimental procedures and analytical data of all compounds (^1^H NMR and ^13^C NMR), copy of the ^1^H NMR and ^13^C NMR and data are available in the Supplementary Materials.

## Figures and Tables

**Table 1:**
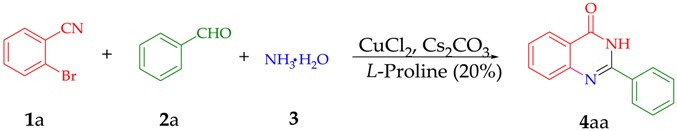
Conditional optimization for the condensation of o-bromobenzonitrile **1a** with benzaldehyde **2a** ^a^.

Entry	Catal. (10 mol %)	Base (2 eqv.)	Solvent	Temp (°C)	Yield (%) ^b^
1	CuBr	Cs_2_CO_3_	DMSO	100	54
2	CuBr	Cs_2_CO_3_	DMSO	80	49
3	CuBr	Cs_2_CO_3_	DMSO	120	42
4	CuBr	-	DMSO	100	38
5	CuBr	K_2_CO_3_	DMSO	100	47
6	CuBr	K_3_PO_4_	DMSO	100	51
7	CuBr	NaOH	DMSO	100	37
8	CuBr	Et_3_N	DMSO	100	21
9	CuCl	Cs_2_CO_3_	DMSO	100	54
10	-	Cs_2_CO_3_	DMSO	100	16
11	CuI	Cs_2_CO_3_	DMSO	100	33
12	CuCl_2_	Cs_2_CO_3_	DMSO	100	62
13	FeCl_3_	Cs_2_CO_3_	DMSO	100	28
14	CuO	Cs_2_CO_3_	DMSO	100	63
15	ZnCl_2_	Cs_2_CO_3_	DMSO	100	45
16	CuCl_2_	Cs_2_CO_3_	DMF	100	24
17	CuCl_2_	Cs_2_CO_3_	DMA	100	41
18	CuCl_2_	Cs_2_CO_3_	H_2_O	100	75
19	CuCl_2_	Cs_2_CO_3_	H_2_O/DMSO (4:1)	100	47
20	CuCl_2_	Cs_2_CO_3_	H_2_O/PEG400 (4:1)	100	55
21	CuCl_2_	Cs_2_CO_3_	DMSO	100	67
22	CuCl_2_	Cs_2_CO_3_	H_2_O	100	Trace ^c^

^a^ Reaction conditions: *o*-bromobenzonitrile **1a** (1 mmol), benzaldehyde **2a** (2 mmol), aqueous ammonia **3** (27%, 1 mL), catalyst (0.1 mmol), base (2 mmol), l-proline (0.2 mmol), and solvent (2 mL), heated, sealed, and stirred for 12 h, then refluxed under air for 12 h. ^b^ Isolated yield. ^c^ Reaction was conducted under protection of N_2_.

**Table 2:**
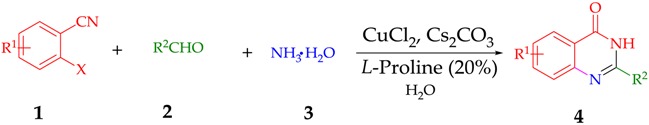
The scope and limitation for the synthesis of 2-aryl quinazolin-4(3*H*)-one **4** ^a^.

Entry	X	R^1^	R^2^	Product	Yield ^b^
1	Br	-	C_6_H_5_	4aa	75%
2	F	-	C_6_H_5_	4aa	0
3	Cl	-	C_6_H_5_	4aa	21%
4	I	-	C_6_H_5_	4aa	17%
5	Br	-	4-ClC_6_H_5_	4ab	44%
6	Br	-	2-ClC_6_H_5_	4ac	41%
7	Br	-	4-CH_3_C_6_H_5_	4ad	71%
8	Br	-	4-MeOC_6_H_5_	4ae	73%
9	Br	-	2-MeOC_6_H_5_	4af	83%
10	Br	-	4-HOC_6_H_5_	4ag	42%
11	Br	-	2-HOC_6_H_5_	4ah	0
12	Br	-	4-CF_3_C_6_H_5_	4ai	27%
13	Br	-	4-N(CH_3_)_2_C_6_H_5_	4aj	30%
14	Br	-	naphthalene	4ak	66%
15	Br	-	4-Pyridine	4al	48%
16	Br	-	2-Pyridine	4am	0
17	Br	-	2-furan	4an	0
18	Br	-	2-thiophene	4ao	0
19	Br	-	2-pyrrole	4ap	27%
20	Br	5-F	C_6_H_5_	4ea	60%
21	Br	5-F	4-MeOC_6_H_5_	4ee	73%
22	Br	5-F	2-MeOC_6_H_5_	4ef	92%
23	Br	5-F	3-MeO-4-HOC_6_H_5_	4eq	60%
24	Br	5-CH_3_	C_6_H_5_	4fa	63%
25	Br	5-CH_3_	4-ClC_6_H_5_	4fb	47%
26	Br	5-CH_3_	4-MeOC_6_H_5_	4fe	51%
27	Br	5-CH_3_	2-MeOC_6_H_5_	4ff	62%
28	Br	5-MeO	4-MeOC_6_H_5_	4ge	67%

^a^ Reaction conditions: substituted o-halobenzonitrile **1** (1 mmol), aryl aldehyde **2** (2 mmol), aqueous ammonia **3** (27%, 1 mL), CuCl_2_ (0.1 mmol), Cs_2_CO_3_ (2 mmol), L-proline (0.2 mmol), and H_2_O (2 mL), heated, sealed, and stirred for 12 h, then refluxed under air for 12 h. ^b^ Isolated yield.

**Table 3:**
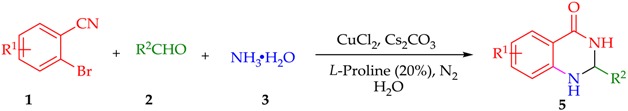
The scope and limitation for the synthesis of 2,3-dihydro-2-aryl quinazolin-4(1*H*)-one **5** ^a^.

Entry	R^1^	R^2^	Product	Yield ^b^
1	-	C_6_H_5_	**5aa**	74%
2	-	4-ClC_6_H_5_	**5ab**	96%
3	-	4-MeOC_6_H_5_	**5ae**	71%
4	-	2-MeOC_6_H_5_	**5af**	54%
5	5-F	C_6_H_5_	**5ea**	39%
6	5-F	4-ClC_6_H_5_	**5eb**	27%
7	5-F	4-MeOC_6_H_5_	**5ee**	53%
8	5-F	2-MeOC_6_H_5_	**5eq**	53%
9	5-CH_3_	C_6_H_5_	**5fa**	77%
10	5-CH_3_	4-ClC_6_H_5_	**5fb**	0
11	5-CH_3_	4-MeOC_6_H_5_	**5fe**	50%

^a^ Reaction conditions: substituted *o*-bromobenzonitrile **1a** (1 mmol), aryl aldehyde **2** (2 mmol), aqueous ammonia **3** (27%, 1 mL), CuCl_2_ (0.1 mmol), Cs_2_CO_3_ (2 mmol), l-proline (0.2 mmol), and H_2_O (2 mL), heated, sealed, and stirred under protection of N_2_ for 24 h. ^b^ Isolated yield.

## Supplementary Materials

The experiment procedure, spectral and analytical data, characterization data including ^1^H-, ^13^C-, and ^19^F-NMR spectra of compounds **1**–**31** are available online.

## References

[1] Safavi M., Ashtari A., Khalili F., Mirfazli S.S., Saeedi M., Ardestani S.K., Rashidi R.P., Barazandeh T.M., Larijani B., Mahdavi M. (2018). Novel quinazolin-4(3*H*)-one linked to 1,2,3-triazoles: Synthesis and anticancer activity. Chem. Biol. Drug Des..

[2] Logé C., Testard A., Thiéry V., Lozach O., Blairvacq M., Robert J.-M., Meijer L., Besson T. (2008). Novel 9-oxo-thiazolo [5, 4-f] quinazoline-2-carbonitrile derivatives as dual cyclin-dependent kinase 1 (CDK1)/glycogen synthase kinase-3 (GSK-3) inhibitors: Synthesis, biological evaluation and molecular modeling studies. Eur. J. Med. Chem..

[3] Moussa G., Alaaeddine R., Alaeddine L.M., Nassra R., Belal A.S.F., Ismail A., El-Yazbi A.F., Abdel-Ghany Y.S., Hazzaa A. (2018). Novel click modifiable thioquinazolinones as anti-inflammatory agents: Design, synthesis, biological evaluation and docking study. Eur. J. Med. Chem..

[4] Zhan X., Xu Y., Qi Q., Wang Y., Shi H., Mao Z. (2018). Synthesis, cytotoxic, and antibacterial evaluation of quinazolinone derivatives with substituted amino moiety. Chem. Biodivers..

[5] Liao B.-L., Pan L.W., Pan Y.-J., Zhang W. (2018). Four natural compounds were separated from folium isatidis: Crystal structures and antibacterial activity. Chem. Biodivers..

[6] Wolfe J.F., Rathman T.L., Sleevi M.C., Campbell J.A., Greenwood T.D. (1990). Synthesis and anticonvulsant activity of some new 2-substituted 3-aryl-4(3*H*)-quinazolinones. J. Med. Chem..

[7] Ghosh G.C., Bhadra R., Ghosh R.K., Banerjee K., Gupta A. (2017). RVX 208: A novel BET protein inhibitor, role as an inducer of apo A.-I./HDL and beyond. Cardiovasc. Ther..

[8] Larsen P.J., Lykkegaard K., Larsen L.K., Fleckner J., Sauerberg P., Wassermann K., Wulff E.M. (2008). Dissociation of antihyperglycaemic and adverse effects of partial perioxisome proliferator-activated receptor (PPAR-γ) agonist balaglitazone. Eur. J. Pharmacol..

[9] Zhang X.-X., Lin J., Liang T.-Z., Duan H., Tan X.-H., Xi B.-M., Li L., Liu S.-W. (2018). The BET bromodomain inhibitor apabetalone induces apoptosis of latent HIV-1 reservoir cells following viral reactivation. Acta Pharmacol. Sin..

[10] Connolly D.J., Cusack D., O’Sullivan T.P., Guiry P.J. (2005). Synthesis of quinazolinones and quinazolines. Tetrahedron.

[11] He L., Li H., Chen J., Wu X.-F. (2014). Recent advances in 4(3*H*)-quinazolinone syntheses. RSC Adv..

[12] Upadhyaya K., Thakur R.K., Shukla S.K., Tripathi R.P. (2016). One-pot copper(i)-catalyzed ligand/base-free tandem cyclooxidative synthesis of quinazolinones. J. Org. Chem..

[13] Laclef S., Harari M., Godeau J., Schmitz-Afonso I., Bischoff L., Hoarau C., Levacher V., Fruit C., Besson T. (2015). Ligand-free Pd-catalyzed and copper-assisted C–H arylation of quinazolin-4-ones with aryl iodides under microwave heating. Org. Lett..

[14] Cheng R., Guo T., Zhang-Negrerie D., Du Y., Zhao K. (2013). One-pot synthesis of quinazolinones from anthranilamides and aldehydes via p-toluenesulfonic acid catalyzed cyclocondensation and phenyliodine diacetate mediated oxidative dehydrogenation. Synthesis.

[15] Ge W., Zhu X., Wei Y. (2013). Iodine-catalyzed oxidative system for cyclization of primary alcohols with o-aminobenzamides to quinazolinones using DMSO as the oxidant in dimethyl carbonate. RSC Adv..

[16] Jiang X., Tang T., Wang J.-M., Chen Z., Zhu Y.-M., Ji S.-J. (2014). Palladium-Catalyzed One-Pot Synthesis of Quinazolinones via tert-Butyl Isocyanide Insertion. J. Org. Chem..

[17] Kim N.Y., Cheon C.-H. (2014). Synthesis of quinazolinones from anthranilamides and aldehydes via metal-free aerobic oxidation in DMSO. Tetrahedron Lett..

[18] Sharif M., Opalach J., Langer P., Beller M., Wu X.-F. (2014). Oxidative synthesis of quinazolinones and benzothiadiazine 1,1-dioxides from 2-aminobenzamide and 2-aminobenzenesulfonamide with benzyl alcohols and aldehydes. RSC Adv..

[19] Wu X.-F., He L., Neumann H., Beller M. (2013). Palladium-catalyzed carbonylative synthesis of quinazolinones from 2-aminobenzamide and aryl bromides. Chem.-Eur. J..

[20] Xu W., Zhu X.-R., Qian P.-C., Zhang X.-G., Deng C.-L. (2016). Copper-catalyzed tandem reaction of 2-aminobenzamides with tertiary amines for the synthesis of quinazolinone derivatives. Synlett.

[21] Zhan D., Li T., Wei H., Weng W., Ghandi K., Zeng Q. (2013). A recyclable CuO-catalyzed synthesis of 4(3*H*)-quinazolinones. RSC Adv..

[22] Li F., Lu L., Liu P. (2016). Acceptorless dehydrogenative coupling of o-aminobenzamides with the activation of methanol as a C1 source for the construction of quinazolinones. Org. Lett..

[23] Hu B.-Q., Wang L.-X., Xiang J.-F., Yang L., Tang Y.-L. (2015). Cu(II)-catalyzed domino reaction of 2-halobenzamide and arylmethanamine to construct 2-aryl quinazolinone. Chin. Chem. Lett..

[24] Huang C., Fu Y., Fu H., Jiang Y., Zhao Y. (2008). Highly efficient copper-catalyzed cascade synthesis of quinazoline and quinazolinone derivatives. Chem. Commun..

[25] Kotipalli T., Kavala V., Janreddy D., Bandi V., Kuo C.-W., Yao C.-F. (2016). Synthesis of 2,3-disubstituted quinazolinone derivatives through copper catalyzed C–H amidation reactions. Eur. J. Org. Chem..

[26] Larksarp C., Alper H. (2000). Palladium-catalyzed cyclocarbonylation of o-iodoanilines with heterocumulenes: Regioselective preparation of 4(3*H*)-quinazolinone derivatives. J. Org. Chem..

[27] Xu L., Jiang Y., Ma D. (2012). Synthesis of 3-substituted and 2,3-disubstituted quinazolinones via cu-catalyzed aryl amidation. Org. Lett..

[28] Xu W., Fu H. (2011). Amino acids as the nitrogen-containing motifs in copper-catalyzed domino synthesis of n-heterocycles. J. Org. Chem..

[29] Xu W., Jin Y., Liu H., Jiang Y., Fu H. (2011). Copper-catalyzed domino synthesis of quinazolinones via ullmann-type coupling and aerobic oxidative C.−H. amidation. Org. Lett..

[30] Yu L., Wang M., Li P., Wang L. (2012). Fe_3_O_4_ nanoparticle-supported copper(I): Magnetically recoverable and reusable catalyst for the synthesis of quinazolinones and bicyclic pyrimidinones. Appl. Organomet. Chem..

[31] Guo S., Li Y., Tao L., Zhang W., Fan X. (2014). Rapid assembly of quinazolinone scaffold via copper-catalyzed tandem reaction of 2-bromobenzamides with aldehydes and aqueous ammonia: Application to the synthesis of the alkaloid tryptanthrin. RSC Adv..

[32] Liu X., Fu H., Jiang Y., Zhao Y. (2009). A simple and efficient approach to quinazolinones under mild copper-catalyzed conditions. Angew. Chem. Int. Ed..

[33] He L., Li H., Neumann H., Beller M., Wu X.-F. (2014). Highly efficient four-component synthesis of 4(3 *H*)-quinazolinones: Palladium-catalyzed carbonylative coupling reactions. Angew. Chem. Int. Ed..

[34] Zeng L.-Y., Cai C. (2010). Iodine: Selectively promote the synthesis of mono substituted quinazolin-4(3*H*)-ones and 2,3-dihydroquinazolin-4(1*H*)-ones in one-pot. J. Heterocycl. Chem..

[35] Li H., He L., Neumann H., Beller M., Wu X.-F. (2014). Cascade synthesis of quinazolinones from 2-aminobenzonitriles and aryl bromides via palladium-catalyzed carbonylation reaction. Green Chem..

[36] Wu X.-F., Oschatz S., Block A., Spannenberg A., Langer P. (2014). Base mediated synthesis of 2-aryl-2,3-dihydroquinazolin-4(1*H*)-ones from 2-aminobenzonitriles and aromatic aldehydes in water. Org. Biomol. Chem..

[37] Wu X.-F., Oschatz S., Sharif M., Beller M., Langer P. (2014). Palladium-catalyzed carbonylative synthesis of N-(2-cyanoaryl)benzamides and sequential synthesis of quinazolinones. Tetrahedron.

[38] Schmid T.E., Gómez-Herrera A., Songis O., Sneddon D., Révolte A., Nahra F., Cazin C.S. (2015). Selective NaOH-catalysed hydration of aromatic nitriles to amides. Catal. Sci. Technol..

[39] Sahnoun S., Messaoudi S., Peyrat J.-F., Brion J.-D., Alami M. (2012). Cs2CO3 in pyrrolidinone promoted hydration of functionalized (hetero)aryl nitriles under metal-free conditions. Tetrahedron Lett..

[40] Zeng L.-Y., Liu T., Yang J., Yang Y., Cai C., Liu S. (2017). “On-Water” facile synthesis of novel pyrazolo[3,4-b]pyridinones possessing anti-influenza virus activity. ACS Comb. Sci..

[41] Yadong F., Yudong L., Guolin C., Lianhui W., Xiuling C. (2015). Copper-catalyzed synthesis of 2-arylquinazolinones from 2-arylindoles with amines or ammoniums. J. Org. Chem..

[42] Mitsuaki Y., Yukari N., Seiya H., Akira I. (2016). One-pot synthesis of polyhydropyrido [1, 2-a] indoles and tetracyclic quinazolinones from 2-arylindoles using copper-mediated oxidative tandem reactions. Tetrahedron.

[43] Khan I., Zaib S., Batool S., Abbas N., Ashraf Z., Iqbal J., Saeed A. (2016). Quinazolines and quinazolinones as ubiquitous structural fragments in medicinal chemistry: An update on the development of synthetic methods and pharmacological diversification. Bioorgan. Med. Chem..

[44] Butler R.N., Coyne A.G. (2010). Water: Nature’s reaction enforcer—Comparative effects for organic synthesis “in-water” and “on-water”. Chem. Rev..

[45] Dam B., Nandi S., Pal A.K. (2014). An efficient ‘on-water’ synthesis of 1,4-dihydropyridines using Fe_3_O_4_@SiO_2_ nanoparticles as a reusable catalyst. Tetrahedron Lett..

[46] Saikia B., Boruah P.R., Ali A.A., Sarma D. (2015). ‘On-water’ organic synthesis: A. green, highly efficient, low cost and reusable catalyst system for biaryl synthesis under aerobic conditions at room temperature. RSC Adv..

