# Supplementary material for: “On-Water” Synthesis of Quinazolinones and Dihydroquinazolinones Starting from o-Bromobenzonitrile
Source: Molecules. 2018 Sep 12;23(9):2325. doi: 10.3390/molecules23092325 (PMC6225144; doi:10.3390/molecules23092325)
Supplement: Supplementary file 1 [file molecules-23-02325-s001.pdf]

Table of contents

1. General Methods S2

2. Experiment procedure.. S2

3. Spectral and Analytical Data of products S3

4. NMR Spectrums for comounds 1-31 (Figure S1- S81) S12

Formatted: Font: (Default) Times New Roman, (Asian) +Headings Asian (宋体)

Formatted: Font: (Default) Times New Roman

Field Code Changed

Formatted: Font: (Default) Times New Roman, 10.5 pt, N Bold, Font color: Auto

Formatted: Font: (Default) Times New Roman, 10.5 pt, N Bold, Font color: Auto

Formatted: Font: (Default) Times New Roman

Formatted: Font: (Default) Times New Roman, 10.5 pt, N Bold, Font color: Auto

1. General Methods

All the chemicals were purchased from the commercial suppliers without further purification. All reactions were monitored by TLC, analytical thin-layer chromatography was performed on GF254 silica gel glass plates. Column chromatography was performed with silica gel (200-300 mesh). All unknown compounds were structurally verified by ^{1}H NMR, ^{13}C NMR and MS, and ^{1}H, ^{13}C NMR spectra were recorded on a Bruker Advance drx 400 spectrometer operating at 400MHz and 100 MHz, respectively, the chemical shifts are reported in ppm and the coupling constant in Hz. MS analysed for the known compounds by Waters HPLC/ZQ 4000.

2. Experiment procedure

2.1 General procedure for the synthesis of 2-phenylquinazolin-4(3H)-one(4aa):

To a mixture of 2-Bromobenzonitrile (183.4 mg, 1 mmol), benzaldehyde (210.5 mg, 2 mmol), $CuCl_2$ (17.2 mg, 0.1 mmol), Cs_2CO_3 (652.2 mg, 2mmol), and L-proline (23.2 mg, 0.2 mmol) in H_2O (2 mL) was added 27% aqueous ammonia (1 mL) in a tube under air atmosphere. Then the tube was sealed, and the mixture was stirred at 100℃ for 12 h. Next, the tube was opened to air and the mixture was stirred at 100 ℃ for another 12 h. After being cooled to room temperature, the resulting mixture was quenched with NH_4Cl solution and extracted with ethyl acetate. The combined organic layer was washed with brine, and then dried over anhydrous Na_2SO_4. The solvent was evaporated under reduced pressure and the crude product was purified by chromatography on silica-gel to afford 2-phenylquinazolin-4(3H)-one (**4aa**) in 75% isolated yield. ^{1}H NMR (400 MHz, $CDCl_3$-*d*) δ 11.24 (s, 1H，-NH-), 8.27 (d, *J* = 7.8 Hz, 1H, Ar-H), 8.16 (dd, *J* = 6.6, 3.0 Hz, 2H, Ar-H), 7.82 – 7.71 (m, 2H, Ar-H), 7.57 – 7.49 (m, 3H, Ar-H), 7.48 – 7.41 (m, 1H, Ar-H). ^{13}C NMR (101 MHz, $CDCl_3$-*d*) δ 151.60, 134.87, 132.77, 131.64, 129.05, 127.97, 127.25, 126.79, 126.34, 120.84. HRMS (ESI) calcd for $C_{14}H_{11}N_2O$ $[M+H]^+$: 223.0866. Found: 223.0865.

2.2 General procedure for the synthesis of 2-phenyl-2,3-dihydroquinazolin- 4(1H)-one(5aa):

2-bromobenzonitrile (182.3 mg, 1 mmol), benzaldehyde (213.6 mg, 2 mmol), $CuCl_2$ (17.1 mg, 0.1 mmol), Cs_2CO_3 (651.3 mg, 2 mmol) and L-proline 23.4 mg, 0.2 mmol) in H_2O (2 mL) were added into a tube and stirred. Remove the air inside the tube under the reduced pressure and flush with N_2, repeat this operation 3 times and protected the starting materials under N_2. 27% of Aqueous ammonia (1 mL) was added into the reaction mixture under nitrogen. The tube was then sealed and the mixture was stirred at 100 ℃for 24 hours. After cooling to room temperature, the resulting mixture was quenched with NH_4Cl solution and extracted with ethyl acetate. The combined organic layers were washed with brine and then dried over anhydrous Na_2SO_4.The solvent was evaporated under reduced pressure and the crude product was purified by chromatography on silica-gel to afford 2-phenyl-2,3-dihydroquinazolin-4(1H)-one(5aa) in 74% isolated yield. ^{1}H NMR (400 MHz, $CDCl_3$-*d*) δ 7.97 (d, J = 7.8 Hz, 1H, Ar-H), 7.67 – 7.56 (m, 2H, Ar-H), 7.55 – 7.41 (m, 3H, Ar-H), 7.36 (t, J = 7.7 Hz, 1H, Ar-H), 6.93 (t, J = 7.5 Hz, 1H, Ar-H), 6.70 (d, J = 8.0 Hz, 1H, Ar-H), 5.93 (s, 1H, -CH-), 5.80 (s, 1H, -NH-), 4.42 (s, 1H, -NH-).^{13}C NMR (101 MHz, DMSO) δ 163.98, 148.27, 142.04, 133.70, 128.85, 128.72, 127.75, 127.26, 117.51, 115.36, 114.80, 66.96. HRMS (ESI) calcd for $C_{14}H_{13}N_2O$$[M+H]^+$: 225.1022. Found: 225.1021.

Formatted: Font: (Asian) +Headings Asian (宋体)
Formatted: Font: (Asian) +Headings Asian (宋体)
Formatted: Font: (Asian) +Body Asian (宋体)
Formatted: Font: (Default) 宋体, (Asian) 宋体, Not Bold
Formatted: Font: (Default) 宋体, (Asian) 宋体, Not Bold
Formatted: Font: Not Bold, Font color: Auto
Formatted: Font: Italic
Formatted: Font: Italic
Formatted: Font: Not Bold, Font color: Auto
Commented [A1]:

2.3 General procedure for the synthesis of 2-phenylquinazolin-4(3H)-one (4aa) with Scheme 3:

o-aminobenzanitrile (119.8 mg, 1 mmol), benzaldehyde (217.8 mg, 2 mmol), $CuCl_2$ (17.6 mg, 0.1 mmol), Cs_2CO_3 (652.3 mg, 2 mmol) and L-proline 23.6 mg, 0.2 mmol) in H_2O (2 mL) was added in a 5 ml reaction bottle. The the mixture was stirred at 100 ℃ for 48 hours. After cooling to room temperature, the resulting mixture was quenched with NH_4Cl solution and extracted with ethyl acetate. The combined organic layers were washed with brine and then dried over anhydrous Na_2SO_4. The solvent was evaporated under reduced pressure and the crude product was purified by chromatography on silica-gel to afford 2-phenylquinazolin-4(3H)-one (**4aa**) in 43% isolated yield.

2.4 General procedure for the synthesis of 2-phenyl-2,3-dihydroquinazolin- 4(1H)-one (5aa) with Scheme 3:

o-Aminobenzanitrile (120.8 mg, 1 mmol), benzaldehyde (216.7 mg, 2 mmol), $CuCl_2$ (17.3 mg, 0.1 mmol), Cs_2CO_3 (653.7 mg, 2 mmol) and L-proline （23.3 mg, 0.2 mmol) in H_2O (2 mL) was added in a 5 ml reaction bottle under nitrogen. Remove the air inside the tube under the reduced pressure and flush with N_2, repeat this operation 3 times and protected the starting materials under N_2. The mixture was stirred at 100 ℃ for 24 hours. After cooling to room temperature, the resulting mixture was quenched with NH_4Cl solution and extracted with ethyl acetate. The combined organic layers were washed with brine and then dried over anhydrous Na_2SO_4. The solvent was evaporated under reduced pressure and the crude product was purified by chromatography on silica-gel to afford 2-phenyl-2,3-dihydroquinazolin-4(1H)-one (**5aa**) in 76% isolated yield.

2.5 General procedure for the synthesis of 2-phenyl-2,3-dihydroquinazolin- 4(1H)-one (5aa) with Scheme 4:

o-Aminobenzamide (139.1 mg, 1 mmol), benzaldehyde (209.9 mg, 2 mmol), $CuCl_2$ (17.6 mg, 0.1 mmol), Cs_2CO_3 (657.0 mg, 2 mmol) and L-proline （23.6 mg, 0.2 mmol) in H_2O (2 mL) was added in a 5 ml reaction bottle. The the mixture was stirred at 100 ℃ for 14 hours. After cooling to room temperature, the resulting mixture was quenched with NH_4Cl solution and extracted with ethyl acetate. The combined organic layers were washed with brine and then dried over anhydrous Na_2SO_4. The solvent was evaporated under reduced pressure and the crude product was purified by chromatography on silica-gel to afford 2-phenyl-2,3-dihydroquinazolin-4(1H)-one (**5aa**) in 95% isolated yield.

3. Spectral and Analytical Data of products

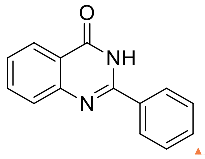

2-phenylquinazolin-4(3H)-one (4aa)[1]:

^{1}HNMR (400 MHz, $CDCl_3$-*d*) *δ* 11.24 (s, 1H,-NH-), 8.27 (d, *J* = 7.8 Hz, 1H, Ar-H), 8.16 (dd, *J* = 6.6, 3.0 Hz, 2H, Ar-H), 7.82 – 7.71 (m, 2H, Ar-H), 7.57 – 7.49 (m, 3H, Ar-H), 7.48 – 7.41 (m, 1H, Ar-H). ^{13}C NMR (100 MHz, $CDCl_3$-*d*) *δ* 151.60, 134.87, 132.77, 131.64, 129.05, 127.97, 127.25, 126.79, 126.34, 120.84. HRMS (ESI) calcd for $C_{14}H_{11}N_2O$ $[M+H]^+$: 223.0871. Found: 223.0866.

Formatted: Font: Not Bold, Italic
Formatted: Font: 10.5 pt
Formatted: Font: (Default) Times New Roman, 10.5 pt
Formatted: Font: (Default) Times New Roman, 10.5 pt
Formatted: Font: (Default) Times New Roman, 10.5 pt
Formatted: Font: (Default) Times New Roman, 10.5 pt
Formatted: Font: (Asian) +Headings Asian (宋体)
Formatted: Font: (Default) Times New Roman
Formatted: Font: Not Bold, Italic
Formatted: Font: Italic
Formatted: Font: Italic
Formatted: Font: Not Bold, Italic

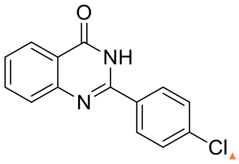

Formatted: Font: (Default) Times New Roman

2-(4-chlorophenyl)quinazolin-4(3H)-one (4ab)[1]:

^{1}H NMR (400 MHz, DMSO-d_6) δ 12.60 (s, 1H, -NH-), 8.21 (d, J = 8.6 Hz, 2H, Ar-H), 8.16 (d, J = 7.8 Hz, 1H, Ar-H), 7.85 (t, J = 7.6 Hz, 1H, Ar-H), 7.75 (d, J = 8.1 Hz, 1H, Ar-H), 7.63 (d, J = 8.6 Hz, 2H, Ar-H), 7.54 (t, J = 7.4 Hz, 1H, Ar-H).

^{13}C NMR (101 MHz, DMSO) δ 162.56, 151.74, 148.98, 136.70, 135.08, 131.97, 130.03, 129.10, 127.93, 127.19, 126.28, 121.42.

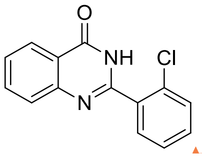

Formatted: Font: (Default) Times New Roman

2-(2-chlorophenyl)quinazolin-4(3H)-one (4ac)[1]:

^{1}H NMR (400 MHz, $CDCl_3$-d) δ 10.62 (s, 1H, -NH-), 8.27 (d, J = 7.9 Hz, 1H, Ar-H), 7.81 (d, J = 4.0 Hz, 3H, Ar-H), 7.58 – 7.40 (m, 4H, Ar-H).

^{13}C NMR (101 MHz, $CDCl_3$) δ 162.42, 151.12, 148.92, 134.83, 132.72, 132.03, 131.91, 131.34, 130.51, 127.91, 127.38, 127.30, 126.44, 121.02.

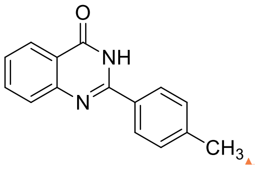

Formatted: Font: (Default) Times New Roman

2-(*p*-tolyl)quinazolin-4(3H)-one (4ad)[1]:

Formatted: Font: Italic

^{1}H NMR (400 MHz, $CDCl_3$-d) δ 11.28 (s, 1H, -NH-), 8.36 (d, J = 7.9 Hz, 1H, Ar-H), 8.15 (d, J = 8.2 Hz, 2H, Ar-H), 7.83 (q, J = 8.4 Hz, 2H, Ar-H), 7.52 (t, J = 7.3 Hz, 1H, Ar-H), 7.41 (d, J = 8.0 Hz, 2H, Ar-H), 2.49 (s, 3H , -CH_3).

^{13}C NMR (101 MHz, $CDCl_3$) δ 163.90, 151.80, 149.52, 142.15, 134.78, 129.87, 129.69, 127.81, 127.33, 126.50, 126.31, 120.72, 21.50.

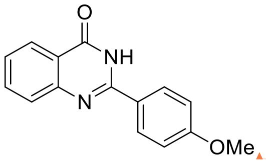

Formatted: Font: (Default) Times New Roman

2-(4-methoxyphenyl)quinazolin-4(3H)-one (4ae)[1]:

^{1}H NMR (400 MHz, DMSO-d_6) δ 12.40 (s, 1H, -NH-), 8.17 (dd, J = 25.4, 8.3 Hz, 3H, Ar-H), 7.82 (t, J = 7.5 Hz, 1H, Ar-H), 7.71 (d, J = 8.1 Hz, 1H, Ar-H), 7.49 (t, J = 7.5 Hz, 1H, Ar-H), 7.09 (d, J = 8.8 Hz, 2H, Ar-H), 3.86 (s, 3H, -CH_3).

^{13}C NMR (101 MHz, DMSO-d_6) δ 162.70, 162.27, 152.26, 149.34, 134.94, 129.86, 127.69, 126.52, 126.23, 125.21, 121.10, 114.40, 55.86.

Formatted: Font: Italic

Formatted: Subscript

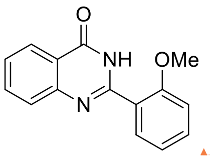

Formatted: Font: (Default) Times New Roman

2-(2-methoxyphenyl)quinazolin-4(3H)-one (4af)[1]:

^{1}H NMR (400 MHz, CDCl$_3$-*d*) *δ* 10.92 (s, 1H, -NH-), 8.53 (d, *J* = 9.4 Hz, 1H, Ar-H), 8.30 (d, *J* = 7.8 Hz, 1H, Ar-H), 7.77 (q, *J* = 8.3 Hz, 2H, Ar-H), 7.55 – 7.43 (m, 2H, Ar-H), 7.16 (t, *J* = 7.6 Hz, 1H, Ar-H), 7.06 (d, *J* = 8.4 Hz, 1H, Ar-H), 4.05 (s, 3H, -CH$_3$).

^{13}C NMR (101 MHz, CDCl$_3$) *δ* 161.82, 157.72, 150.71, 149.32, 134.40, 133.13, 131.47, 127.78, 126.39, 126.34, 121.79, 121.12, 119.84, 111.77, 56.09.

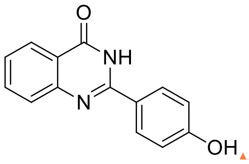

Formatted: Font: (Default) Times New Roman

2-(4-hydroxyphenyl)quinazolin-4(3H)-one (4ag)[2]:

^{1}H NMR (400 MHz, DMSO-*d*$_6$) δ 12.30 (s, 1H, -NH-), 10.15 (s, 1H, -OH), 8.11 (dd, *J* = 12.1, 8.6 Hz, 3H, Ar-H), 7.80 (t, *J* = 7.5 Hz, 1H, Ar-H), 7.68 (d, *J* = 8.1 Hz, 1H, Ar-H), 7.47 (t, *J* = 7.4 Hz, 1H, Ar-H), 6.90 (d, *J* = 8.5 Hz, 2H, Ar-H).

^{13}C NMR (101 MHz, DMSO) *δ* 162.71, 160.96, 152.53, 149.45, 134.88, 129.98, 127.60, 126.31, 126.22, 123.62, 120.99, 115.76.

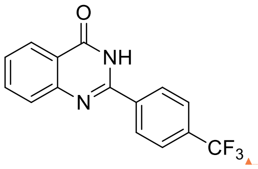

Formatted: Font: (Default) Times New Roman

2-(4-(trifluoromethyl)phenyl)quinazolin-4(3H)-one (4ai)[3]:

^{1}H NMR (400 MHz, DMSO-*d*$_6$) *δ* 12.74 (s, 1H, -NH-), 8.38 (d, *J* = 8.1 Hz, 2H, Ar-H), 8.19 (d, *J* = 7.9 Hz, 1H, Ar-H), 7.93 (d, *J* = 8.2 Hz, 2H, Ar-H), 7.87 (t, *J* = 7.6 Hz, 1H, Ar-H), 7.78 (d, *J* = 8.1 Hz, 1H, Ar-H), 7.57 (t, *J* = 7.5 Hz, 1H, Ar-H).

^{13}C NMR (101 MHz, DMSO-*d*$_6$) δ 162.52 , 151.58 , 148.84 , 137.02 , 135.14 , 131.51 (d, $^{1}J_{C,F}$ = 31.7 Hz), 129.14, 128.09, 127.52, 126.31, 125.90 (dd, $^{3}J_{C,F}$ = 3.7, $^{2}J_{C,F}$ = 13.1Hz), 123.01 , 121.61.

^{19}F NMR (376 MHz, DMSO) *δ* -61.35.

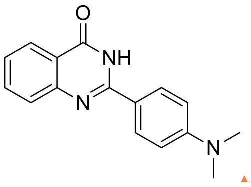

Formatted: Font: (Default) Times New Roman

2-(4-(dimethylamino)phenyl)quinazolin-4(3H)-one (4aj)[4]:

^{1}H NMR (400 MHz, CDCl$_3$-*d*) *δ* 10.70 (s, 1H, -NH-), 8.32 (d, *J* = 7.7 Hz, 1H, Ar-H), 8.11 (d, *J* = 9.0 Hz, 2H, Ar-H), 7.78 (s, 2H, Ar-H), 7.44 (s, 1H, Ar-H), 6.82 (d, *J* = 9.0 Hz, 2H, Ar-H), 3.10 (s, 6H, -CH$_3$).

^{13}C NMR (101 MHz, CDCl$_3$) *δ* 163.47, 152.54, 151.79, 149.82, 134.62, 128.45, 127.31, 126.31, 125.63,

120.30, 119.00, 111.64, 40.08.

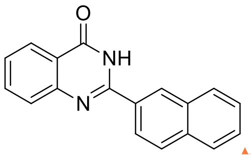

Formatted: Font: (Default) Times New Roman

2-(naphthalen-2-yl)quinazolin-4(3H)-one (4ak)[2]:

^{1}H NMR (400 MHz, DMSO-d_6) δ 12.67 (s, 1H, -NH-), 8.83 (s, 1H, Ar-H), 8.36 – 8.26 (m, 1H, Ar-H), 8.24 – 8.16 (m, 1H, Ar-H), 8.05 (d, J = 16.5 Hz, 3H, Ar-H), 7.84 (d, J = 21.9 Hz, 2H, Ar-H), 7.60 (d, J = 38.2 Hz, 3H, Ar-H).

^{13}C NMR (101 MHz, DMSO) δ 162.65, 152.64, 149.19, 135.07, 134.54, 132.69, 130.37, 129.37, 128.58, 128.52, 128.34, 128.08, 127.97, 127.33, 127.08, 126.32, 124.91, 121.46.

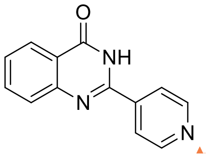

Formatted: Font: (Default) Times New Roman

2-(pyridin-4-yl)quinazolin-4(3H)-one (4al)[3]:

^{1}H NMR (400 MHz, –CDCl$_3$-d) δ 12.03 (s, 1H, -NH-), 8.93 (d, J = 5.5 Hz, 2H, Ar-H), 8.40 (d, J = 7.9 Hz, 1H, Ar-H), 8.23 (d, J = 5.8 Hz, 2H, Ar-H), 7.90 (d, J = 6.1 Hz, 2H, Ar-H), 7.63 (ddd, J = 8.1, 6.0, 2.2 Hz, 1H, Ar-H).

^{13}C NMR (101 MHz, DMSO) δ 162.43, 150.94, 150.69, 148.68, 140.33, 135.19, 128.21, 127.81, 126.34, 121.99, 121.90.

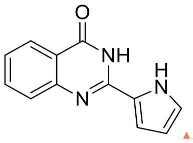

Formatted: Font: (Default) Times New Roman

2-(1H-pyrrol-2-yl)quinazolin-4(3H)-one (4ap)[5][5]:

^{1}H NMR (400 MHz, DMSO-d_6) δ 12.20 (s, 1H, -NH-), 11.74 (s, 1H, -NH-), 8.09 (d, J = 7.7 Hz, 1H, Ar-H), 7.77 (t, J = 7.4 Hz, 1H, Ar-H), 7.62 (d, J = 8.1 Hz, 1H, Ar-H), 7.41 (t, J = 7.4 Hz, 1H, Ar-H), 7.31 (s, 1H, Ar-H), 7.04 (s, 1H, Ar-H), 6.22 (s, 1H, Ar-H).

^{13}C NMR (101 MHz, DMSO) δ 162.30, 149.71, 146.80, 134.91, 126.85, 126.37, 125.62, 124.69, 124.27, 120.90, 112.89, 110.15.

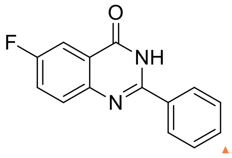

Formatted: Font: (Default) Times New Roman

6-~~fluoro~~Fluoro-2-phenylquinazolin-4(3H)-one (4ea)[6]:

Commented [微软中国2]: 注意首字母大写的问题

^{1}H NMR (400 MHz, DMSO-d_6) δ 12.66 (s, 1H, -NH-), 8.18 (d, J = 6.9 Hz, 2H, Ar-H), 7.87 – 7.80 (m, 2H, Ar-H), 7.73 (td, J = 8.7, 3.0 Hz, 1H, Ar-H), 7.63 – 7.52 (m, 3H, Ar-H).

Formatted: Font color: Text 1

^{13}C NMR (101 MHz, DMSO-d_6) δ 162.06, 160.40 (d, $^{1}J_{C,F}$ ~~J~~= 246.5Hz), 152.27, 146.04 , 132.97, 131.83, 130.74 (d, $^{3}J_{C,F}$ = 8.4 Hz), 129.02, 128.16, 123.47 (d, $^{2}J_{C,F}$ = 24.3 Hz), 122.61 (d, $^{3}J_{C,F}$ = 9.5

Hz), 110.92 (d, $^{2}J_{C,F}$ ~~J~~ = 23.2 Hz). ^{19}F NMR (376 MHz, DMSO) δ -113.49, -113.50, -113.51, -113.53, -113.53, -113.55.

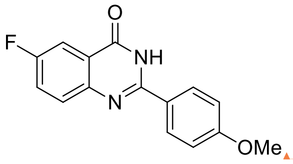

6-fluoro-2-(4-methoxyphenyl)quinazolin-4(3H)-one (4ee)[7]:

^{1}H NMR (400 MHz, $CDCl_3$-*d*) *δ* 9.87 (s, 1H, -NH-), 8.05 (d, *J* = 8.3 Hz, 2H, Ar-H), 7.95 (d, *J* = 7.5 Hz, 1H, Ar-H), 7.82 (dd, *J* = 8.9, 4.6 Hz, 1H, Ar-H), 7.54 (d, *J* = 6.8 Hz, 1H, Ar-H), 7.13 – 7.06 (m, 2H, Ar-H), 3.94 (s, 3H, $-CH_3$). ^{13}C NMR (101 MHz, DMSO-d_6) δ 162.30 , 162.13 (d, $^{4}J_{C,F}$ = 3.0 Hz), 160.14 (d, $^{1}J_{C,F}$ = 245.0 Hz), 151.84 , 146.25 , 130.48 (d, $^{3}J_{C,F}$ = 8.2 Hz), 129.85 , 125.05 , 123.42 (d, $^{2}J_{C,F}$ = 24.1 Hz), 122.24 (d, $^{3}J_{C,F}$ = 8.4 Hz), 114.43 , 110.85 (d, $^{2}J_{C,F}$ = 23.2 Hz), 55.88 . ^{19}F NMR (376 MHz, DMSO) *δ* -114.17. HRMS (ESI) calcd for $C_{15}H_{12}FN_2O_2$[M+H]$^{+}$: 271.0877. Found: 271.0876.

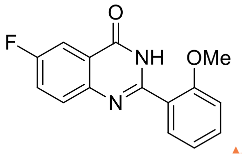

6-fluoro-2-(2-methoxyphenyl)quinazolin-4(3H)-one (4ef)[7]:

^{1}HNMR(400MHz, $CDCl_3$-*d*) *δ* 11.00 (s, 1H, -NH-), 8.53 (d, J = 7.9 Hz, 1H, Ar-H), 7.94 (d, J = 8.3 Hz, 1H, Ar-H), 7.80 (dd, J = 8.9, 4.9 Hz, 1H, Ar-H), 7.57 – 7.46 (m, 2H, Ar-H), 7.18 (t, J = 7.6 Hz, 1H, Ar-H), 7.09 (d, J = 8.4 Hz, 1H, Ar-H), 4.08 (s, 3H, $-CH_3$). ^{13}C NMR (101 MHz, DMSO-d_6) δ 161.07 (d, $^{4}J_{C,F}$ = 3.4 Hz), 160.40(d, $^{1}J_{C,F}$ = 245.43 Hz), 157.52 , 152.27 , 146.33 , 132.66 , 130.85 , 130.65 (d, $^{3}J_{C,F}$ = 8.5 Hz), 123.32 (d, $^{2}J_{C,F}$ = 23.9 Hz), 122.95 , 122.62 (d, $^{3}J_{C,F}$ = 8.5 Hz), 120.82 , 112.26, 10.83 (d, $^{2}J_{C,F}$ = 23.4 Hz), 56.17. ^{19}F NMR (376 MHz, DMSO) *δ* -113.56.

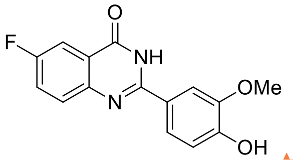

6-fluoro-2-(4-hydroxy-3-methoxyphenyl)quinazolin-4(3*H*)-one (4eq):

^{1}H NMR (400 MHz, DMSO-d_6) *δ* 12.47 (s, 1H, -NH-), 9.76 (s, 1H,-OH), 8.09 – 7.61 (m, 5H, Ar-H), 6.91 (d, *J* = 8.3 Hz, 1H, Ar-H), 3.90 (s, 3H, $-CH_3$). ^{13}C NMR (101 MHz, DMSO-d_6) δ 162.23 , 160.02 (d, $^{1}J_{C,F}$ = 244.7 Hz), 152.02 , 150.38 , 147.89 , 146.33 , 130.38 , 123.62 , 123.38 (d, $^{2}J_{C,F}$ = 24.2 Hz), 122.07 (d, $^{3}J_{C,F}$ = 7.9 Hz), 121.87 , 115.83 , 111.73 , 110.83 (d, $^{2}J_{C,F}$ = 23.2 Hz), 56.19. HRMS (ESI) calcd for $C_{15}H_{12}FN_2O_3$[M+H]$^{+}$: 287.0826. Found: 287.0825. ^{19}F NMR (376 MHz, DMSO) *δ* -114.53.

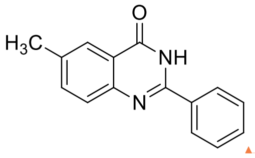

6-methyl-2-phenylquinazolin-4(3H)-one (4fa)[3]:

^{1}H NMR (400 MHz, $CDCl_3$-*d*) *δ* 10.82 (s, 1H, -NH-), 8.19 (d, *J* = 5.7 Hz, 2H, Ar-H), 8.14 (s, 1H, Ar-

Formatted: Font: (Default) +Body (Calibri), 10.5 pt

Formatted: Font: (Default) Times New Roman

Formatted: Font: (Default) Times New Roman

Formatted: Font: (Default) Times New Roman

Commented [微软中国3]: 注意斜体

Formatted: Font: Italic

Formatted: Font: (Default) Times New Roman

H), 7.76 (d, J = 8.3 Hz, 1H, Ar-H), 7.64 (d, J = 19.1 Hz, 4H, Ar-H), 2.55 (s, 3H, $-CH_3$). 13C NMR (101 MHz, DMSO) δ 162.56, 151.86, 147.15, 136.71, 136.28, 133.19, 131.63, 128.99, 128.04, 127.79, 125.65, 121.13, 21.26.

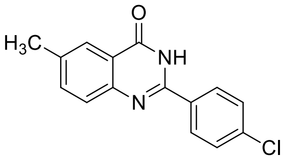

2-(4-chlorophenyl)-6-methylquinazolin-4(3H)-one (4fb)[8]:

^{1}H NMR (400 MHz, DMSO-d_6) δ 12.53 (s, 1H, -NH-), 8.20 (d, J = 8.3 Hz, 2H, Ar-H), 7.96 (s, 1H, Ar-H), 7.63 (s, 4H, Ar-H), 2.47 (s, 3H, $-CH_3$). ^{13}C NMR (101 MHz, DMSO) δ 162.49, 150.95, 147.00, 136.95, 136.53, 136.35, 132.05, 129.91, 129.08, 127.82, 125.68, 121.16, 21.27.

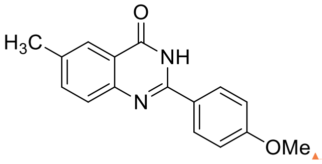

Formatted: Font: (Default) Times New Roman

2-(4-methoxyphenyl)-6-methylquinazolin-4(3H)-one (4fe)[3]:

^{1}H NMR (400 MHz, DMSO-d_6) δ 12.32 (s, 1H, -NH-), 8.18 (d, J = 8.8 Hz, 2H, Ar-H), 7.93 (s, 1H, Ar-H), 7.67 – 7.58 (m, 2H, Ar-H), 7.08 (d, J = 8.8 Hz, 2H, Ar-H), 3.85 (s, 3H, $-CH_3$), 2.45 (s, 3H, $-CH_3$). ^{13}C NMR (101 MHz, DMSO) δ 162.68, 162.14, 151.47, 147.34, 136.20, 136.16, 129.70, 127.54, 125.62, 125.31, 120.82, 114.37, 55.84, 21.22.

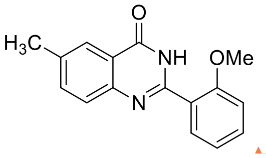

Formatted: Font: (Default) Times New Roman

2-(2-methoxyphenyl)-6-methylquinazolin-4(3H)-one (4ff):

^{1}H NMR (400 MHz, $CDCl_3$-d) δ 10.88 (s, 1H, -NH-), 8.52 (d, J = 7.6 Hz, 1H, Ar-H), 8.10 (s, 1H, Ar-H), 7.69 (d, J = 8.3 Hz, 1H, Ar-H), 7.58 (d, J = 9.8 Hz, 1H, Ar-H), 7.50 (t, J = 7.8 Hz, 1H, Ar-H), 7.16 (t, J = 7.6 Hz, 1H, Ar-H), 7.06 (d, J = 8.4 Hz, 1H, Ar-H), 4.05 (s, 3H, $-CH_3$), 2.50 (s, 3H, $-CH_3$).

^{13}C NMR (101 MHz, DMSO) δ 161.54, 157.55, 151.88, 147.46, 144.31, 136.62, 136.09, 132.52, 130.83, 127.68, 125.54, 123.08, 120.85, 112.29, 56.19, 21.25. HRMS (ESI) calcd for $C_{16}H_{15}N_2O_2[M+H]^+$:267.1128. Found: 267.1127.

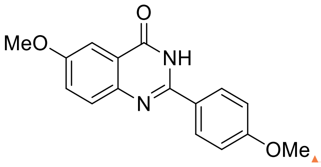

Formatted: Font: (Default) Times New Roman

6-methoxy-2-(4-methoxyphenyl)quinazolin-4(3H)-one (4ge)[9]:

^{1}H NMR (400 MHz, DMSO-d_6) δ 12.37 (s, 1H,-NH-), 8.17 (d, J = 8.8 Hz, 2H, Ar-H), 7.66 (d, J = 8.9 Hz, 1H, Ar-H), 7.53 (d, J = 2.8 Hz, 1H, Ar-H), 7.42 (d, J = 11.8 Hz, 1H, Ar-H), 7.08 (d, J = 8.8 Hz, 2H, Ar-H), 3.89 (s, 3H, $-CH_3$), 3.85 (s, 3H, $-CH_3$). 13C NMR (101 MHz, DMSO) δ 162.51, 161.98, 157.83,

150.14, 143.82, 129.53, 129.41, 125.35, 124.51, 121.84, 114.38, 106.22, 56.02, 55.84.

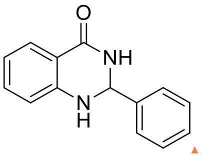

Formatted: Font: (Default) Times New Roman

2-phenyl-2,3-dihydroquinazolin-4(1H)-one (5aa)[10]:

^{1}H NMR (400 MHz, $CDCl_3$-*d*) *δ* 7.97 (d, *J* = 7.8 Hz, 1H, Ar-H), 7.67 – 7.56 (m, 2H, Ar-H), 7.55 – 7.41 (m, 3H, , Ar-H), 7.36 (t, *J* = 7.7 Hz, 1H, Ar-H), 6.93 (t, *J* = 7.5 Hz, 1H, Ar-H), 6.70 (d, *J* = 8.0 Hz, 1H, Ar-H), 5.93 (s, 1H,-CH-), 5.80 (s, 1H,-NH-), 4.42 (s, 1H, -NH-). ^{13}C NMR (101 MHz, DMSO) *δ* 163.98, 148.27, 142.04, 133.70, 128.85, 128.72, 127.75, 127.26, 117.51, 115.36, 114.80, 66.96. HRMS (ESI) calcd for $C_{14}H_{13}N_2O[M+H]^+$: 225.1022. Found: 225.1021.

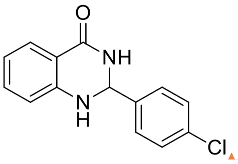

Formatted: Font: (Default) Times New Roman

2-(4-chlorophenyl)-2,3-dihydroquinazolin-4(1H)-one (5ab)[11]:

^{1}H NMR (400 MHz, DMSO-d_6) δ 8.33 (s, 1H,-NH-), 7.62 (dd, *J* = 7.7, 1.5 Hz, 1H, Ar-H), 7.52 (d, *J* = 8.6 Hz, 2H, Ar-H), 7.49 – 7.43 (m, 2H, Ar-H), 7.30 – 7.21 (m, 1H, Ar-H), 7.14 (s, 1H,-CH-), 6.75 (d, *J* = 7.7 Hz, 1H, Ar-H), 6.69 (t, *J* = 7.9 Hz, 1H, Ar-H), 5.78 (s, 1H, -NH-). ^{13}C NMR (101 MHz, DMSO) *δ* 163.90, 148.06, 141.08, 133.80, 133.39, 129.16, 128.72, 127.78, 117.70, 115.36, 114.87, 66.18.

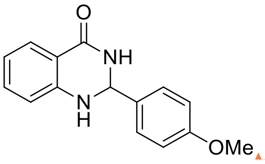

Formatted: Font: (Default) Times New Roman

2-(4-methoxyphenyl)-2,3-dihydroquinazolin-4(1H)-one (5ae)[10]:

^{1}H NMR (400 MHz, DMSO-d_6) *δ* 8.18 (s, 1H,-NH-), 7.62 (d, *J* = 9.1 Hz, 1H, Ar-H), 7.42 (d, *J* = 8.7 Hz, 2H, Ar-H), 7.28 – 7.20 (m, 1H, Ar-H), 7.01 (s, 1H,-NH-), 6.95 (d, *J* = 8.7 Hz, 2H, Ar-H), 6.75 (d, *J* = 7.8 Hz, 1H, Ar-H), 6.68 (t, *J* = 7.9 Hz, 1H, Ar-H), 5.71 (s, 1H, -CH-), 3.75 (s, 3H $-CH_3$). ^{13}C NMR (101 MHz, DMSO) *δ* 164.10, 159.84, 148.42, 133.88, 133.64, 128.62, 127.75, 117.49, 115.41, 114.82, 114.04, 66.72, 55.58.

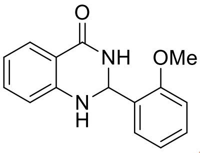

Formatted: Font: (Default) Times New Roman

2-(2-methoxyphenyl)-2,3-dihydroquinazolin-4(1H)-one (5af)~~[13]~~[7]:

Formatted: Superscript

Formatted: Superscript

^{1}H NMR (400 MHz, $CDCl_3$-*d*) *δ* 7.91 (d, *J* = 7.8 Hz, 1H, Ar-H), 7.56 (d, *J* = 7.6 Hz, 1H, Ar-H), 7.32 (dt, *J* = 15.4, 7.6 Hz, 2H, Ar-H), 7.02 – 6.91 (m, 2H, Ar-H), 6.84 (t, *J* = 7.5 Hz, 1H, Ar-H), 6.67 (d, *J* = 8.1 Hz, 1H, Ar-H), 6.28 (s, 1H,-CH-), 6.25 (s, 1H, -NH-), 4.76 (s, 1H, -NH-), 3.90 (s, 3H, $-CH_3$). ^{13}C NMR (101 MHz, $CDCl_3$) *δ* 164.99, 156.35, 147.07, 133.87, 130.11, 128.46, 127.84, 126.66, 120.90, 119.01, 115.21, 114.57, 110.58, 62.43, 55.43.

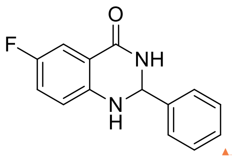

Formatted: Font: (Default) Times New Roman

6-fluoro-2-phenyl-2,3-dihydroquinazolin-4(1H)-one (5ea):

^{1}H NMR (400 MHz, DMSO-d_6) δ 8.48 (s, 1H,-NH-), 7.52 (dd, J = 8.0, 1.3 Hz, 2H, Ar-H), 7.46 – 7.32 (m, 4H, Ar-H), 7.16 (td, J = 8.7, 3.1 Hz, 1H, Ar-H), 7.10 (s, 1H, -NH-), 6.81 (dd, J = 8.9, 4.5 Hz, 1H, Ar-H), 5.77 (s, 1H, -CH-). ^{13}C NMR (101 MHz, DMSO-d_6) δ 163.22 , 155.14 (d, $^{1}J_{C, F}$ = 233.4 Hz), 145.04 , 141.57 , 128.97 , 128.75 , 127.37 , 121.17 (d, $^{2}J_{C, F}$ = 23.4 Hz), 116.58 (d, $^{3}J_{C, F}$ = 7.1 Hz), 116.12 (d, $^{3}J_{C, F}$ = 6.6 Hz), 112.90 (d, $^{2}J_{C, F}$ = 22.9 Hz), 67.14 . ^{19}F NMR (376 MHz, DMSO) δ -125.22.

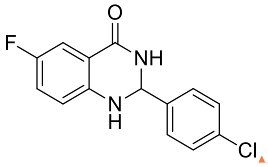

Formatted: Font: (Default) Times New Roman

2-(4-chlorophenyl)-6-fluoro-2,3-dihydroquinazolin-4(1H)-one (5eb):

^{1}H NMR (400 MHz, DMSO-d_6) δ 8.50 (s, 1H, -NH-), 7.55 – 7.49 (m, 2H, Ar-H), 7.49 – 7.43 (m, 2H, Ar-H), 7.32 (dd, J = 9.0, 3.1 Hz, 1H, Ar-H), 7.16 (td, J = 8.7, 3.1 Hz, 1H, Ar-H), 7.12 (s, 1H, -NH-), 6.79 (dd, J = 8.9, 4.5 Hz, 1H), 5.77 (s, 1H, -CH-). ^{13}C NMR (101 MHz, DMSO-d_6) δ 163.07, 155.20 (d, $^{1}J_{C, F}$ = 233.5 Hz), 144.79, 140.62, 133.50, 129.24, 128.74, 121.26 (d, $^{2}J_{C, F}$ = 23.5 Hz), 116.67 (d, $^{3}J_{C, F}$ = 7.1 Hz), 116.12 (d, $^{3}J_{C, F}$ = 6.9 Hz), 112.92 (d, $^{2}J_{C, F}$ = 22.9 Hz), 66.31. ^{19}F NMR (376 MHz, DMSO) δ -125.77.

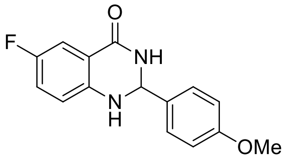

6-fluoro-2-(4-methoxyphenyl)-2,3-dihydroquinazolin-4(1H)-one (5ee):

Formatted: Font color: Auto

^{1}H NMR (400 MHz, DMSO-d_6) δ 8.36 (s, 1H, -NH-), 7.42 (d, J = 8.6 Hz, 2H, Ar-H), 7.32 (dd, J = 9.0, 3.0 Hz, 1H, Ar-H), 7.15 (td, J = 8.7, 3.1 Hz, 1H, Ar-H), 6.99 (s, 1H, Ar-H), 6.97 (s, 1H, Ar-H), 6.95 (s, 1H,-CH-), 6.78 (dd, J = 8.9, 4.5 Hz, 1H, Ar-H), 5.70 (s, 1H, -NH-), 3.76 (s, 3H, -CH_3). ^{13}C NMR (101 MHz, DMSO-d_6) δ 163.31, 159.93, 155.14 (d, $^{1}J_{C, F}$ = 233.3 Hz), 145.18, 133.39, 128.71, 121.08 (d, $^{2}J_{C, F}$ = 23.4 Hz), 116.57 (d, $^{3}J_{C, F}$ = 7.1 Hz), 116.17 (d, $^{3}J_{C, F}$ = 6.8 Hz), 114.06, 112.88 (d, $^{2}J_{C, F}$ = 23.1 Hz), 66.89, 55.57 . HRMS (ESI) calcd for $C_{15}H_{14}FN_2O_2$[M+H]$^{+}$: 273.1034 Found: 273.1032. ^{19}F NMR (376 MHz, DMSO) δ -125.76.

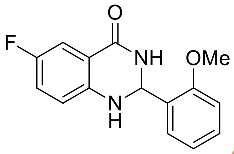

Formatted: Font: (Default) Times New Roman

6-fluoro-2-(2-methoxyphenyl)-2,3-dihydroquinazolin-4(1H)-one (5eq):

^{1}H NMR (400 MHz, DMSO-d_6) δ 8.19 (s, 1H, -NH-), 7.42 (d, *J* = 7.5 Hz, 1H, Ar-H), 7.38 – 7.30 (m, 2H, Ar-H), 7.18 – 7.09 (m, 1H, Ar-H), 7.06 (d, *J* = 8.2 Hz, 1H, Ar-H), 6.96 (t, *J* = 7.5 Hz, 1H, Ar-H), 6.81 (dd, *J* = 8.9, 4.5 Hz, 1H, Ar-H), 6.78 (s, 1H,-NH-), 6.03 (s, 1H,-CH-), 3.84 (s, 3H, -CH_3). ^{13}C NMR (101 MHz, DMSO-d_6) δ 163.44 , 156.82 , 155.09 (d, $^{1}J_{C,F}$ = 233.3 Hz), 145.14 , 130.14 , 128.93 , 127.37 , 121.08 (d, $^{2}J_{C,F}$ = 23.3 Hz), 120.55 , 116.65 (d, $^{3}J_{C,F}$ = 7.1 Hz), 115.85 (d, $^{3}J_{C,F}$ = 6.7 Hz), 112.84 (d, $^{2}J_{C,F}$ = 22.9 Hz), 111.51, 61.54, 55.93. HRMS (ESI) calcd for $C_{15}H_{14}FN_2O_2$[M+H]$^+$: 273.1034. Found: 273.1032. ^{19}F NMR (376 MHz, DMSO) *δ* -126.07.

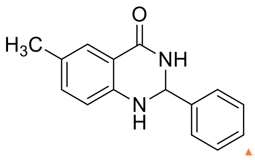

Formatted: Font: (Default) Times New Roman

6-methyl-2-phenyl-2,3-dihydroquinazolin-4(1H)-one (5fa)[10]:

Formatted: Font: (Default) Times New Roman, Not Bold

^{1}H NMR (400 MHz, $CDCl_3$-*d*) *δ* 7.77 (s, 1H, Ar-H), 7.60 (dd, *J* = 6.5, 2.7 Hz, 2H) , Ar-H, 7.49 – 7.42 (m, 3H, Ar-H), 7.16 (d, *J* = 9.3 Hz, 1H, Ar-H), 6.63 – 6.56 (m, 1H, Ar-H), 5.86 (s, 1H, -CH-), 5.73 (s, 1H, -NH-), 4.26 (s, 1H,-NH-), 2.30 (s, 3H, -CH_3). ^{13}C NMR (101 MHz, DMSO) *δ* 163.43, 156.53, 153.92, 145.12, 130.13, 128.94, 127.36, 121.07, 120.55, 116.64, 115.84, 112.82, 111.53, 61.53, 55.95.

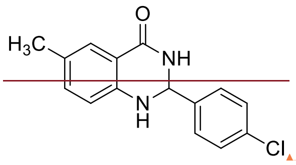

Formatted: Font: (Default) Times New Roman

~~**2-(4-chlorophenyl)-6-methyl-2,3-dihydroquinazolin-4(1H)-one (5fb)[713]:**~~

Formatted: Font: Not Bold, Superscript

Field Code Changed

~~^{1}H NMR (400 MHz, DMSO-d_6) δ 8.28 (s, 1H, Ar-H), 7.55 – 7.40 (m, 5H, Ar-H), 7.08 (d, *J* = 8.2 Hz, 1H, Ar-H), 6.95 (s, 1H, CH-), 6.67 (d, *J* = 8.2 Hz, 1H, NH-), 5.72 (s, 1H, NH-), 2.18 (s, 3H, -CH_3).~~

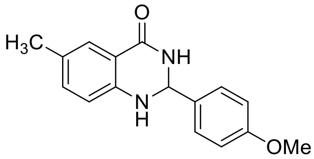

2-(4-methoxyphenyl)-6-methyl-2,3-dihydroquinazolin-4(1H)-one (5fe):

Formatted: Font color: Red

^{1}H NMR (400 MHz, DMSO-d_6) *δ* 8.11 (s, 1H, Ar-H), 7.46 – 7.31 (m, 3H, Ar-H), 7.06 (dd, *J* = 8.2, 1.9 Hz, 1H, Ar-H), 6.93 (d, *J* = 8.7 Hz, 2H, Ar-H), 6.80 (s, 1H,-CH-), 6.66 (d, *J* = 8.2 Hz, 1H,-NH-), 5.65 (s, 1H,-NH-), 3.74 (s, 3H, -CH_3), 2.18 (s, 3H, -CH_3). ^{13}C NMR (101 MHz, DMSO) *δ* 164.23, 159.80, 146.28, 134.45, 133.92, 128.63, 127.60, 126.15, 115.47, 114.97, 114.00, 66.84, 55.57, 20.50. HRMS (ESI) calcd for $C_{16}H_{17}N_2O_2$[M+H]$^+$: 269.1285. Found: 269.1283.

4. NMR Spectrums for comounds 1-32 (Figure S1- ~~S33~~S72)

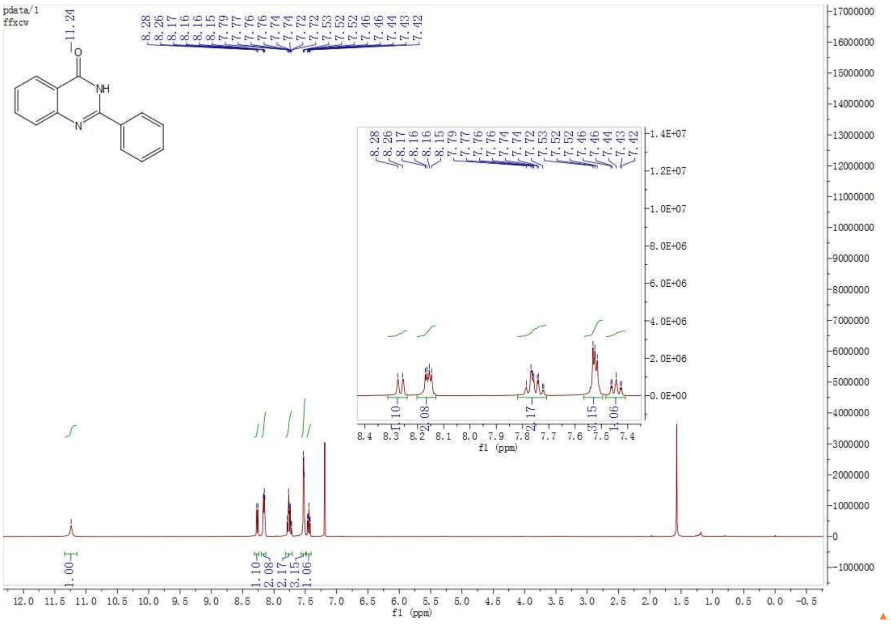

Figure S1

~~Fig. S1.~~ ^{1}H NMR for compound 1 (4aa) 2-phenylquinazolin-4(3H)-one.

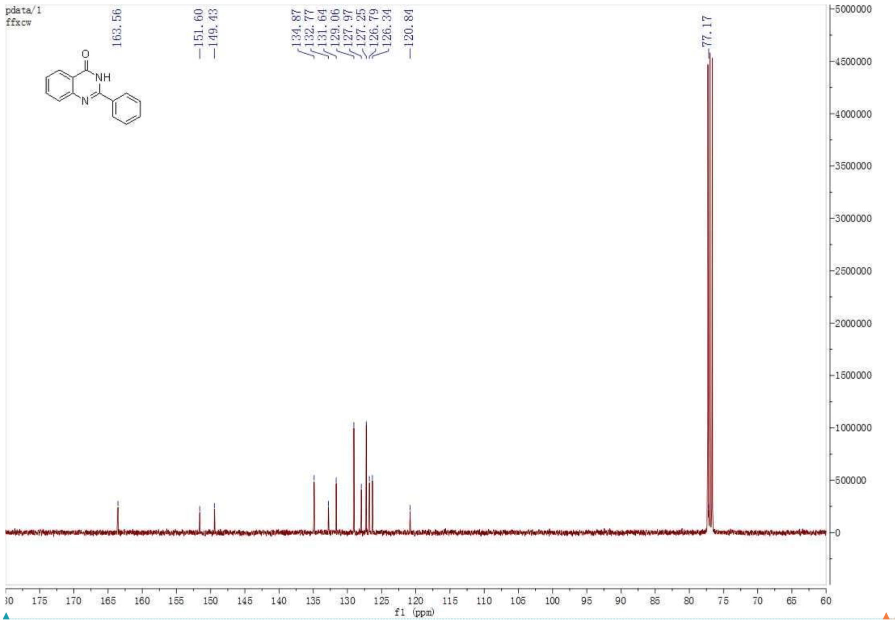

Figure S2

Formatted: Font: (Asian) +Headings Asian (宋体)

Formatted: Font: (Default) Times New Roman

Formatted: Font: (Default) Times New Roman, Not Bold

Formatted: Font: (Default) Times New Roman

Formatted: Font: (Default) Times New Roman, Not Bold

Formatted: Font: (Default) Times New Roman

Formatted: Font: (Default) Times New Roman, Not Bold

Formatted: Font: (Default) Times New Roman

~~Fig. S2.~~ ^{13}C NMR for compound 1 (4aa) 2-phenylquinazolin-4(3H)-one.

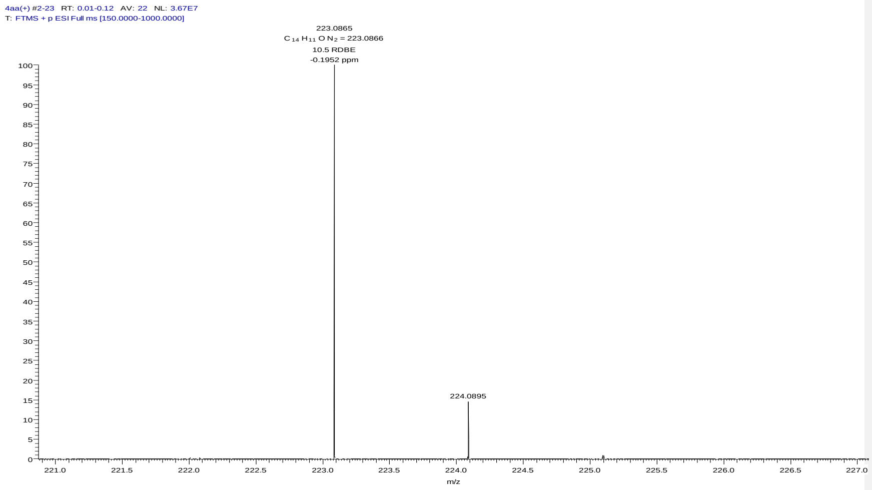

Figure S3HRMS for compound 1 (4aa) 2-phenylquinazolin-4(3H)-one.

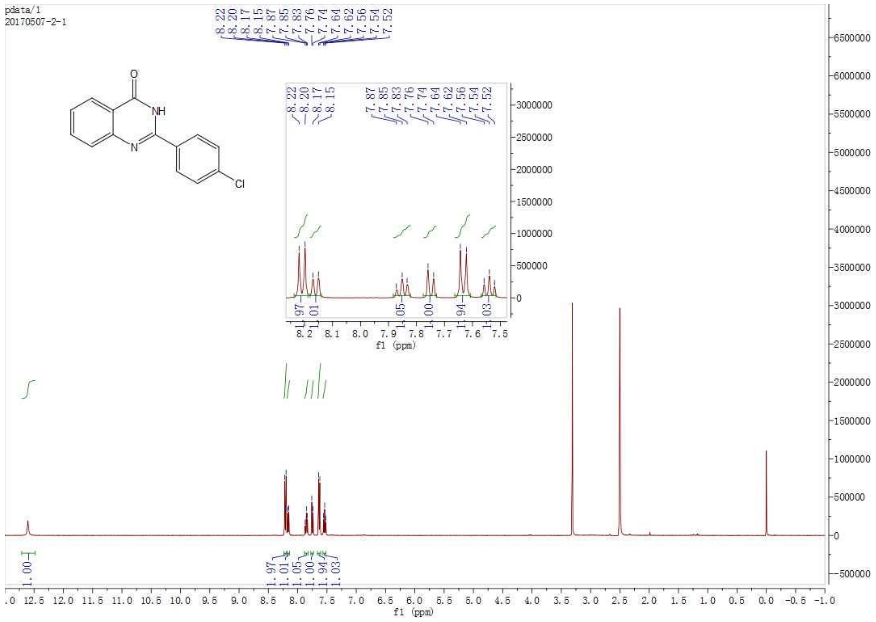

Figure S4

~~Fig. S3.~~ ^{1}H NMR for compound 2 (4ab)2-(4-chlorophenyl)quinazolin-4(3H)-one.

Formatted: Font: (Default) Times New Roman

Formatted: Font: (Default) Times New Roman

Formatted: Font: (Default) Times New Roman, Not Bold

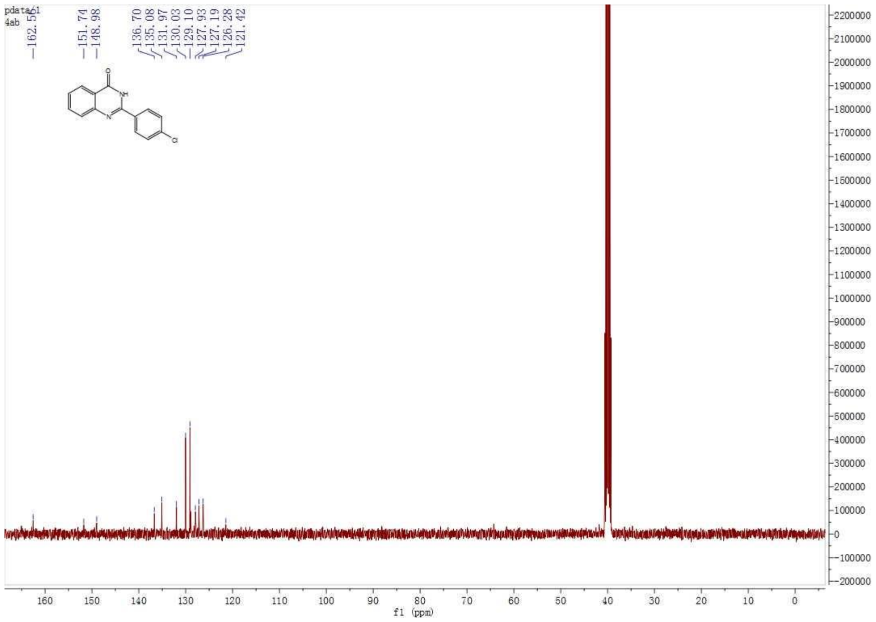

Figure S5

Fig. S3. ^{13}C NMR for compound 2 (4ab)2-(4-chlorophenyl)quinazolin-4(3H)-one.

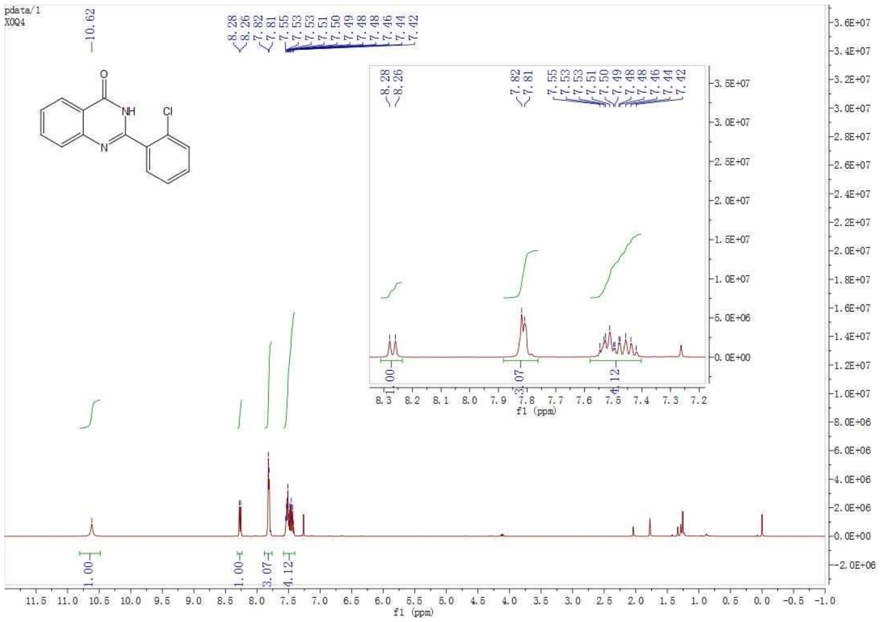

Formatted: Font: (Default) Times New Roman

Figure S6

Formatted: Font: (Default) Times New Roman, Not Bold

Fig. S4. ^{1}H NMR for compound 3 (4ac)2-(2-chlorophenyl)quinazolin-4(3H)-one.

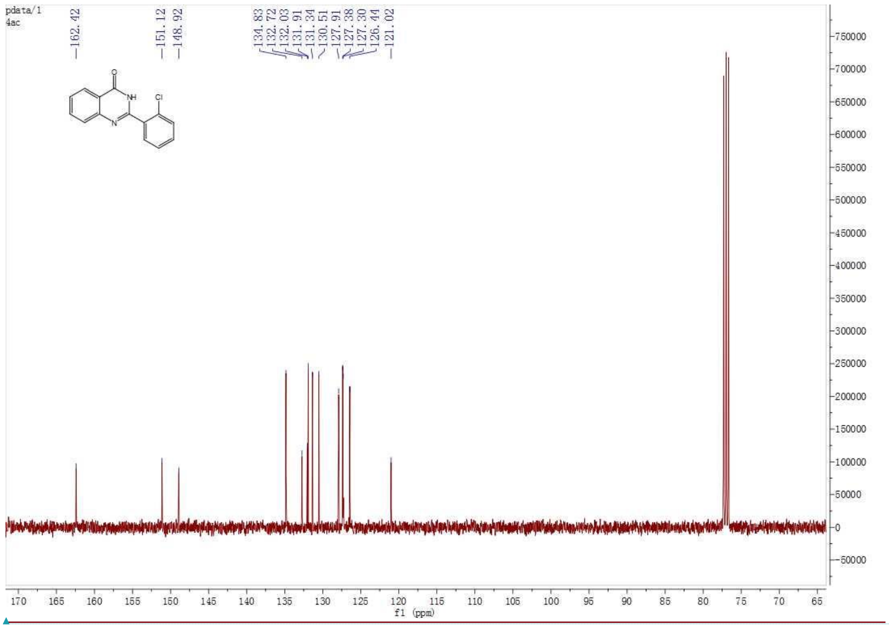

Figure S7 ^{13}C NMR for compound 3 (4ac) 2-(2-chlorophenyl)quinazolin-4(3H)-one.

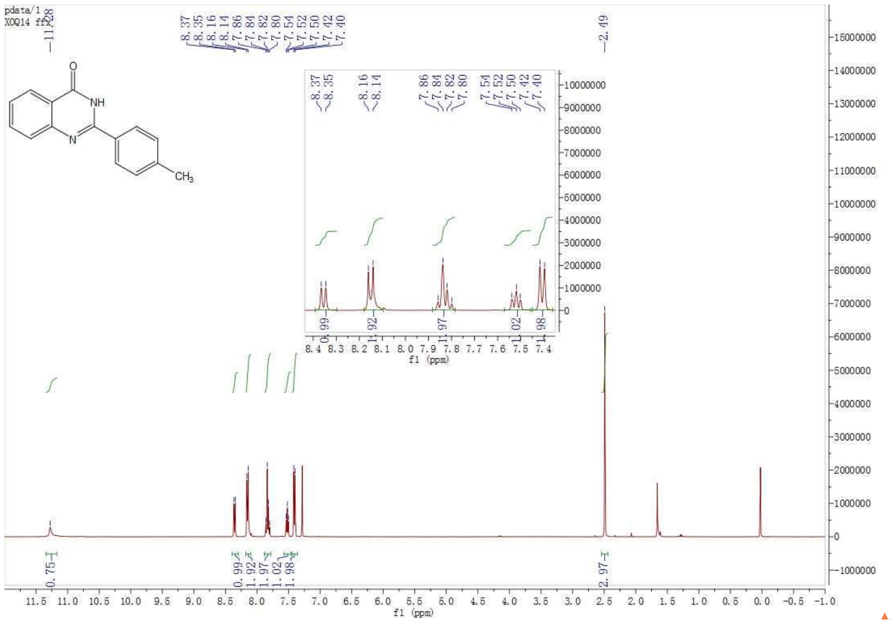

Figure S8

Fig. S5. ^{1}H NMR for compound 4 (4ad)2-(*p*-tolyl)quinazolin-4(3H)-one.

Formatted: Font: (Default) Times New Roman, Not Bold

Formatted: Font: 10 pt, Not Bold

Formatted: Font: (Default) Times New Roman

Formatted: Font: (Default) Times New Roman, Not Bold

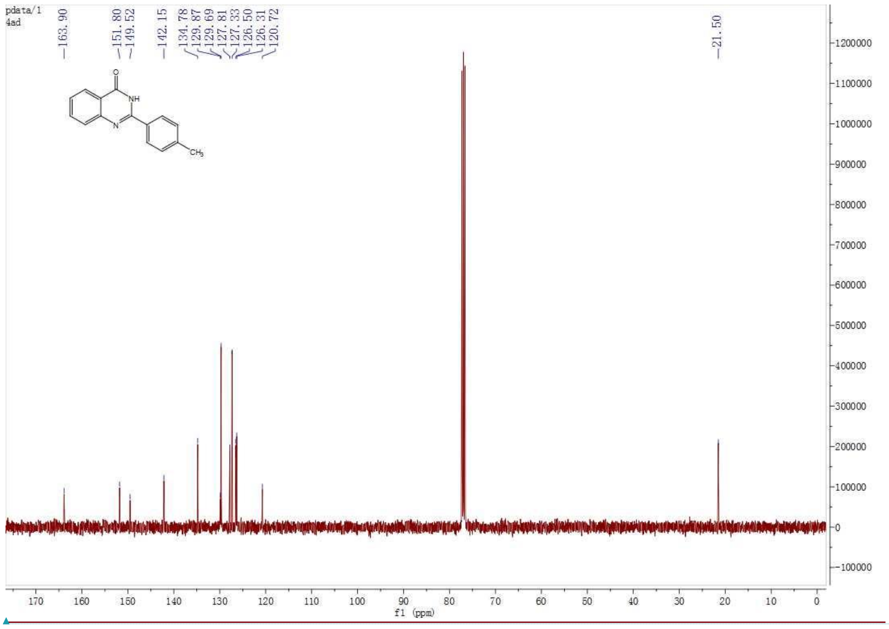

Figure S9 ^{13}C NMR for compound 4 (4ad) 2-(*p*-tolyl)quinazolin-4(3H)-one.

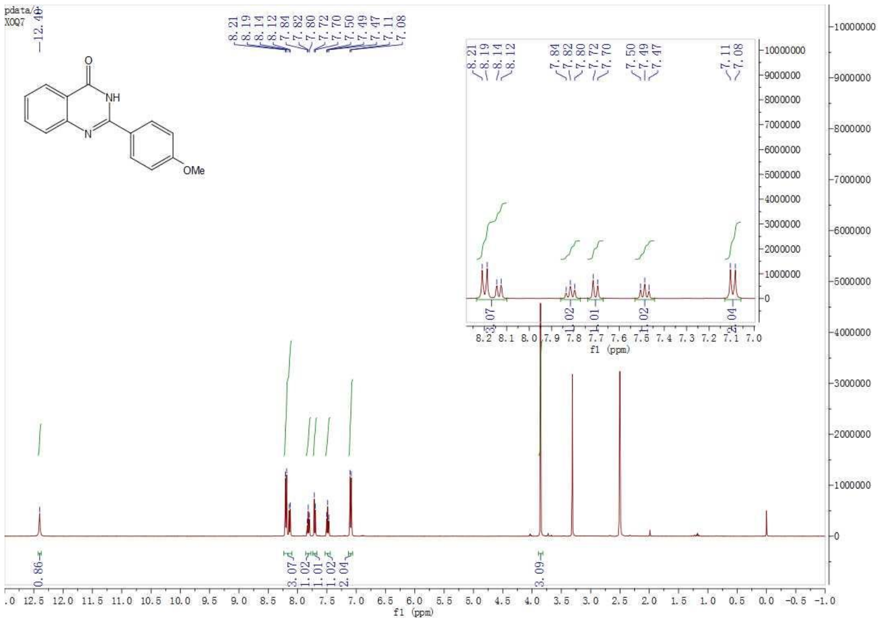

Figure S10

~~Fig. S6.~~ ^{1}H NMR for compound 5 (4ae)2-(4-methoxyphenyl)quinazolin-4(3H)-one.

Formatted: Font: (Default) Times New Roman, Not Bold

Formatted: Font: Not Bold

Formatted: Font: (Default) Times New Roman

Formatted: Font: (Default) Times New Roman, Not Bold

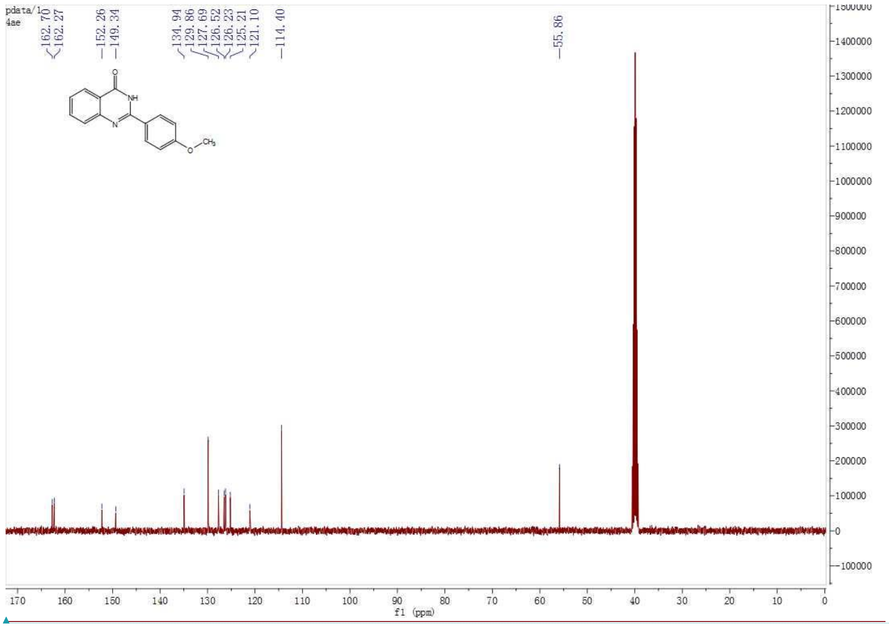

Figure S11 ^{13}CNMR for compound 5 (4ae) 2-(4-methoxyphenyl)quinazolin-4(3H)-one.

Formatted: Font: (Default) Times New Roman, 10 pt, Not Bold

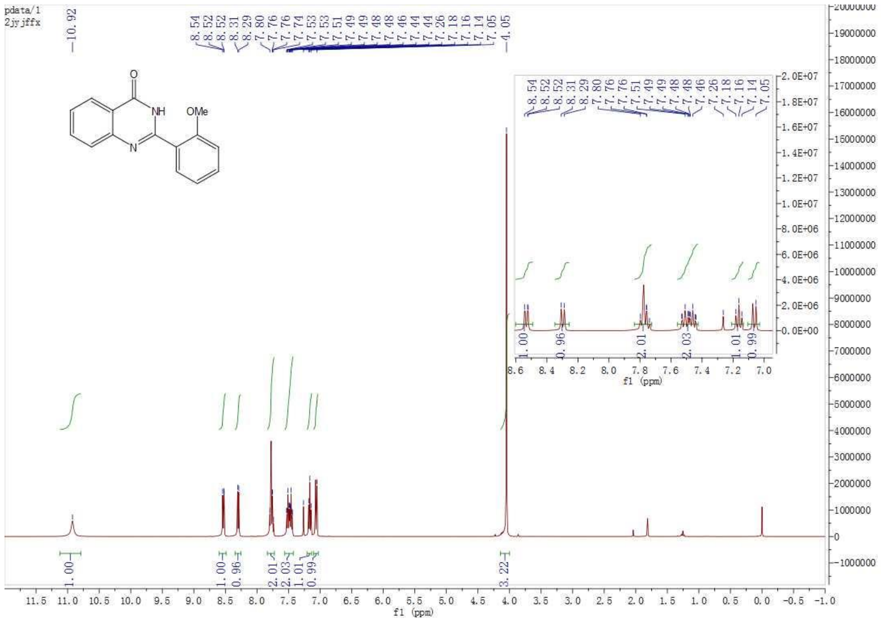

Figure S12

Fig. S7. ^{1}H NMR for compound 6 (4af)2-(2-methoxyphenyl)quinazolin-4(3H)-one.

Formatted: Font: (Default) Times New Roman

Formatted: Font: (Default) Times New Roman, Not Bold

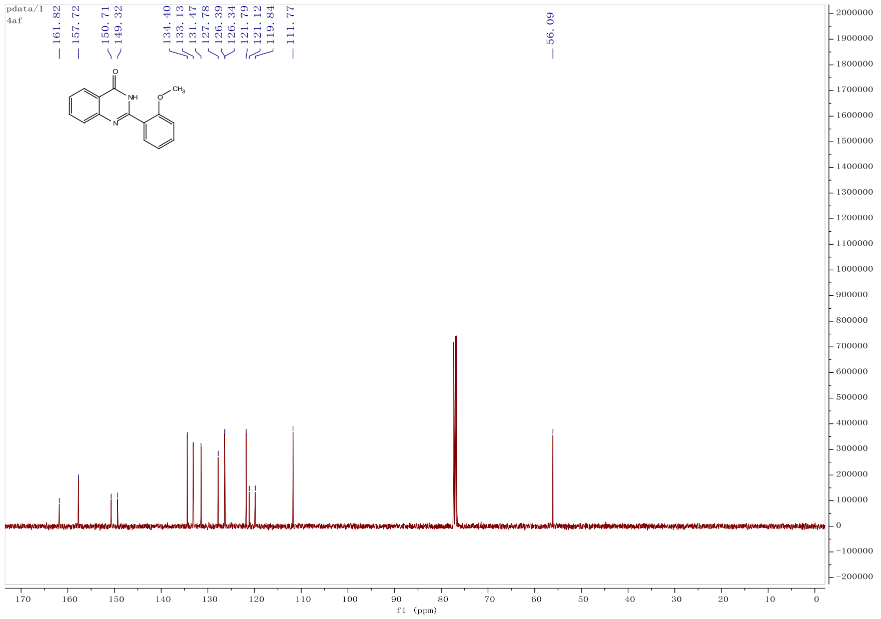

Figure S13 ^{13}C NMR for compound 6 (4af) 2-(2-methoxyphenyl)quinazolin-4(3H)-one.

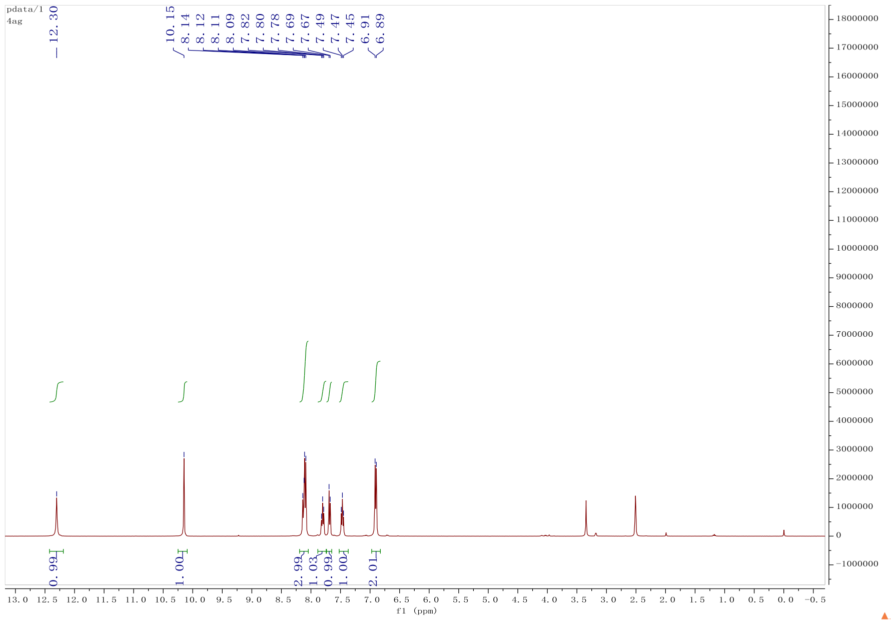

Figure S14

~~Fig. S8.~~ ^{1}H NMR for compound 7 (4ag)2-(4-hydroxyphenyl)quinazolin-4(3H)-one.

Formatted: Font: 10 pt, Not Bold

Formatted: Font: (Default) Times New Roman

Formatted: Font: (Default) Times New Roman, Not Bold

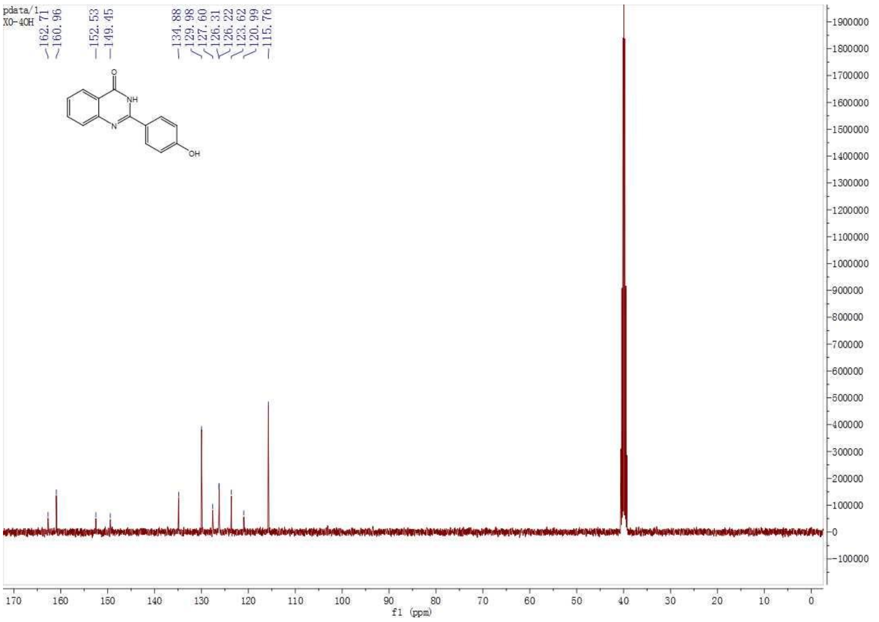

Figure S15

~~Fig. S8.~~ ^{13}C NMR for compound 7 (4ag) 2-(4-hydroxyphenyl)quinazolin-4(3H)-one.

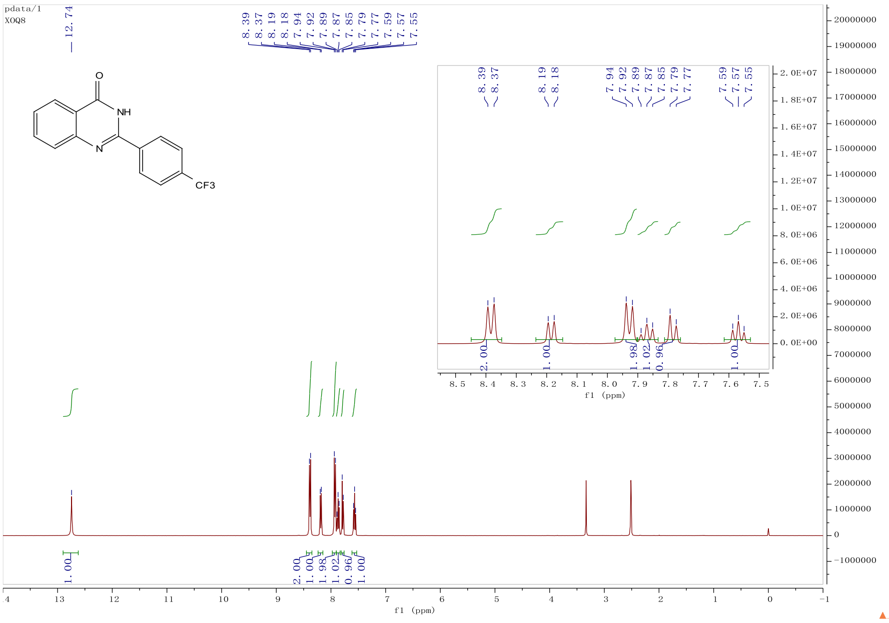

Figure S16

~~Fig. S9.~~ ^{1}H NMR for compound 8 (4ai)2-(4-(trifluoromethyl)phenyl)quinazolin-4(3H)-one.

Formatted: Font: (Default) Times New Roman

Formatted: Font: (Default) Times New Roman, Not Bold

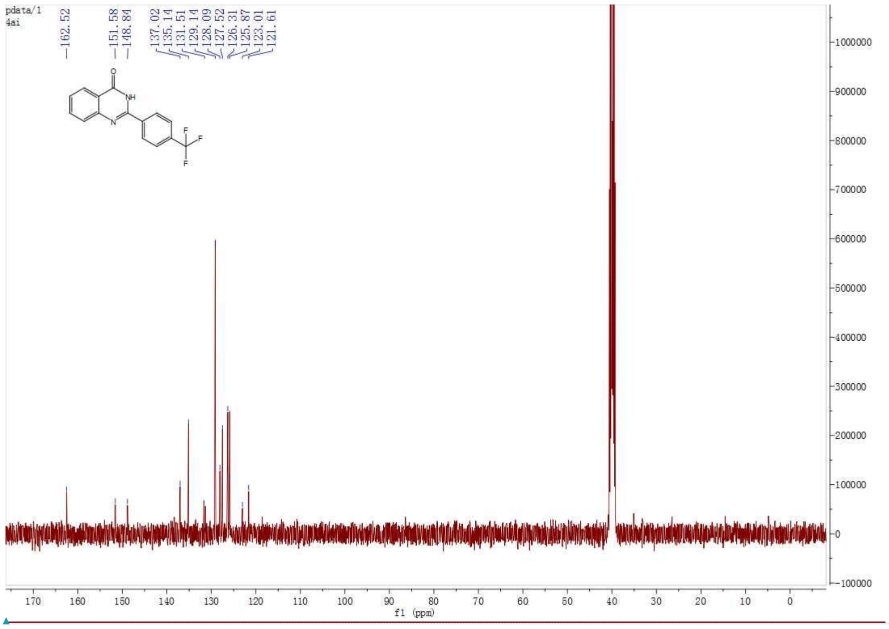

Figure S17 ^{13}C NMR for compound 8 (4ai) 2-(4-(trifluoromethyl)phenyl)quinazolin-4(3H)-one.

Formatted: Font: (Default) Times New Roman, Not Bold

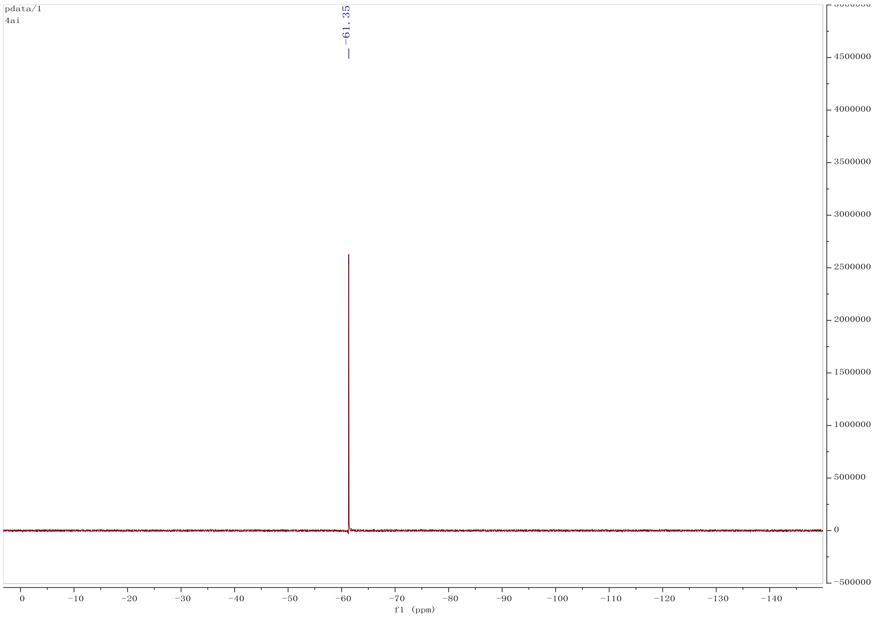

Figure S 18 ^{19}F NMR for compound 8 (4ai) 2-(4-(trifluoromethyl)phenyl)quinazolin-4(3H)-one.

Formatted: Font: (Default) +Body (Calibri), 10.5 pt, Not

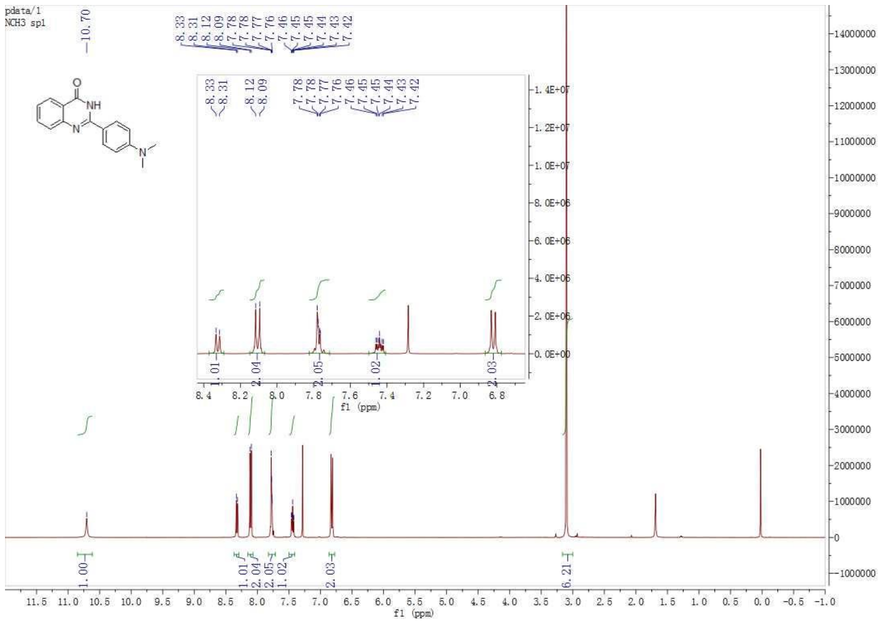

Figure S19

~~Fig. S10.~~ ^{1}H NMR for compound 9 (4aj)2-(4-(dimethylamino)phenyl)quinazolin-4(3H)-one.

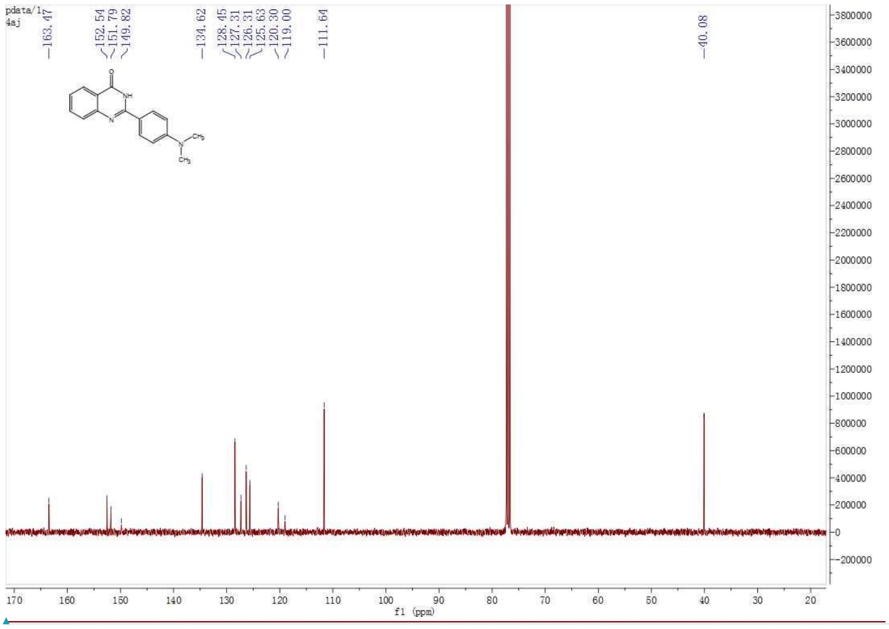

Figure S20 ^{13}C NMR for compound 9 (4aj) 2-(4-(dimethylamino)phenyl)quinazolin-4(3H)-one.

Formatted: Font: (Default) Times New Roman

Formatted: Font: (Default) Times New Roman, Not Bold

Formatted: Font: (Default) Times New Roman, 12 pt, No Bold

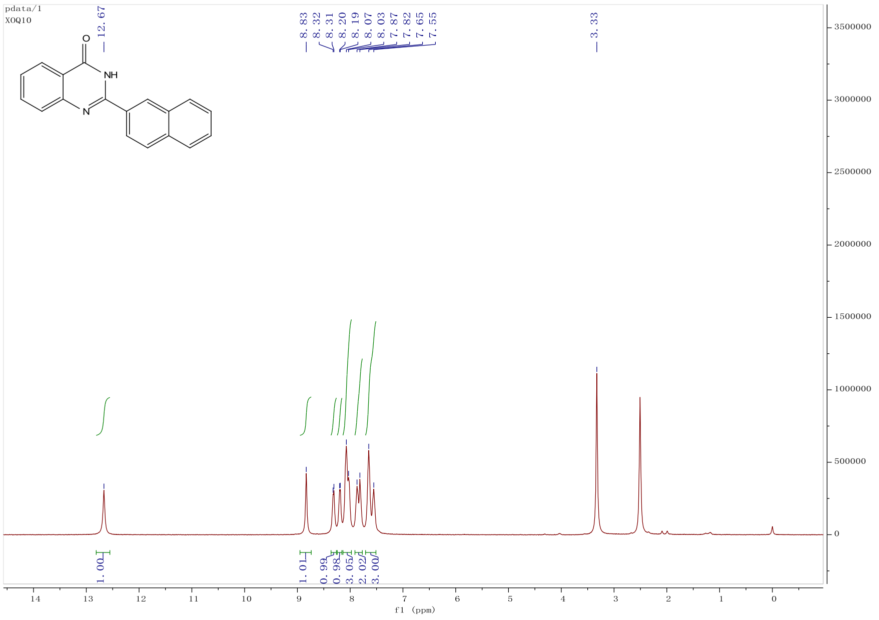

Figure S21
~~Fig. S11.~~ ^{1}H NMR for compound 10 (4ak)2-(naphthalen-2-yl)quinazolin-4(3H)-one.

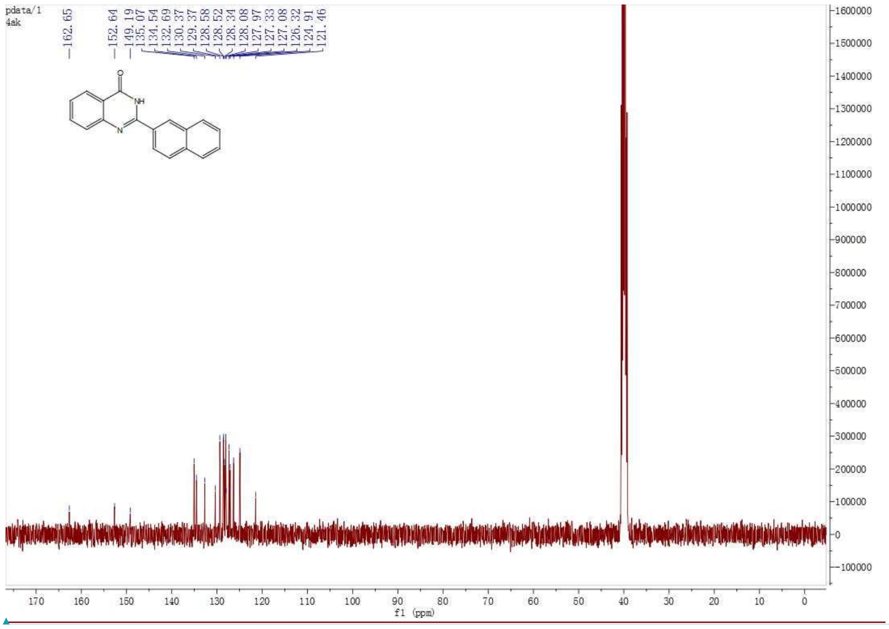

Figure S22 ^{13}C NMR for compound 10 (4ak) 2-(naphthalen-2-yl)quinazolin-4(3H)-one.

Formatted: Font: (Default) Times New Roman

Formatted: Font: (Default) Times New Roman, Not Bold

Formatted: Font: (Default) Times New Roman, Not Bold

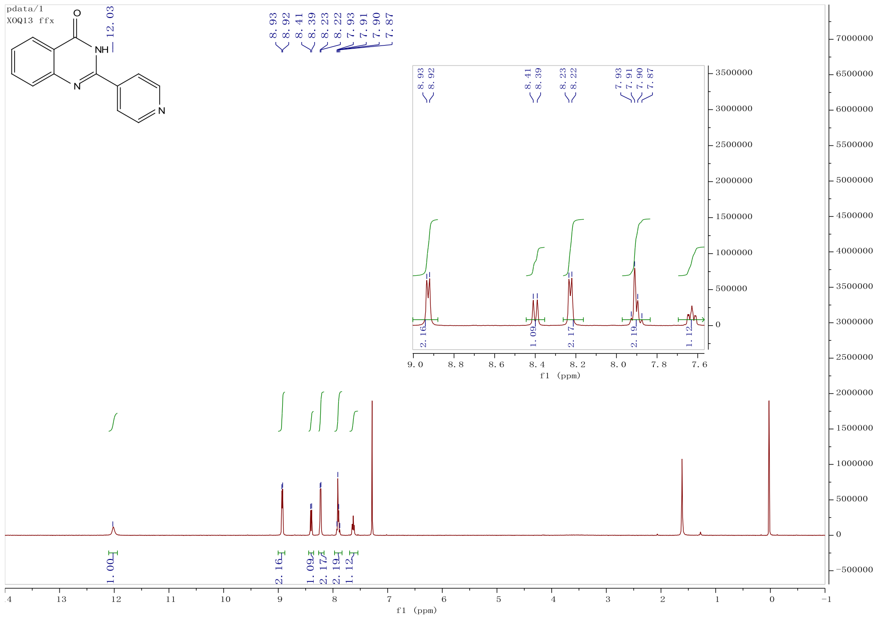

Formatted: Font: (Default) Times New Roman

Figure S23

Fig. S12. ^{1}H NMR for compound 11 (4al)2-(pyridin-4-yl)quinazolin-4(3H)-one.

Formatted: Font: (Default) Times New Roman, Not Bold

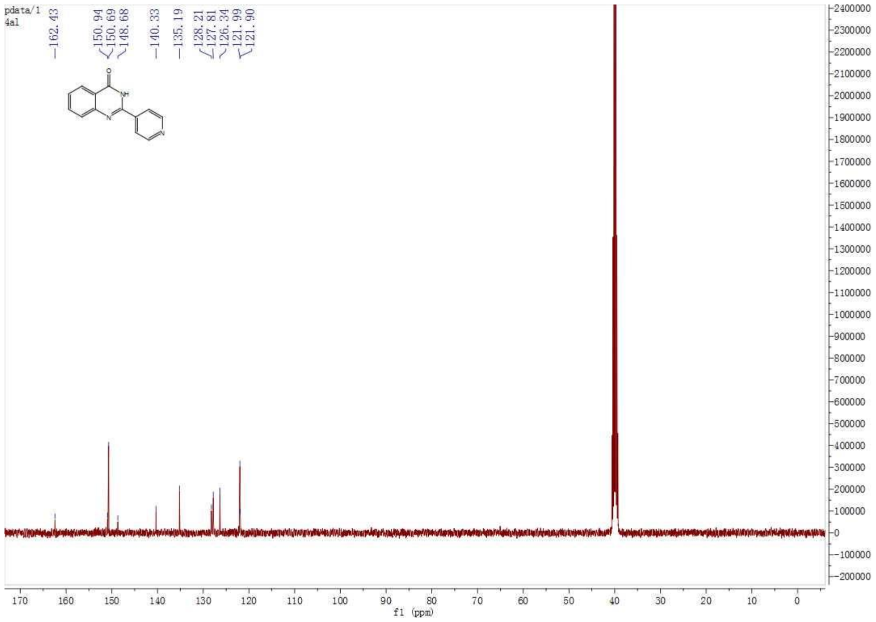

Figure S24Fig. S12. ^{13}C NMR for compound 11 (4al) 2-(pyridin-4-yl)quinazolin-4(3H)-one.

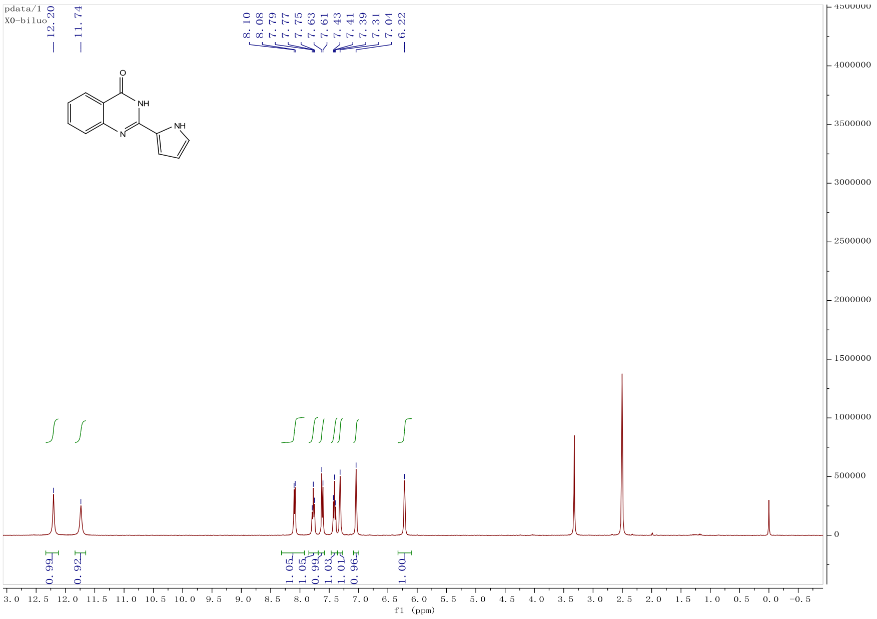

Figure S25

~~Fig. S13.~~ ^{1}H NMR for compound 12 (4ap)2-(1H-pyrrol-2-yl)quinazolin-4(3H)-one.

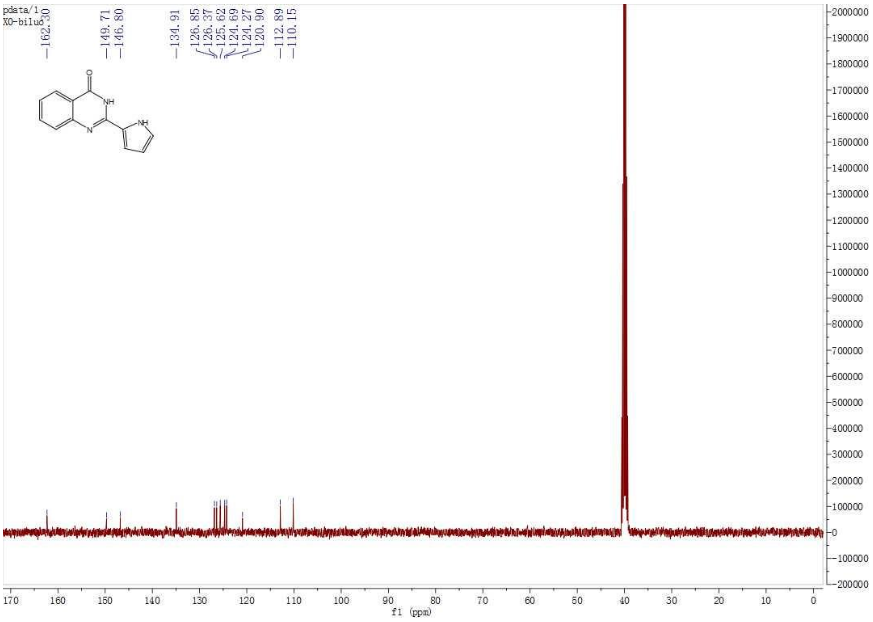

Figure S26 ^{13}C NMR for compound 12 (4ap) 2-(1H-pyrrol-2-yl)quinazolin-4(3H)-one.

Formatted: Font: (Default) Times New Roman

Formatted: Font: (Default) Times New Roman, Not Bold

Formatted: Font: (Default) Times New Roman

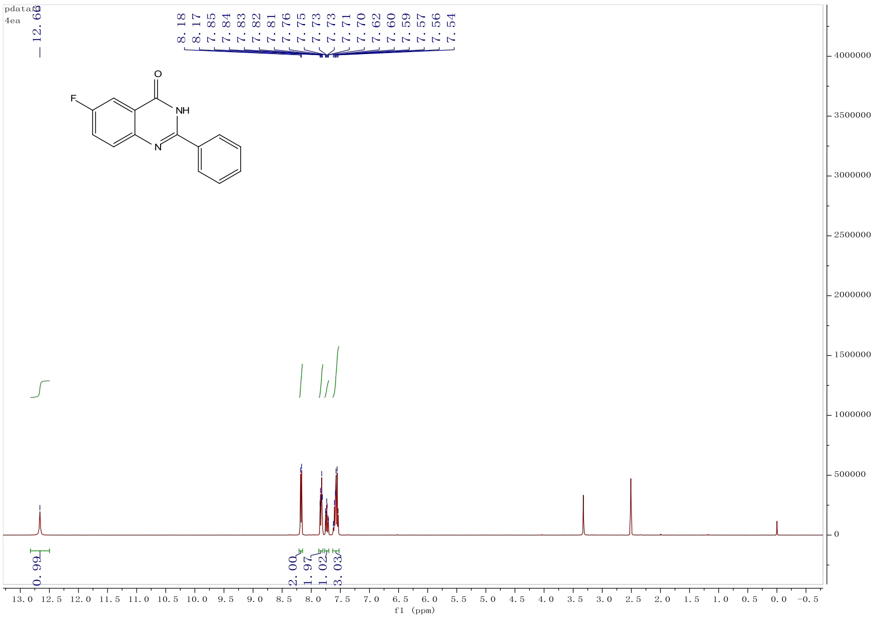

Figure S27

Formatted: Font: (Default) Times New Roman, Not Bold

Fig. S14. ^{1}H NMR for compound 13 (4ea)6-fluoro-2-phenylquinazolin-4(3H)-one.

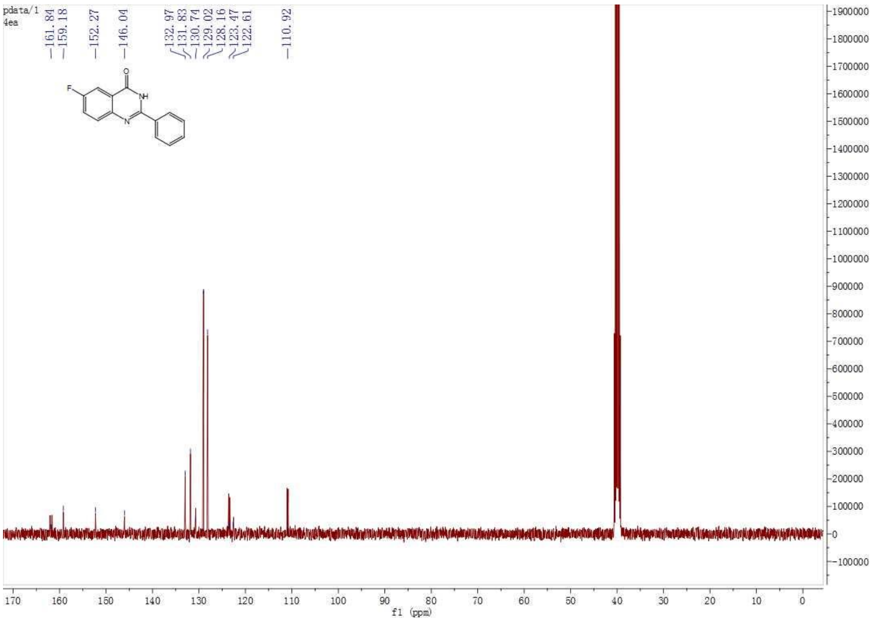

Figure S28Fig. S14. ^{13}C NMR for compound 13 (4ea) 6-fluoro-2-phenylquinazolin-4(3H)-one.

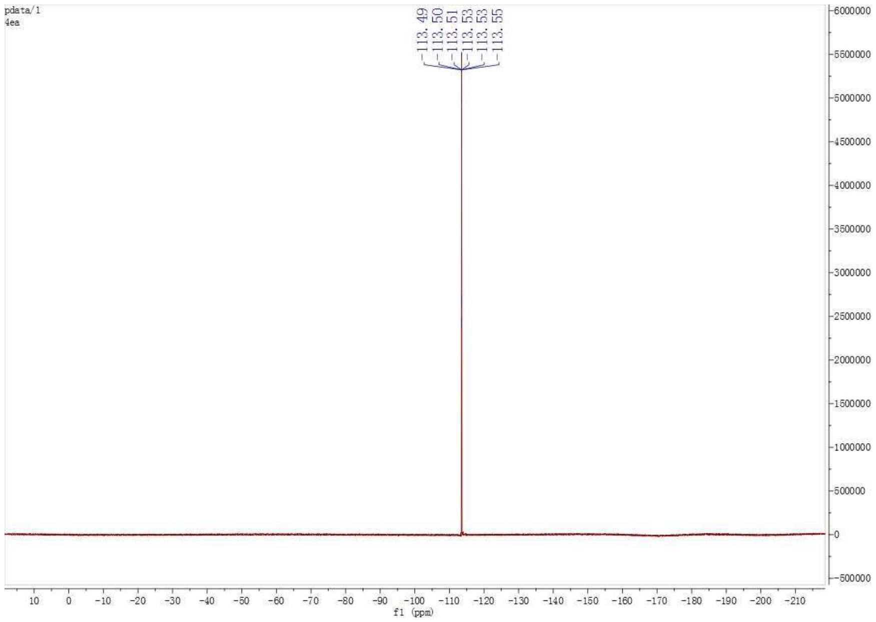

Figure S 29[19]F NMR for compound 13 (4ea) 6-fluoro-2-phenylquinazolin-4(3H)-one.

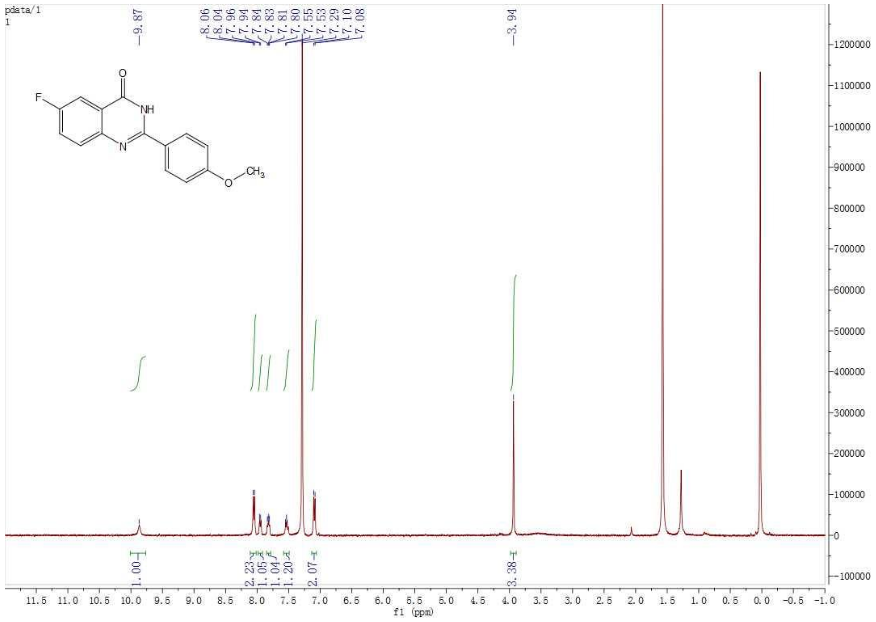

Figure S30

~~Fig. S15.~~ ^{1}H NMR for compound 14 (4ee)6-fluoro-2-(4-methoxyphenyl)quinazolin-4(3H)-one.

Formatted: Font: (Default) Times New Roman

Formatted: Font: (Default) Times New Roman, Not Bold

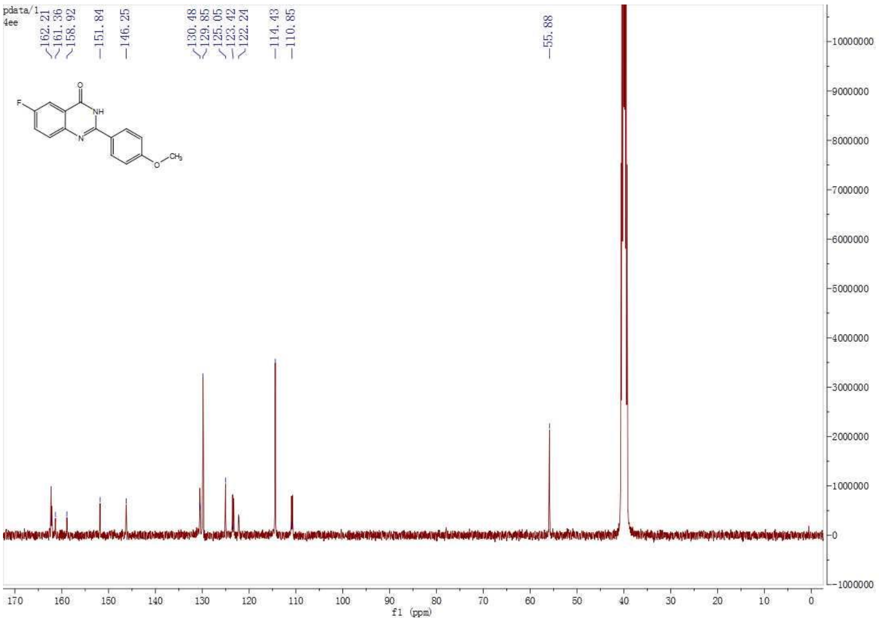

Figure S 31[13]C NMR for compound 14 (4ee) 6-fluoro-2-(4-methoxyphenyl)quinazolin-4(3H)-one.

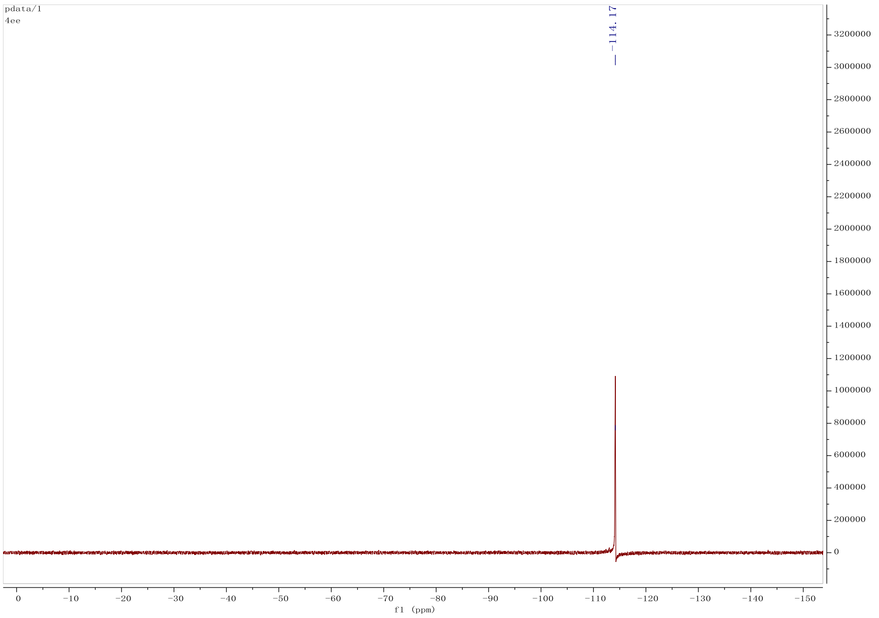

Figure S 32[19]F NMR for compound 14 (4ee) 6-fluoro-2-(4-methoxyphenyl)quinazolin-4(3H)-one.

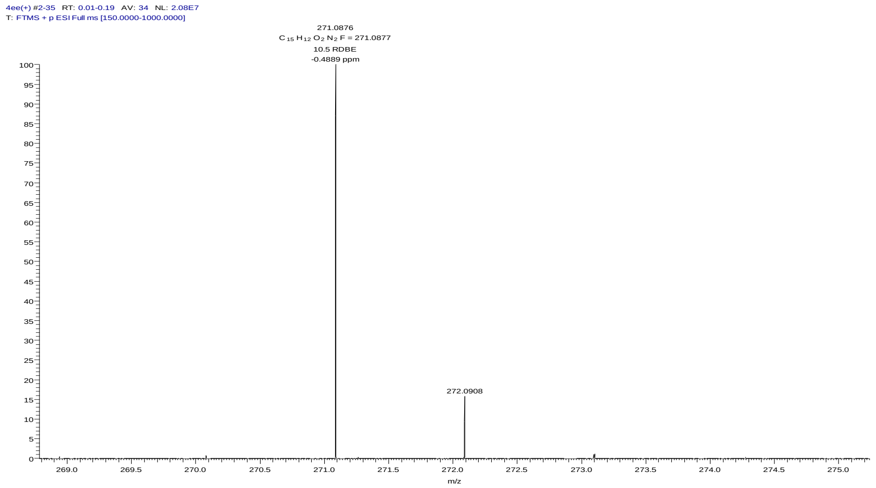

Figure S 33HRMS for compound 14 (4ee) 6-fluoro-2-(4-methoxyphenyl)quinazolin-4(3H)-one.

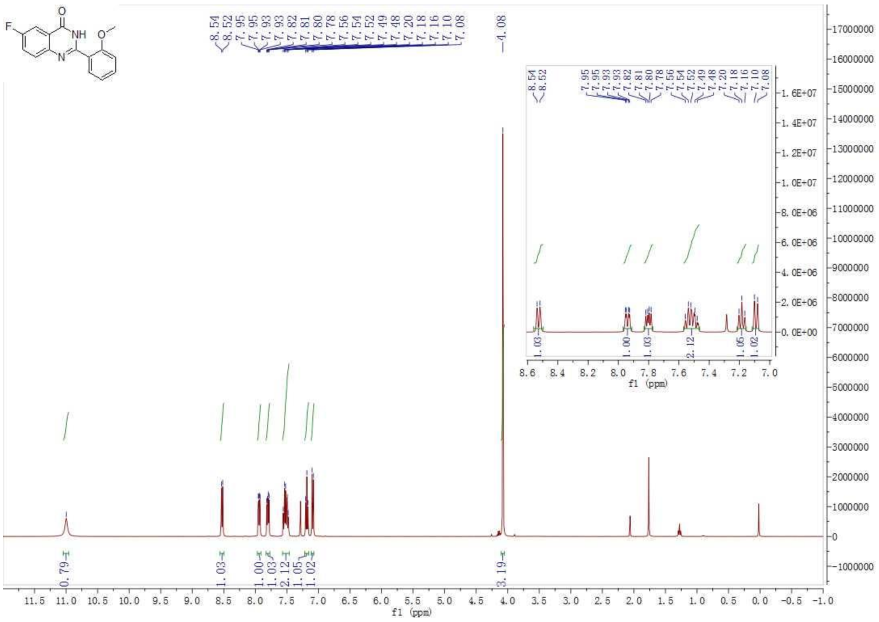

Figure S34

~~Fig. S16.~~ ^{1}H NMR for compound 15 (4ef)6-fluoro-2-(2-methoxyphenyl)quinazolin-4(3H)-one.

Formatted: Font: (Default) Times New Roman

Formatted: Font: (Default) Times New Roman, Not Bold

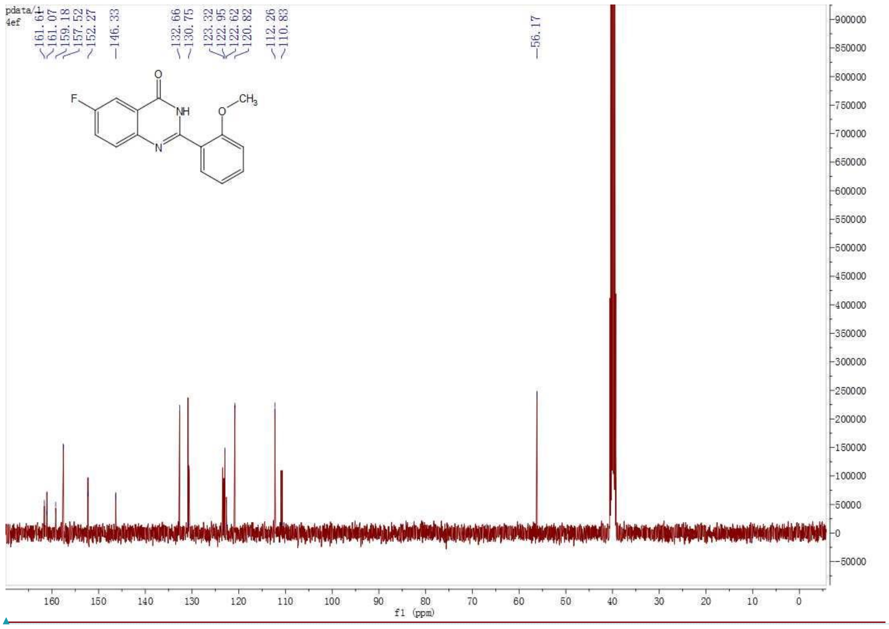

Figure S35[13]C NMR for compound 15 (4ef) 6-fluoro-2-(2-methoxyphenyl)quinazolin-4(3H)-one.

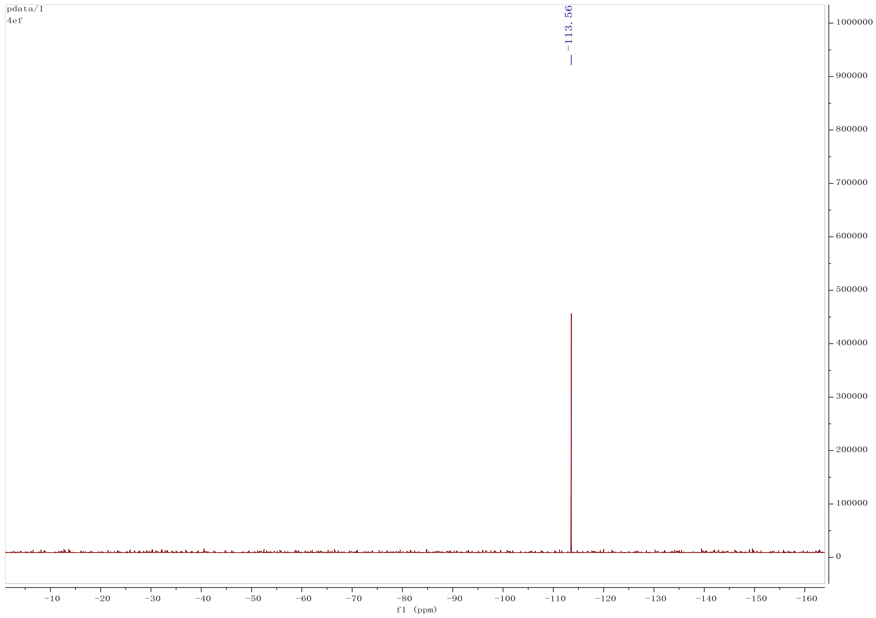

Figure S 36[19]F NMR for compound 15 (4ef) 6-fluoro-2-(2-methoxyphenyl)quinazolin-4(3H)-one.

Formatted: Font: (Default) Times New Roman, Not Bold

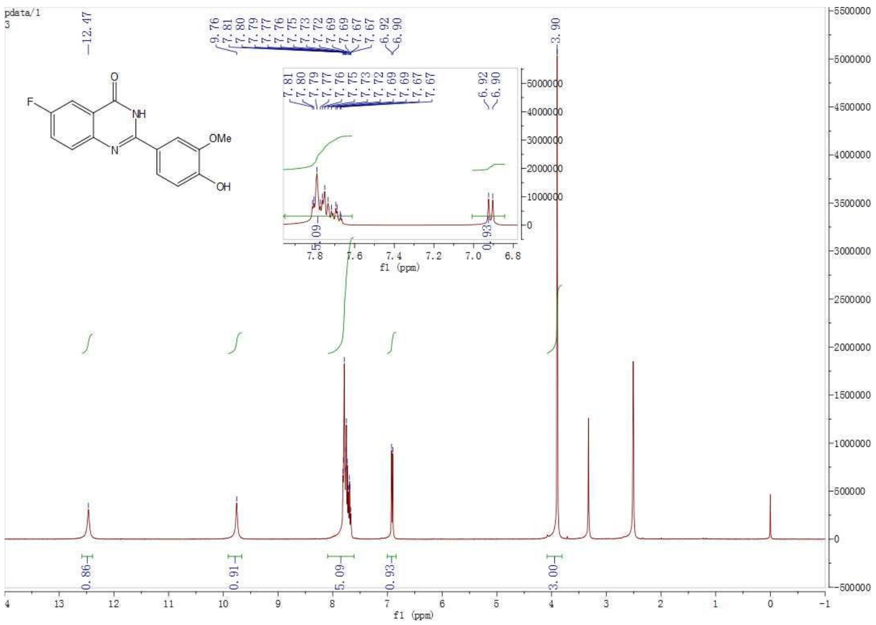

Figure S37

Fig.S17.^{1}HNMRforcompound 16 (4eq)6-fluoro-2-(4-hydroxy-3-methoxyphenyl)quinazolin-4(3H)-one.

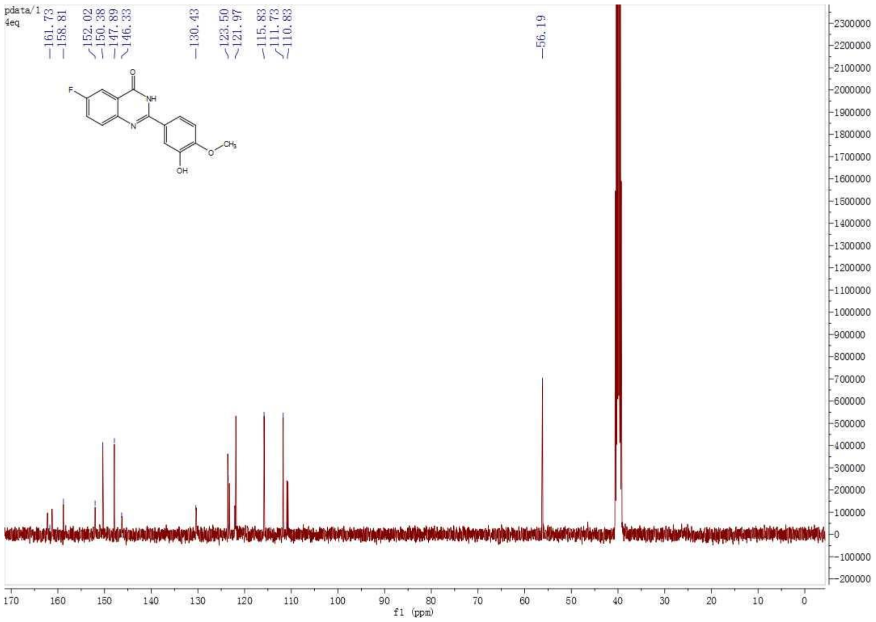

Figure S38Fig.S17.^{13}CNMRforcompound 16 (4eq) 6-fluoro-2-(4-hydroxy-3-methoxyphenyl)quinazolin-4(3H)-one.

Formatted: Font: (Default) Times New Roman

Formatted: Font: (Default) Times New Roman, Not Bold

Formatted: Superscript

Formatted: Font: Not Bold, Superscript

Formatted: Font: Not Bold, Superscript

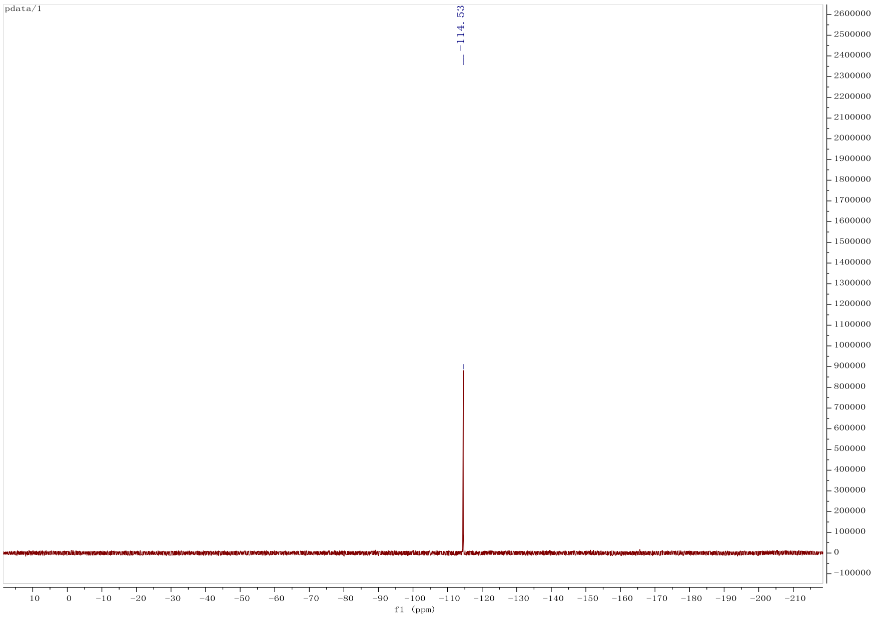

Figure S 39 ^{19}FNMRforcompound 16 (4eq) 6-fluoro-2-(4-hydroxy-3-methoxyphenyl)quinazolin-4(3H)-one.

Formatted: Font: (Asian) 黑体, 10 pt, Not Bold, Superscr

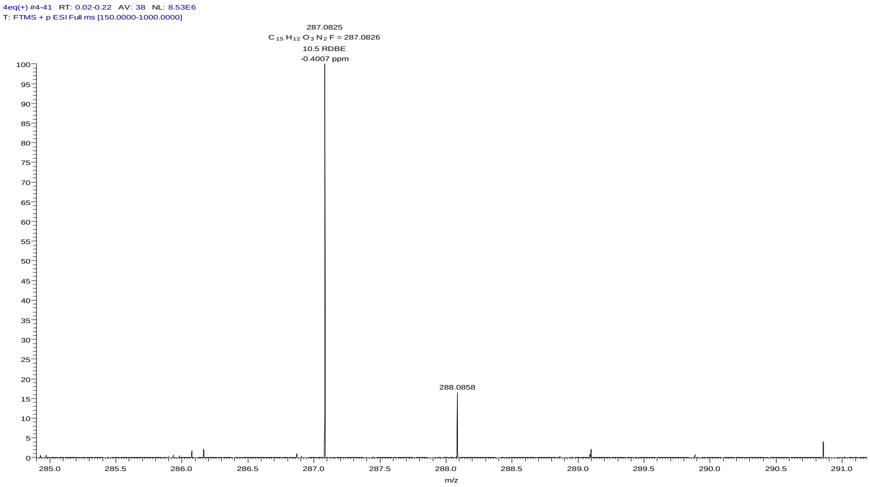

Figure S40HRMSforcompound 16 (4eq) 6-fluoro-2-(4-hydroxy-3-methoxyphenyl)quinazolin-4(3H)-one.

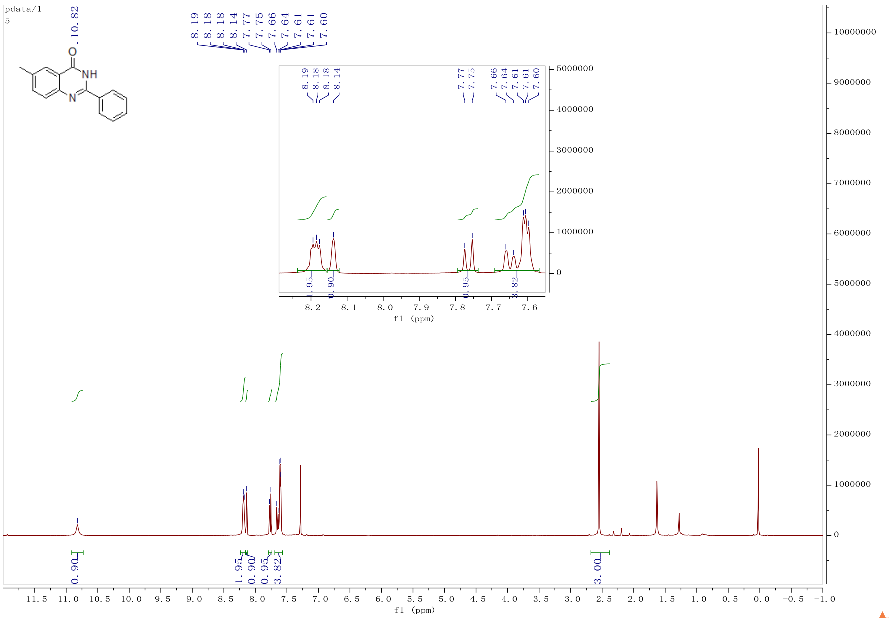

~~Figure S~~41

~~Fig.S18.~~ ^{1}HNMRforcompound 17 (4fa)6-methyl-2-phenylquinazolin-4(3H)-one.

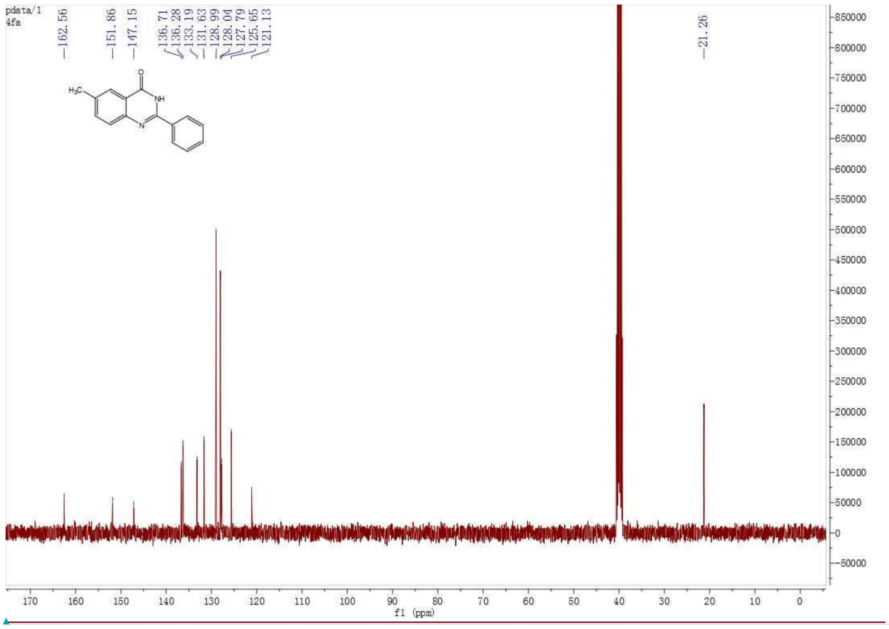

Figure S42^{13}C NMRforcompound 17 (4fa) 6-methyl-2-phenylquinazolin-4(3H)-one.

Formatted: Font: (Default) Times New Roman

Formatted: Font: (Default) Times New Roman, Not Bold

Formatted: Font: (Default) Times New Roman, Not Bold

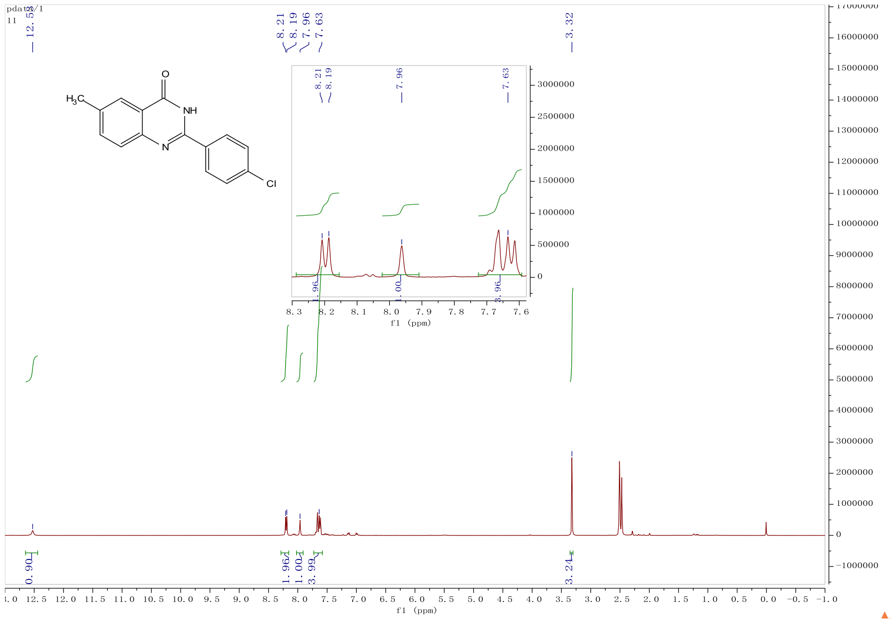

Figure S43

~~Fig. S19.~~ ^{1}H NMR for compound 18 (4fb)2-(4-chlorophenyl)-6-methylquinazolin-4(3H)-one.

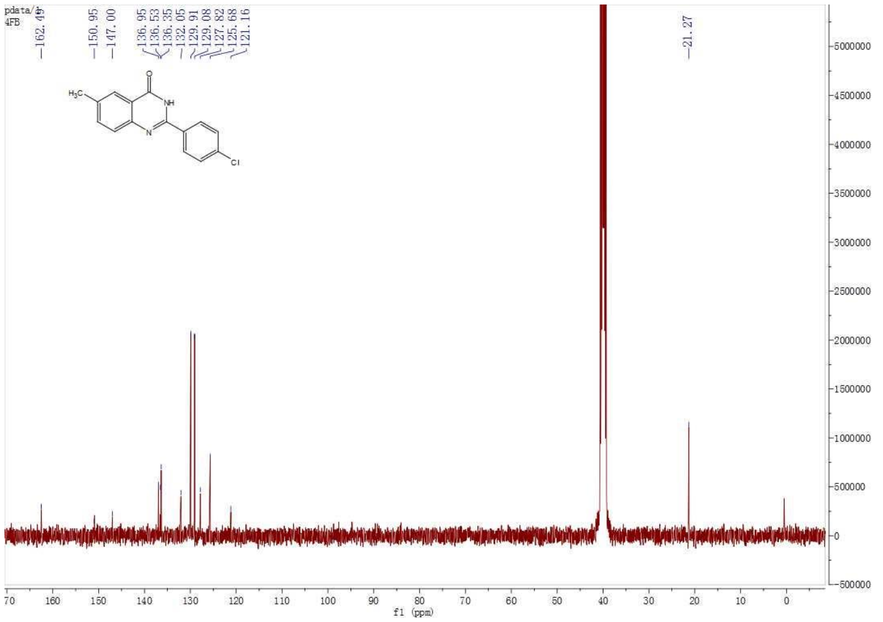

Figure S44~~Fig. S19.~~ ^{13}C NMR for compound 18 (4fb) 2-(4-chlorophenyl)-6-methylquinazolin-4(3H)-one.

Formatted: Font: (Default) Times New Roman

Formatted: Font: (Default) Times New Roman, Not Bold

Formatted: Font: Not Bold

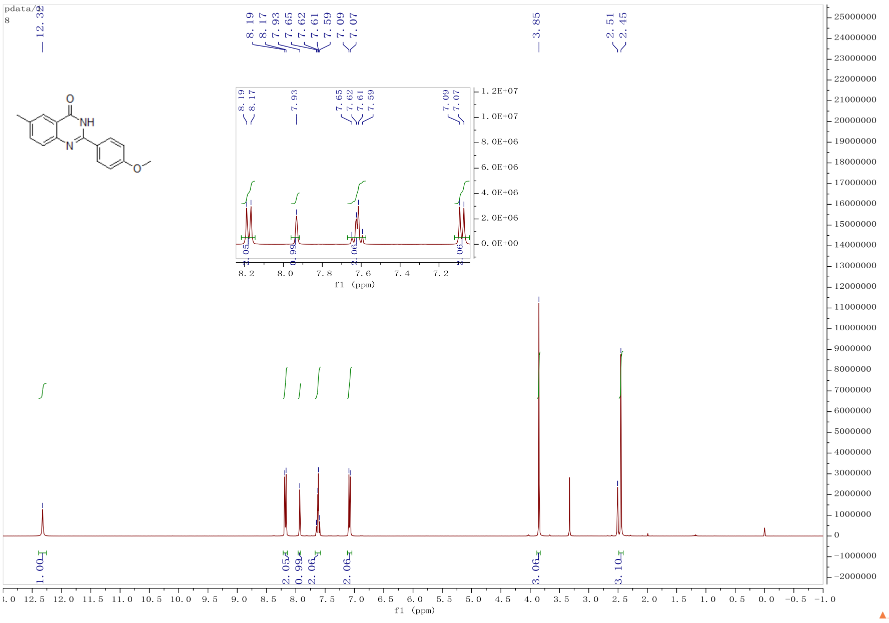

Figure S45

Fig. S20. ^{1}H NMR for compound 19 (4fe)2-(4-methoxyphenyl)-6-methylquinazolin-4(3H)-one.

Formatted: Font: (Default) Times New Roman

Formatted: Font: (Default) Times New Roman, Not Bold

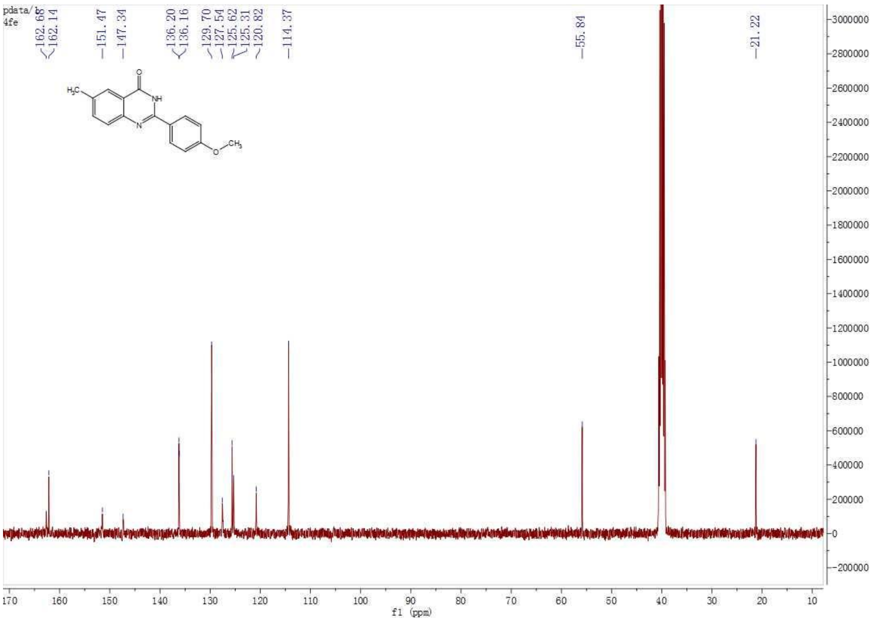

Figure S46Fig. S20. ^{13}C NMR for compound 19 (4fe) 2-(4-methoxyphenyl)-6-methylquinazolin-4(3H)-one.

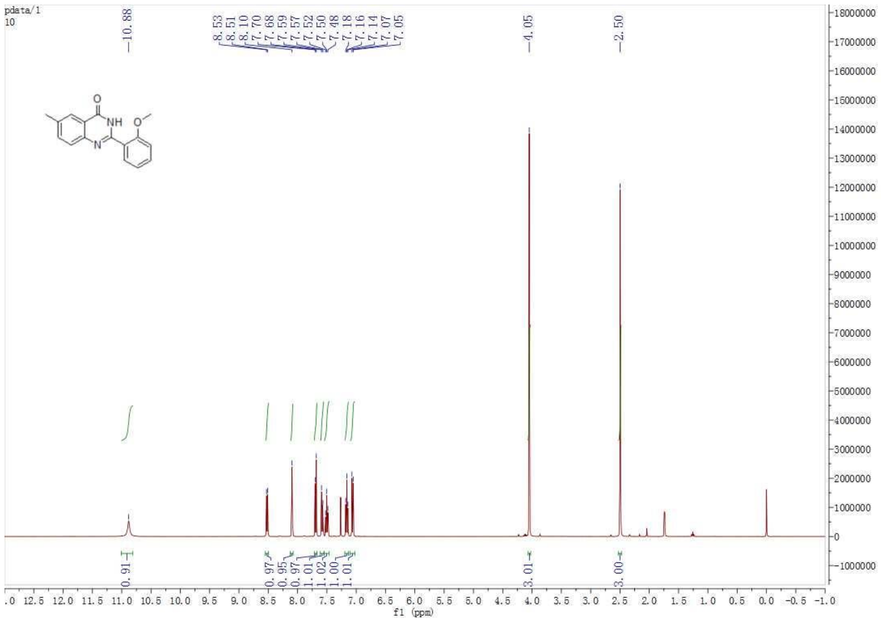

Figure S47

Fig. S21. ^{1}H NMR for compound 20 (4ff)2-(2-methoxyphenyl)-6-methylquinazolin-4(3H)-one.

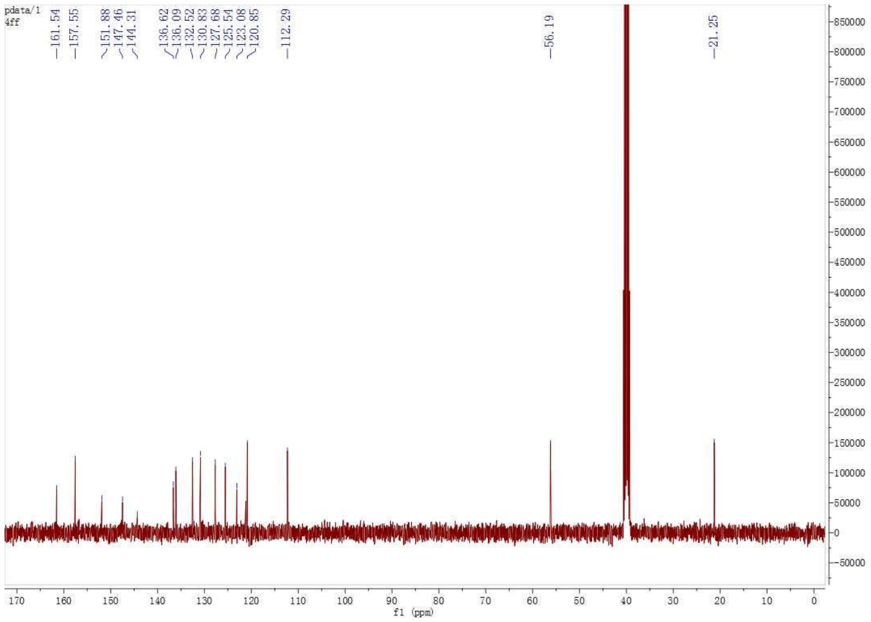

Figure S48Fig. S21. ^{13}C NMR for compound 20 (4ff) 2-(2-methoxyphenyl)-6-methylquinazolin-4(3H)-one.

Formatted: Font: (Default) Times New Roman

Formatted: Font: (Default) Times New Roman, Not Bold

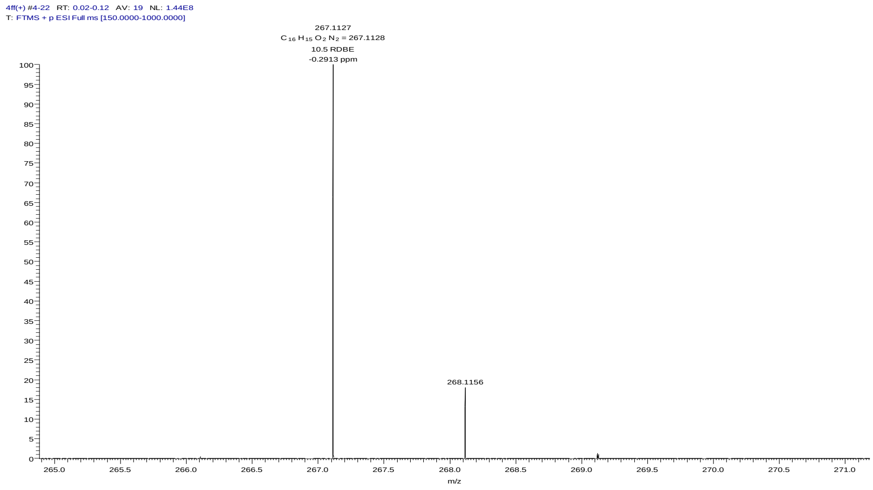

Figure S49HRMSfor compound 20 (4ff) 2-(2-methoxyphenyl)-6-methylquinazolin-4(3H)-one.

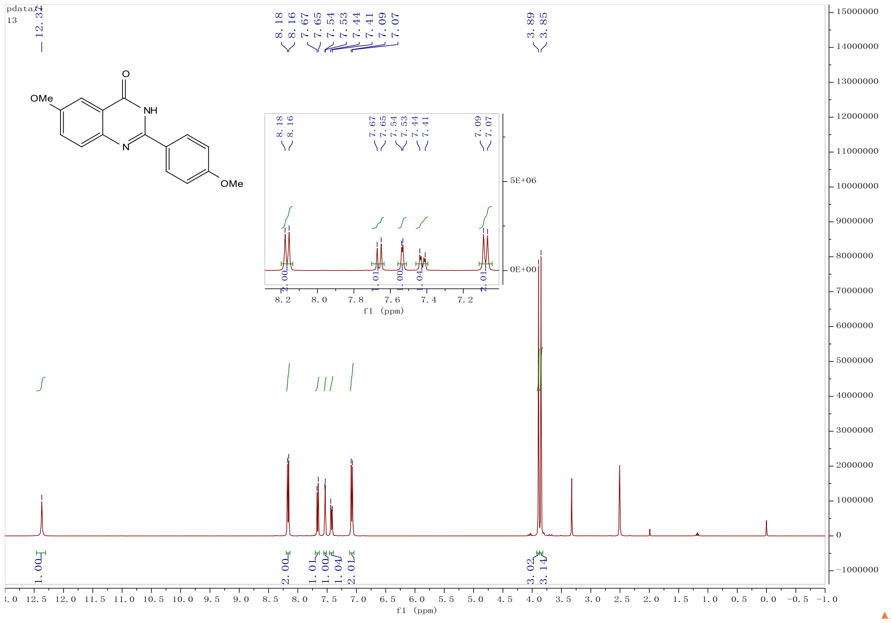

Figure S50

~~Fig. S22.~~ ^{1}H NMR for compound 21 (4ge)6-methoxy-2-(4-methoxyphenyl)quinazolin-4(3H)-one.

Formatted: Font: (Default) Times New Roman

Formatted: Font: (Default) Times New Roman, Not Bold

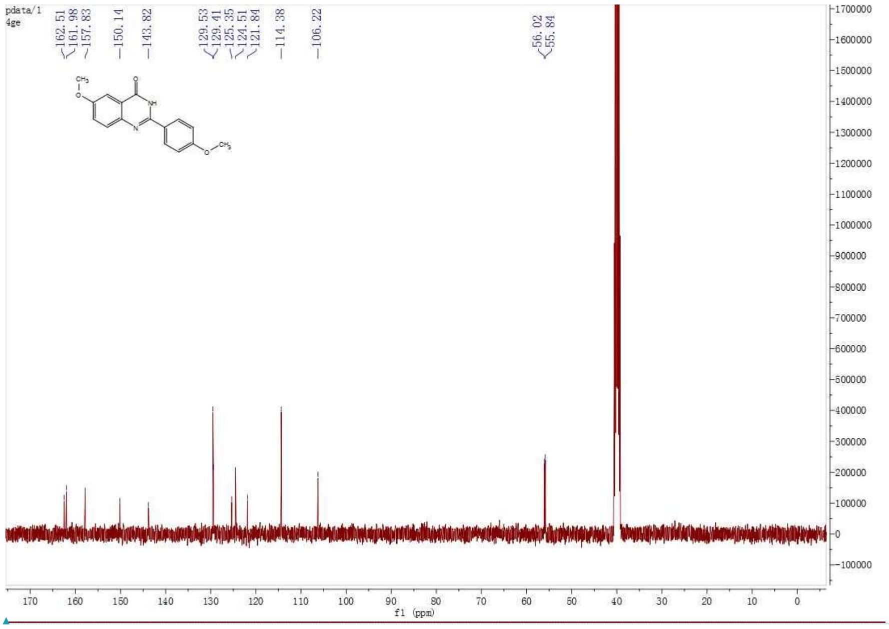

Figure S51 ^{13}C NMR for compound 21 (4ge) 6-methoxy-2-(4-methoxyphenyl)quinazolin-4(3H)-one.

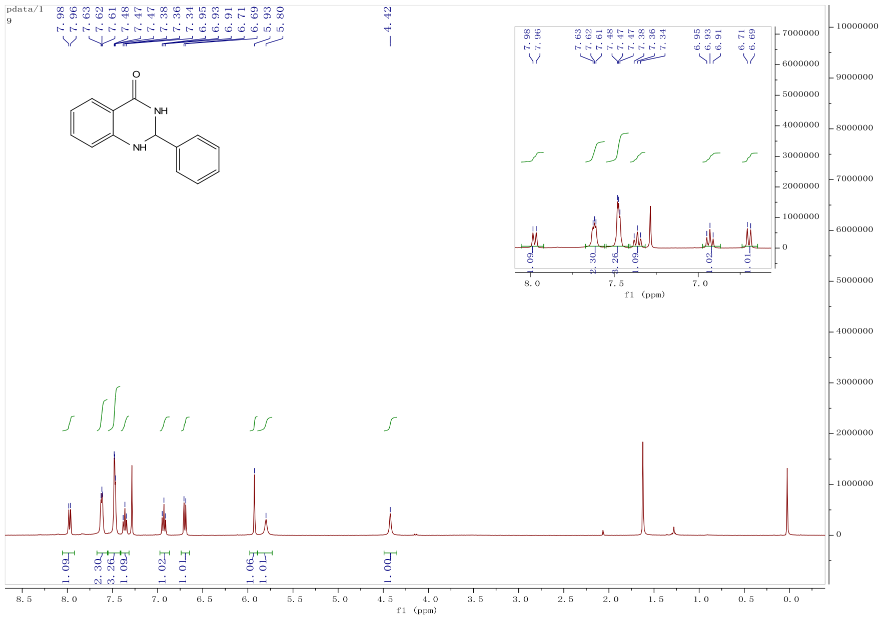

Figure S52

~~Fig. S23.~~ ^{1}H NMR for compound 22 (5aa)2-phenyl-2,3-dihydroquinazolin-4(1H)-one.

Formatted: Font: (Default) Times New Roman, Not Bold

Formatted: Font: (Default) Times New Roman

Formatted: Font: (Default) Times New Roman, Not Bold

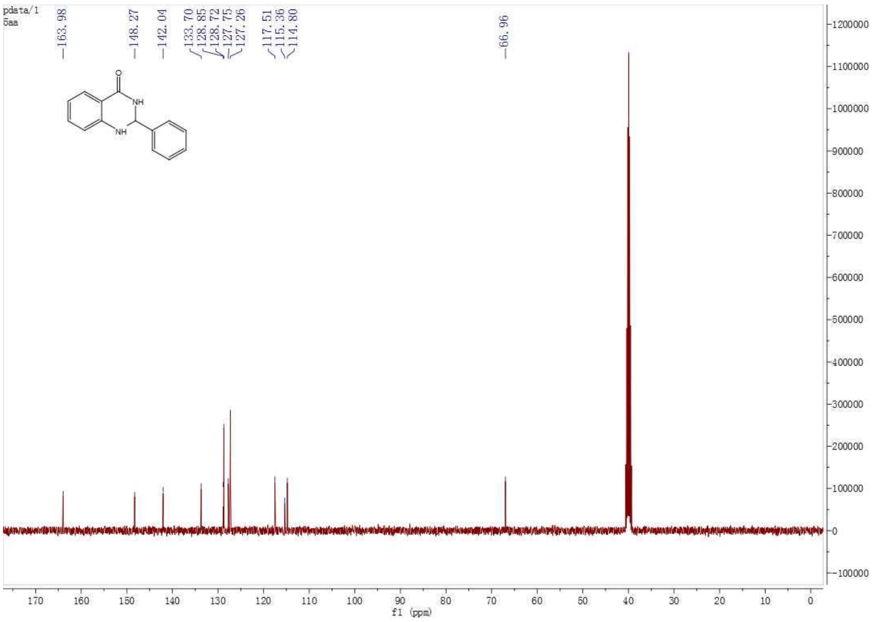

Figure S53 ^{13}C NMR for compound 22 (5aa) 2-phenyl-2,3-dihydroquinazolin-4(1H)-one.

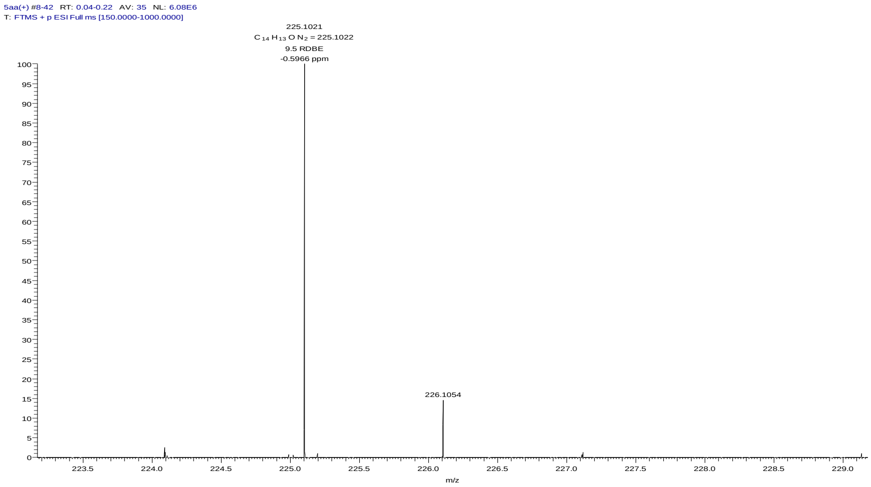

Figure S54HRMS for compound 22 (5aa) 2-phenyl-2,3-dihydroquinazolin-4(1H)-one.

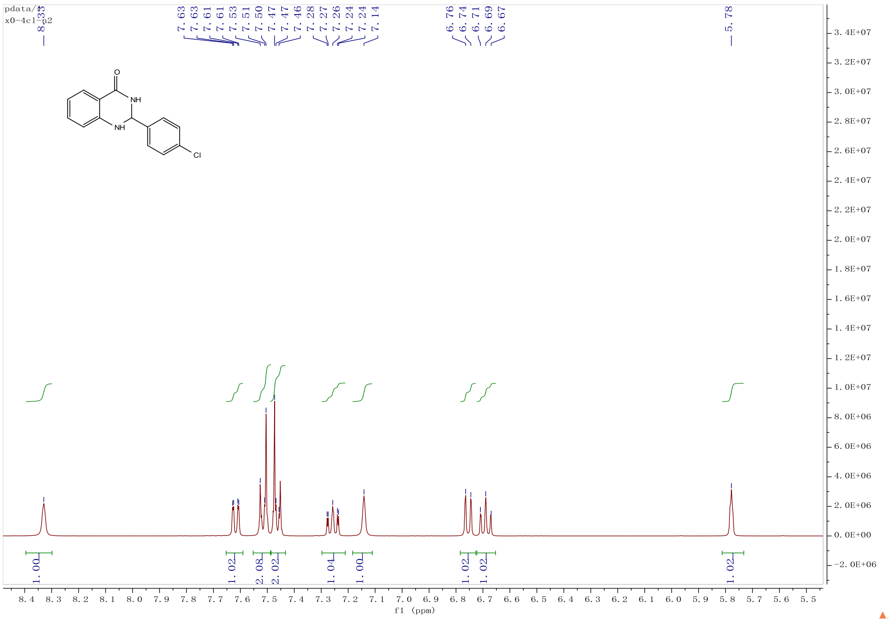

Figure S55

~~Fig. S24.~~ ^{1}H NMR for compound 23 (5ab)2-(4-chlorophenyl)-2,3-dihydroquinazolin-4(1H)-one.

Formatted: Font: (Default) Times New Roman

Formatted: Font: (Default) Times New Roman, Not Bold

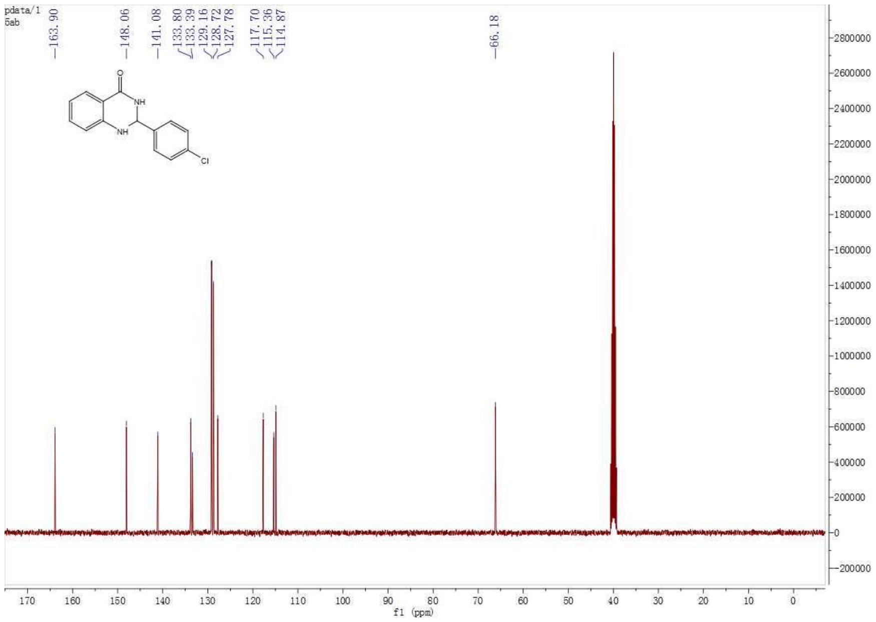

Figure S56 ~~Fig. S24.~~ ^{13}C NMR for compound 23 (5ab) 2-(4-chlorophenyl)-2,3-dihydroquinazolin-4(1H)-one.

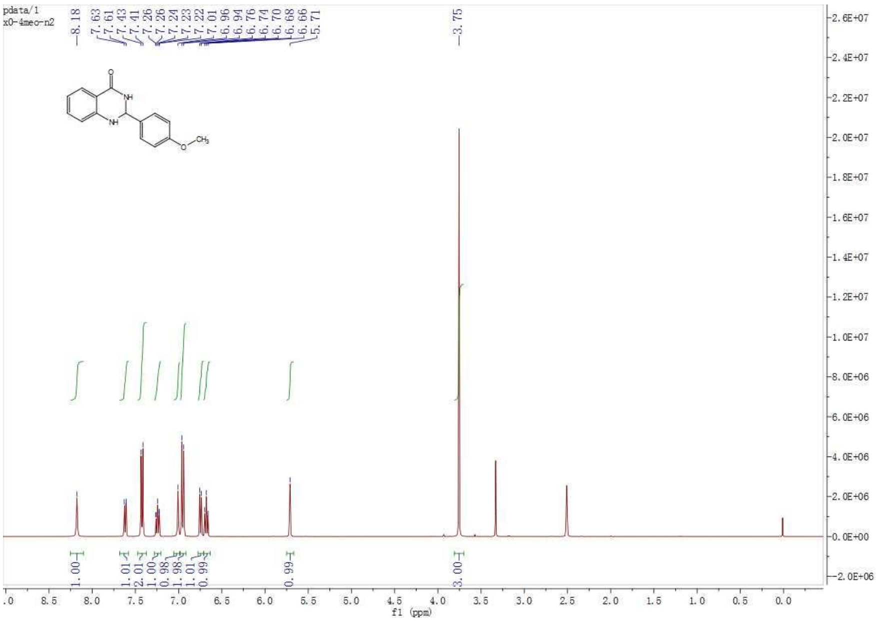

Figure S57

Fig. S25. ^{1}H NMR for compound 24 (5ae)2-(4-methoxyphenyl)-2,3-dihydroquinazolin-4(1H)-one.

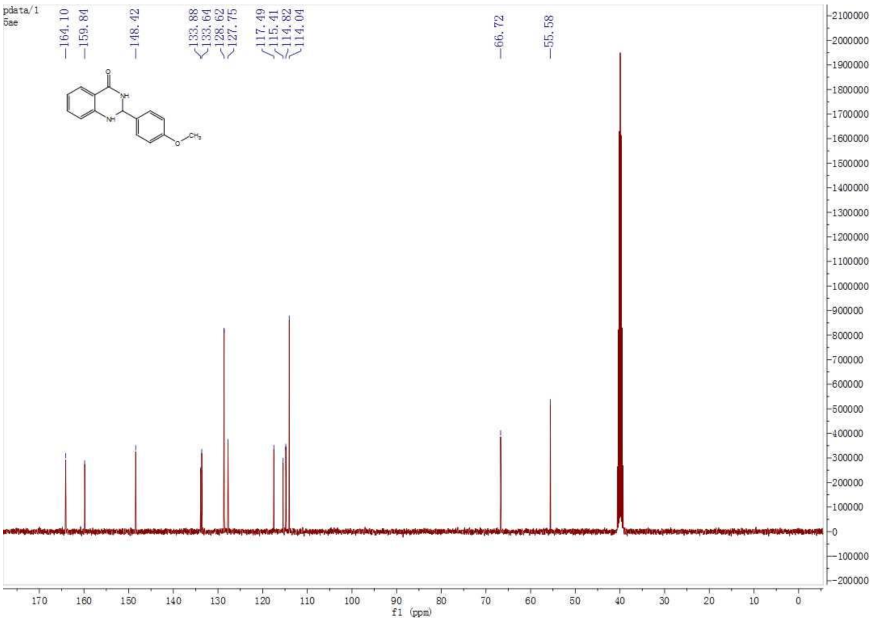

Figure S58 Fig. S25. ^{13}C NMR for compound 24 (5ae) 2-(4-methoxyphenyl)-2,3-dihydroquinazolin-4(1H)-one.

Formatted: Font: (Default) Times New Roman

Formatted: Font: (Default) Times New Roman, Not Bold

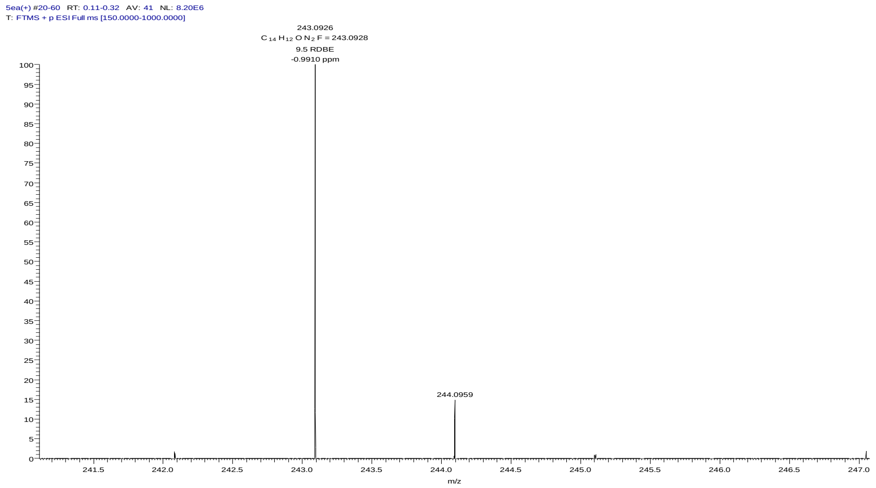

Figure S59HRMS for compound 24 (5ae) 2-(4-methoxyphenyl)-2,3-dihydroquinazolin-4(1H)-one.

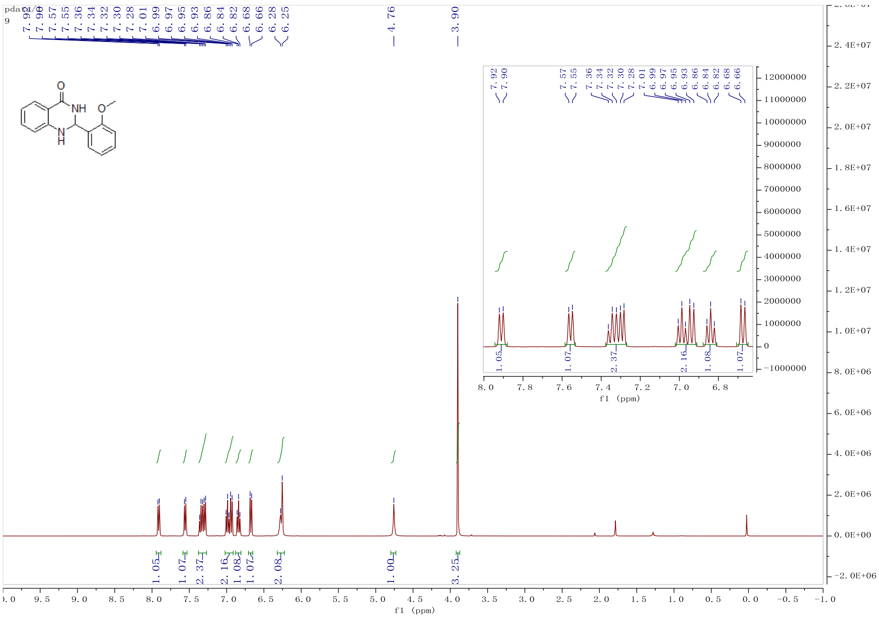

Figure S60

Fig. S26. ^{1}H NMR for compound 25 (5af)2-(2-methoxyphenyl)-2,3-dihydroquinazolin-4(1H)-one.

Formatted: Font: (Default) Times New Roman

Formatted: Font: (Default) Times New Roman, Not Bold

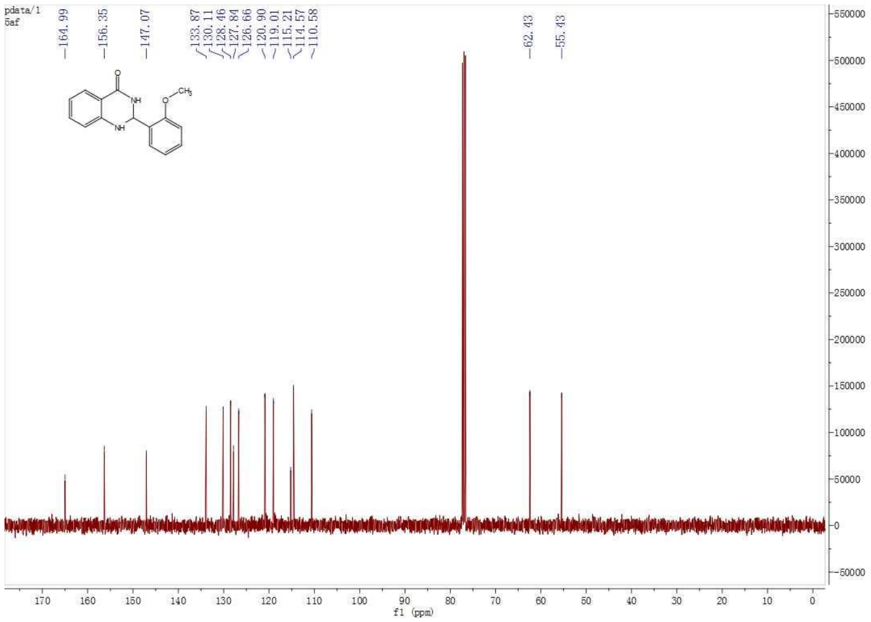

Figure S61~~Fig. S26.~~ ^{13}C NMR for compound 25 (5af) 2-(2-methoxyphenyl)-2,3-dihydroquinazolin-4(1H)-one.

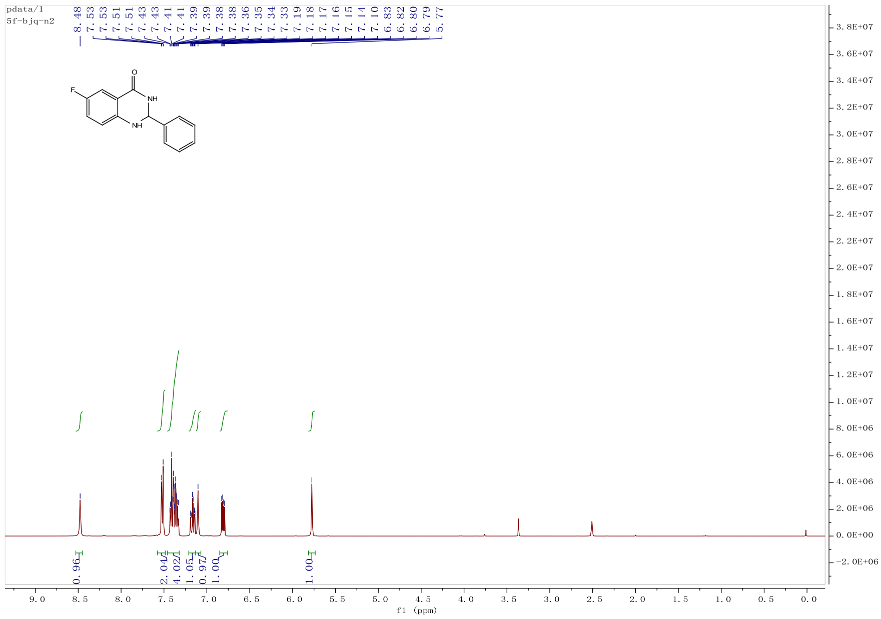

Figure S62

~~Fig. S27.~~ ^{1}H NMR for compound 26 (5ea)6-fluoro-2-phenyl-2,3-dihydroquinazolin-4(1H)-one.

Formatted: Font: (Default) Times New Roman

Formatted: Font: (Default) Times New Roman, Not Bold

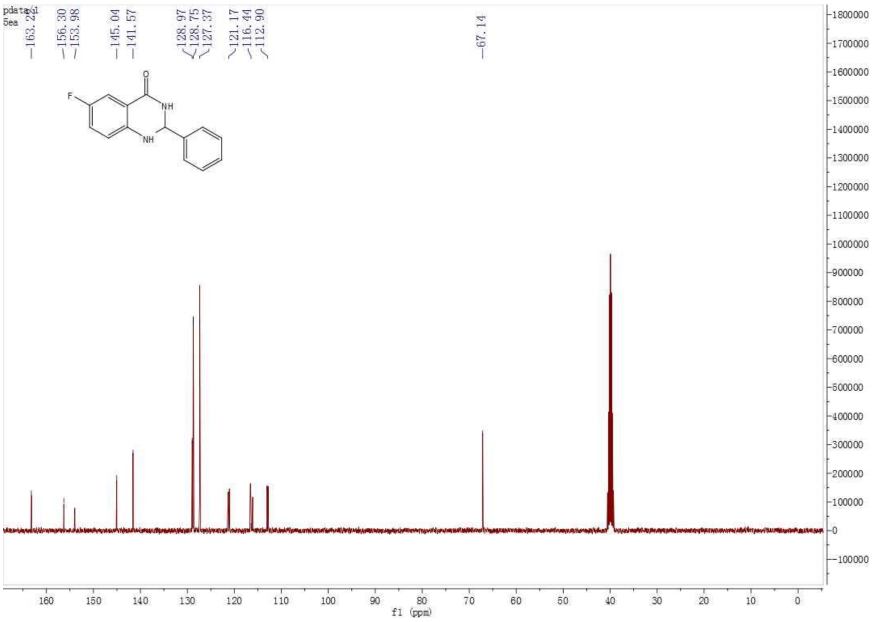

Figure S63~~Fig. S27.~~ ^{13}C NMR for compound 26 (5ea) 6-fluoro-2-phenyl-2,3-dihydroquinazolin-4(1H)-one.

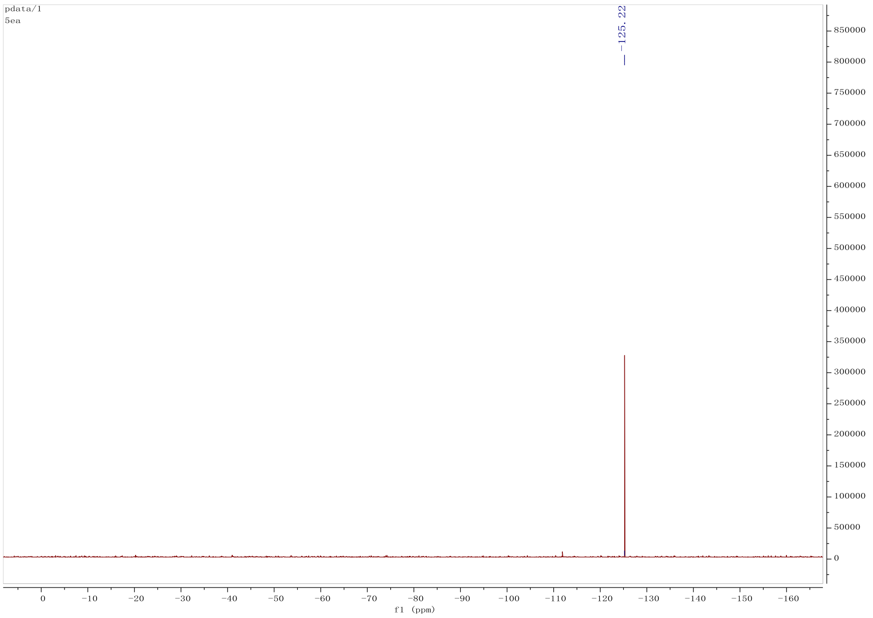

Figure S 64^{19}F NMR for compound 26 (5ea) 6-fluoro-2-phenyl-2,3-dihydroquinazolin-4(1H)-one.

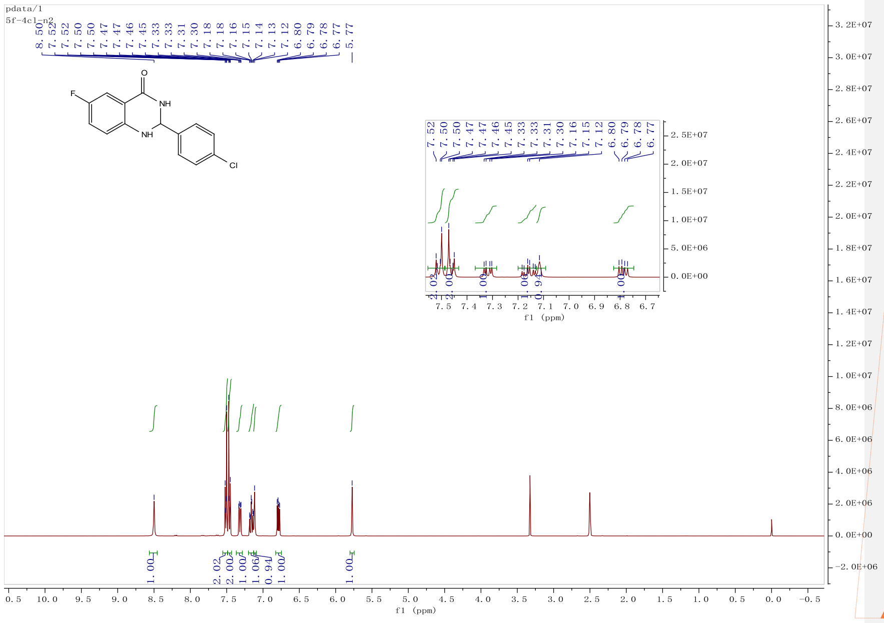

Figure S65

Fig. S28. ^{1}H NMR for compound 27 (5eb)2-(4-chlorophenyl)-6-fluoro-2,3-dihydroquinazolin-4(1H)-one.

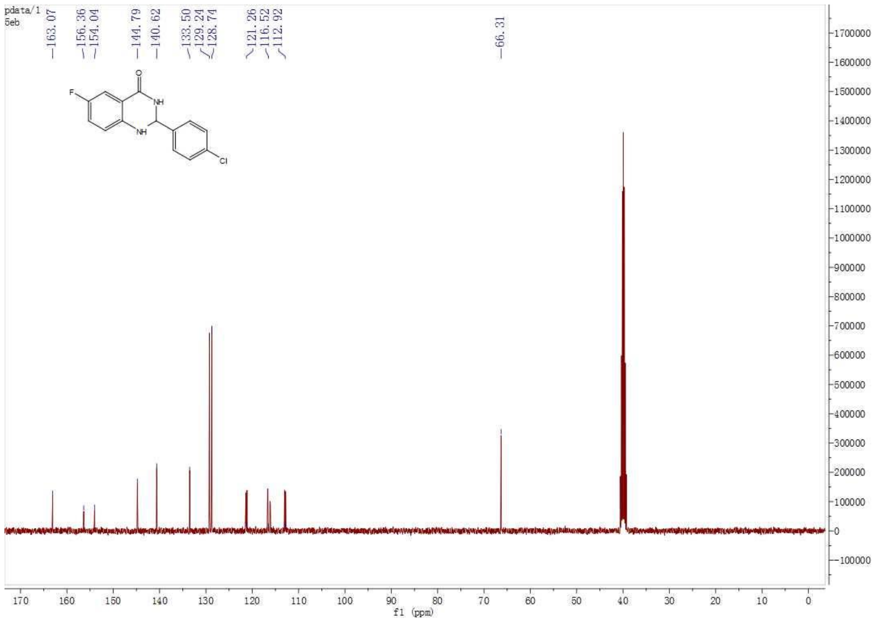

Figure S66Fig. S28. ^{13}C NMR for compound 27 (5eb) 2-(4-chlorophenyl)-6-fluoro-2,3-

Formatted: Font: (Default) Times New Roman

Formatted: Font: (Default) Times New Roman, Not Bold

dihydroquinazolin-4(1H)-one.

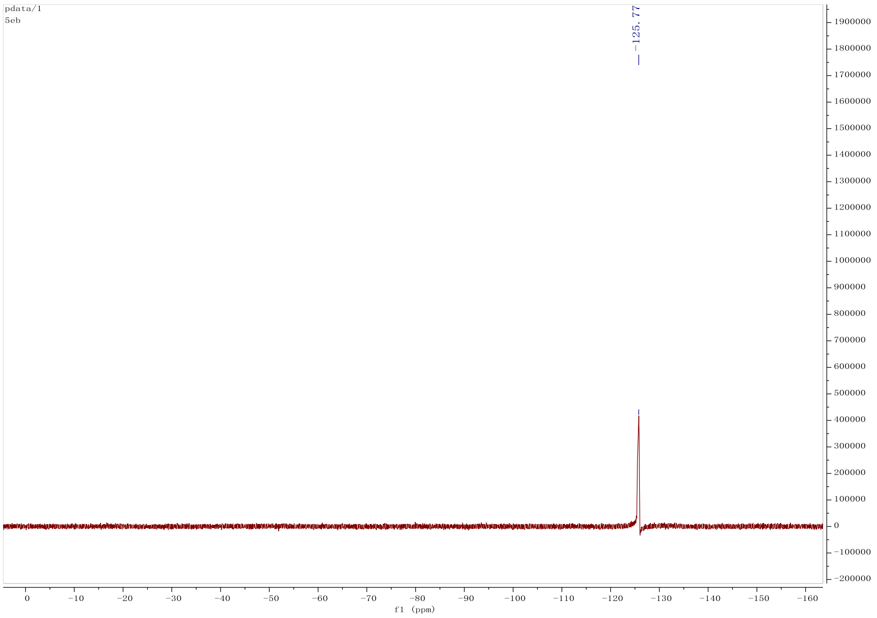

Figure S 67~~Fig. S28.~~ ^{19}F NMR for compound 27 (5eb) 2-(4-chlorophenyl)-6-fluoro-2,3-dihydroquinazolin-4(1H)-one.

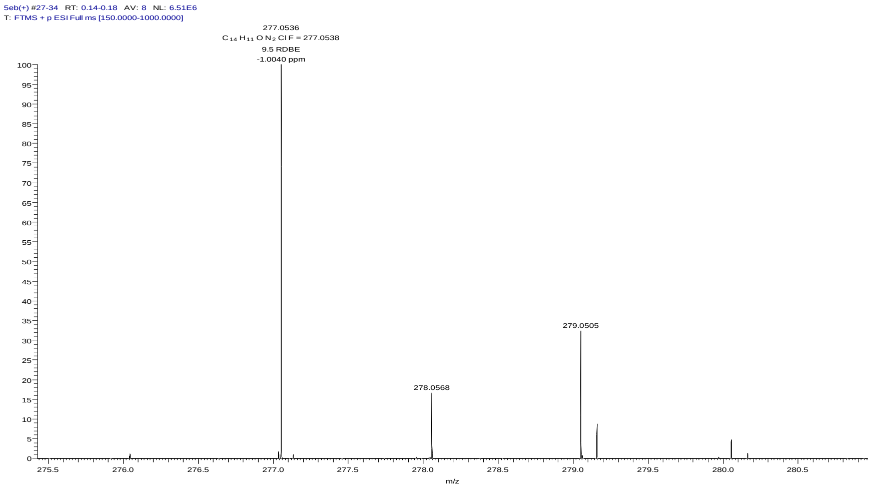

Figure S68HRMS for compound 27 (5eb) 2-(4-chlorophenyl)-6-fluoro-2,3-dihydroquinazolin-4(1H)-one.

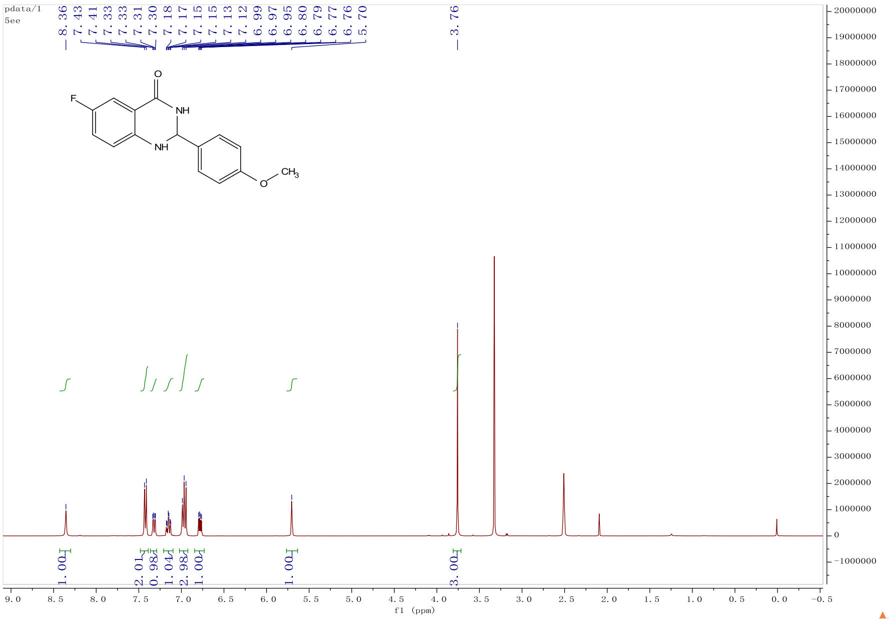

FigureS69

Fig. S29. ^{1}H NMR for compound 28 (5ee)6-fluoro-2-(4-methoxyphenyl)-2,3-dihydroquinazolin-4(1H)-one.

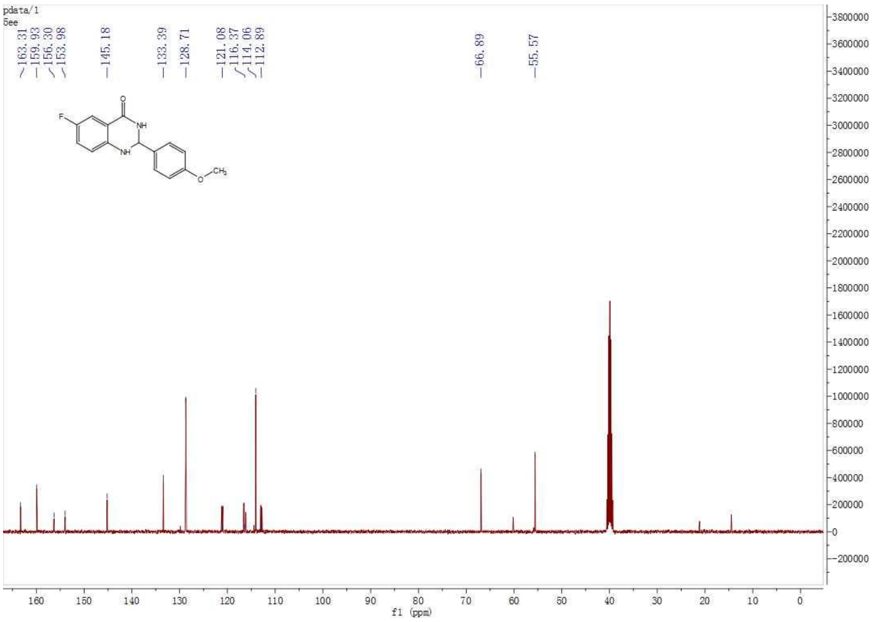

Figure S70Fig. S29. ^{13}C NMR for compound 28 (5ee) 6-fluoro-2-(4-methoxyphenyl)-2,3-dihydroquinazolin-4(1H)-one.

Formatted: Font: (Default) Times New Roman

Formatted: Font: (Default) Times New Roman, Not Bold

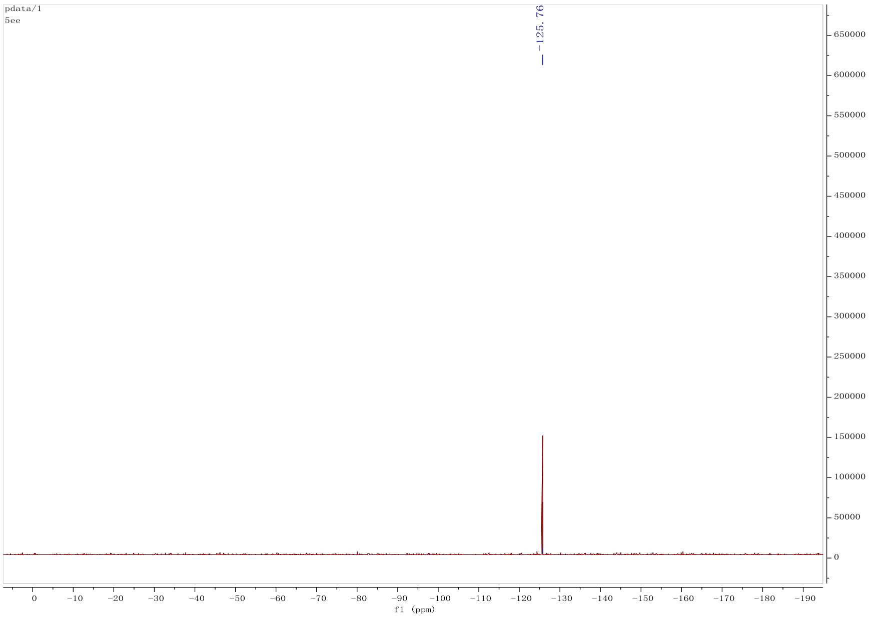

Figure S 71[19]F NMR for compound 28 (5ee) 6-fluoro-2-(4-methoxyphenyl)-2,3-dihydroquinazolin-4(1H)-one.

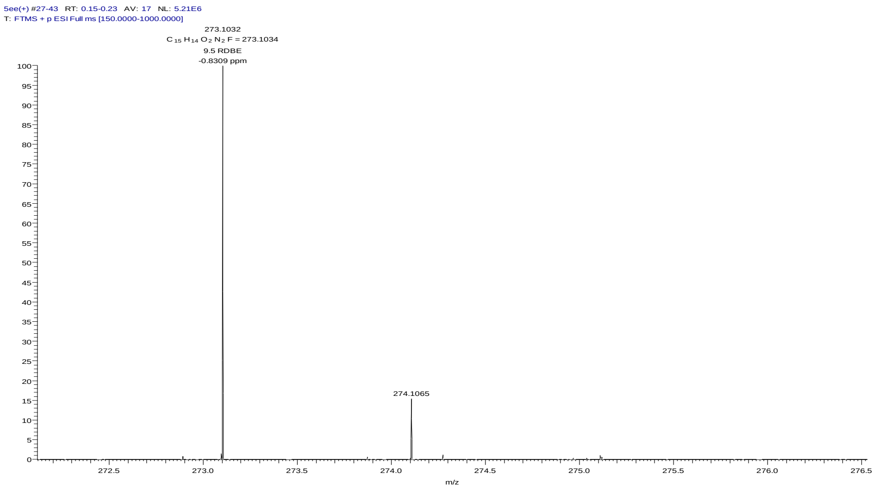

Figure S72HRMS for compound 28 (5ee) 6-fluoro-2-(4-methoxyphenyl)-2,3-dihydroquinazolin-4(1H)-one.

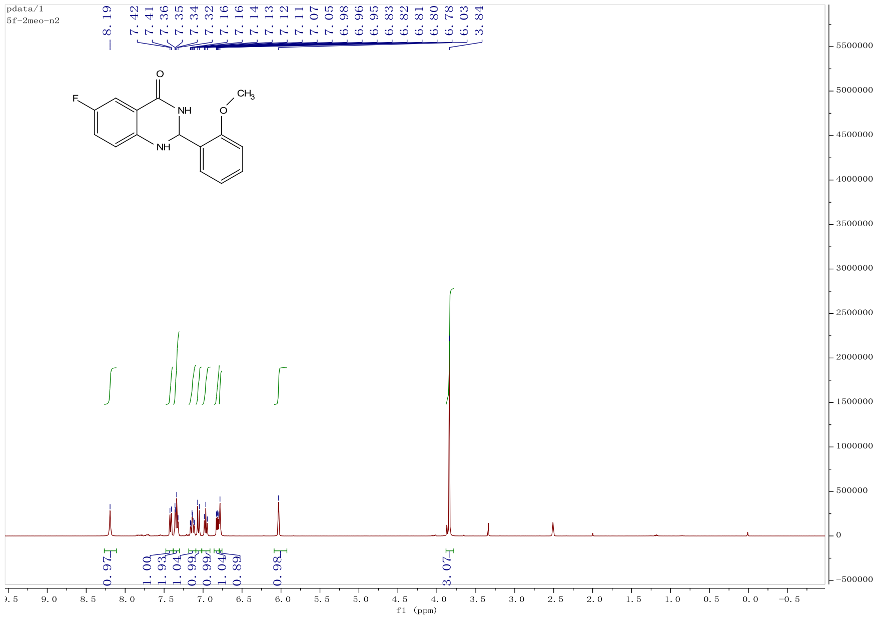

Figure S73

~~Fig. S30.~~ ^{1}H NMR for compound 29 (5eq)6-fluoro-2-(2-methoxyphenyl)-2,3-dihydroquinazolin-4(1H)-one.

Formatted: Font: (Default) Times New Roman

Formatted: Font: (Default) Times New Roman, Not Bold

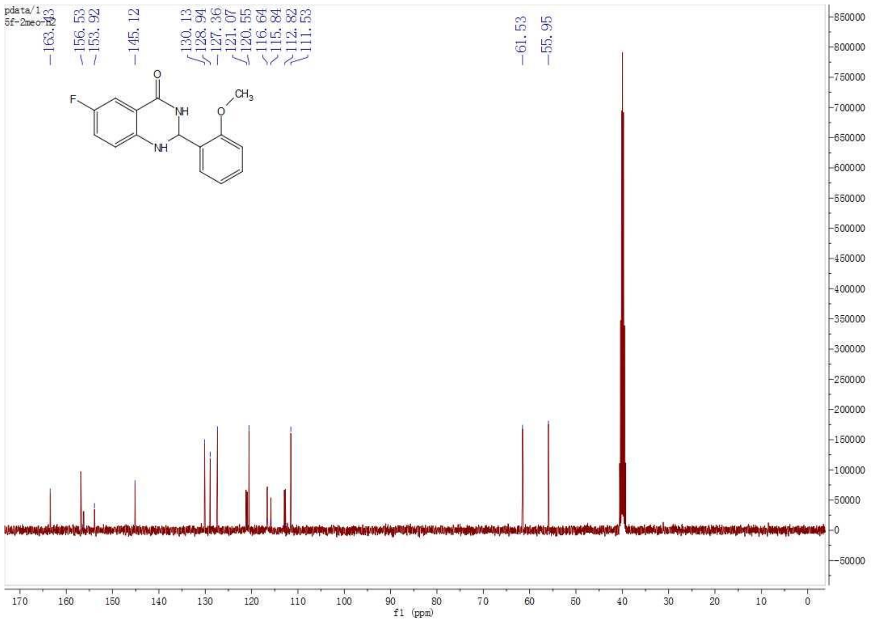

Figure S74~~Fig. S30.~~ ^{13}C NMR for compound 29 (5eq) 6-fluoro-2-(2-methoxyphenyl)-2,3-dihydroquinazolin-4(1H)-one.

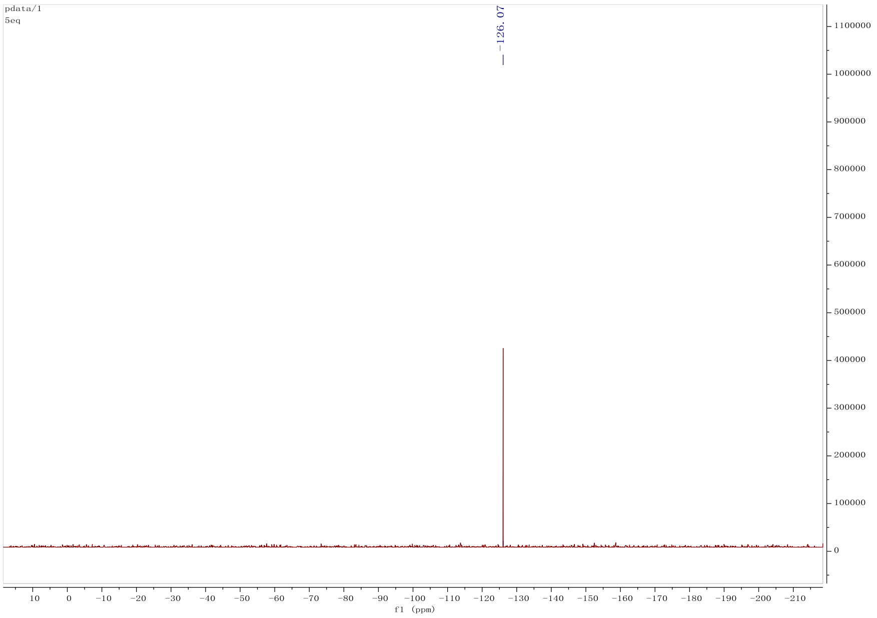

Figure S 75[19]F NMR for compound 29 (5eq) 6-fluoro-2-(2-methoxyphenyl)-2,3-dihydroquinazolin-4(1H)-one.

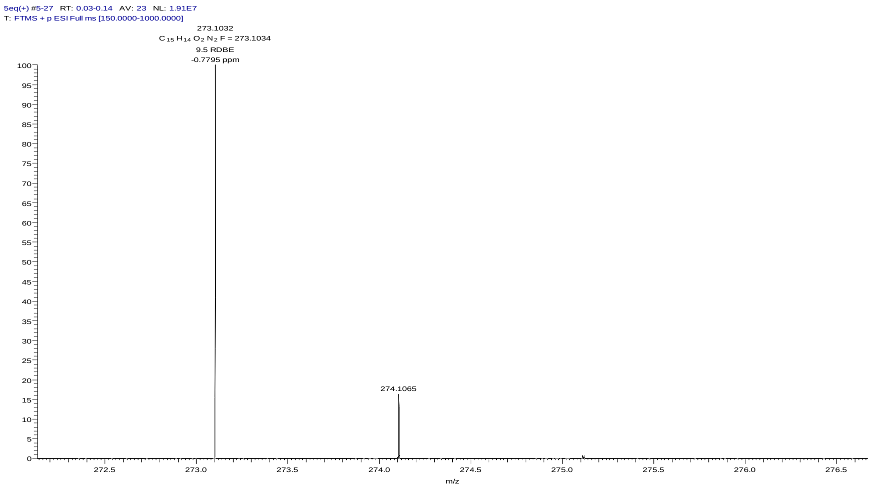

Figure S 76HRMS for compound 29 (5eq) 6-fluoro-2-(2-methoxyphenyl)-2,3-dihydroquinazolin-4(1H)-one.

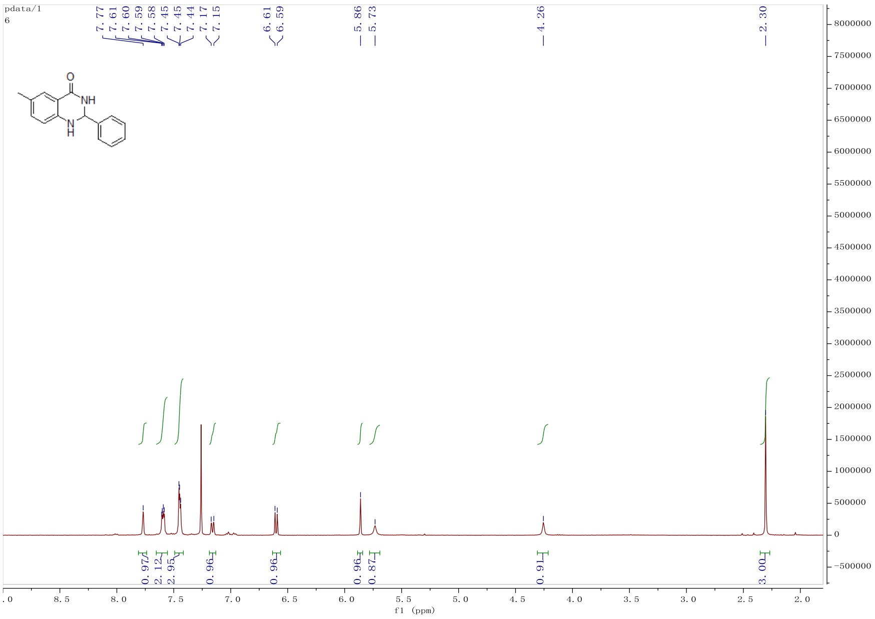

Figure S77

~~Fig. S31.~~ ^{1}H NMR for compound 30 (5fa)6-methyl-2-phenyl-2,3-dihydroquinazolin-4(1H)-one.

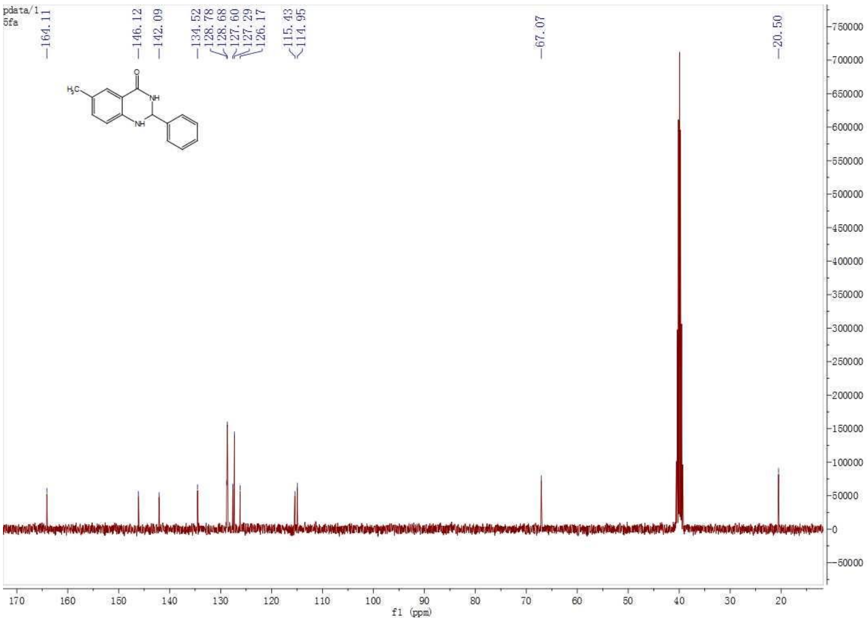

Figure S78 ^{13}C NMR for compound 30 (5fa) 6-methyl-2-phenyl-2,3-dihydroquinazolin-4(1H)-one.

Formatted: Font: (Default) Times New Roman

Formatted: Font: (Default) Times New Roman, Not Bold

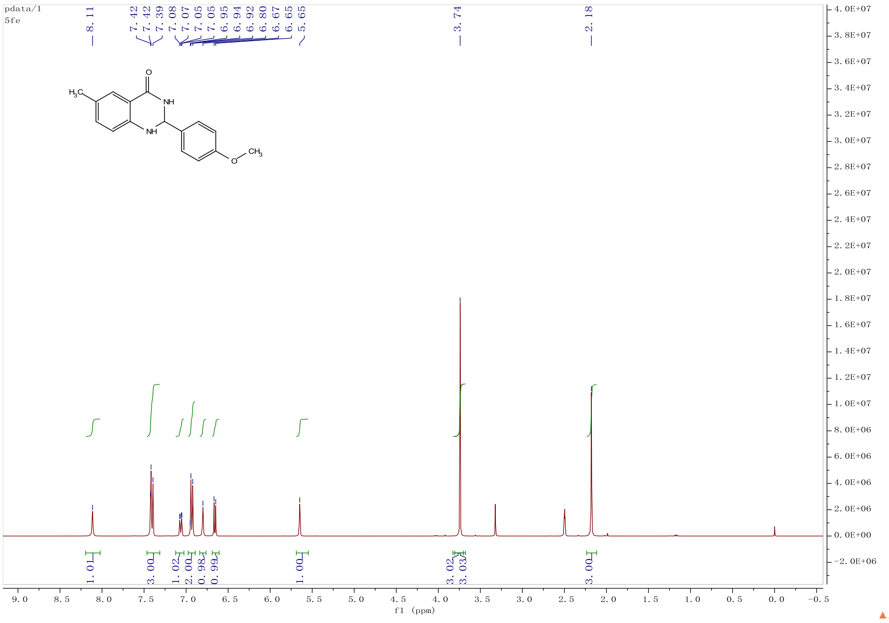

Figure S79

~~Fig. S33.~~ ^{1}H NMR for compound 31 (5fe)2-(4-methoxyphenyl)-6-methyl-2,3-dihydroquinazolin-4(1H)-one.

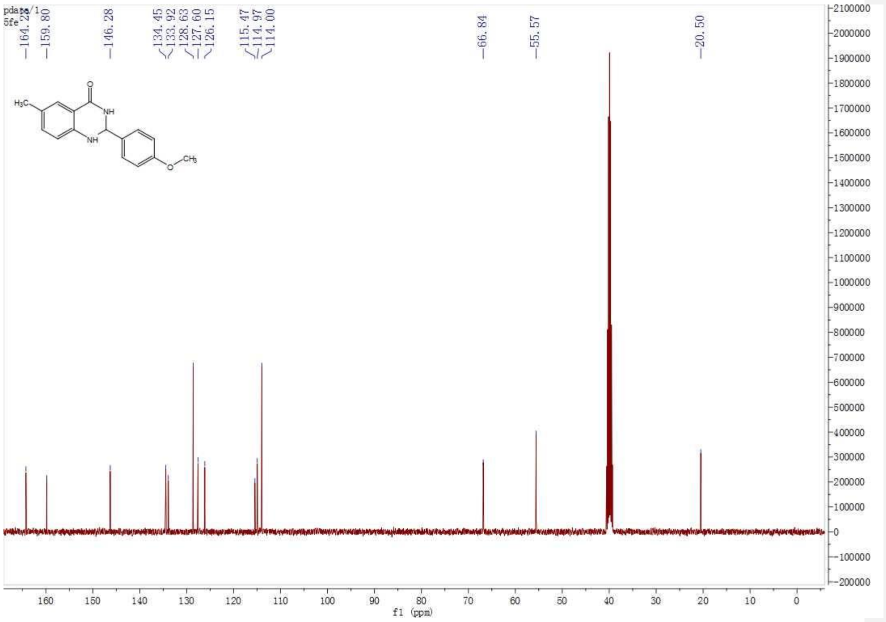

Figure S80 ^{13}C NMR for compound 31 (5fe) 2-(4-methoxyphenyl)-6-methyl-2,3-dihydroquinazolin-4(1H)-one.

Formatted: Font: (Default) Times New Roman

Formatted: Font: (Default) Times New Roman, Not Bold

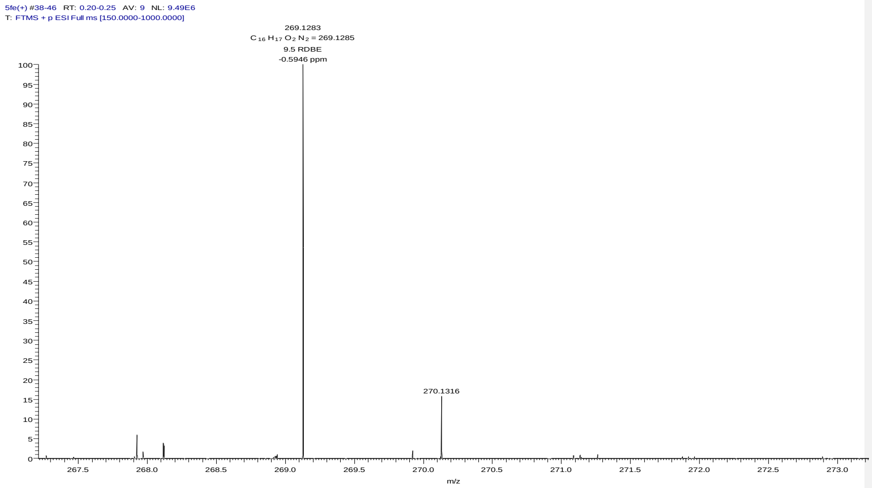

Figure S 81 HRMS for compound 31 (5fe) 2-(4-methoxyphenyl)-6-methyl-2,3-dihydroquinazolin-4(1H)-one.

1. Parua, S.; Das, S.; Sikari, R.; Sinha, S.; Paul, N. D., One-Pot Cascade Synthesis of Quinazolin-4(3H)-ones via Nickel-Catalyzed Dehydrogenative Coupling of *o*-Aminobenzamides with Alcohols. *J Org Chem* **2017,** 82, (14), 7165-7175, 10.1021/acs.joc.7b00643.
2. Mohammed, S.; Vishwakarma, R. A.; Bharate, S. B., Iodine catalyzed oxidative synthesis of quinazolin-4(3H)-ones and pyrazolo[4,3-d]pyrimidin-7(6H)-ones via amination of sp3 C-H bond. *J Org Chem* **2015,** 80, (13), 6915-6921, 10.1021/acs.joc.5b00989.
3. Guo, S.; Li, Y.; Tao, L.; Zhang, W.; Fan, X., Rapid assembly of quinazolinone scaffold via copper-catalyzed tandem reaction of 2-bromobenzamides with aldehydes and aqueous ammonia: application to the synthesis of the alkaloid tryptanthrin. *RSC Adv.* **2014,** 4, (103), 59289-59296, 10.1039/c4ra10799c.
4. Wu, X. F.; He, L.; Neumann, H.; Beller, M., Palladium-catalyzed carbonylative synthesis of quinazolinones from 2-aminobenzamide and aryl bromides. *Chemistry* **2013,** 19, (38), 12635-12638, 10.1002/chem.201302182.
5. Ghosh, S. K.; Nagarajan, R., Deep Eutectic Solvent Mediated Synthesis of Quinazolinones and Dihydroquinazolinones: Synthesis of Natural Products and Drugs. *RSC Adv.***, 2016**,6, 27378-27387, 10.1039/C6RA00855K.
6. Yu, L.; Wang, M.; Li, P.; Wang, L., Fe_3O_4 nanoparticle-supported copper(I): magnetically recoverable and reusable catalyst for the synthesis of quinazolinones and bicyclic pyrimidinones. *Appl Organomet Chem* **2012,** 26, (11), 576-582, 10.1002/aoc.2902.
7. Huang, H.; Liu, S.; Jean, M.; Simpson, S.; Huang, H.; Merkley, M.; Hayashi, T.; Kong, W.; Rodriguez-Sanchez, I.; Zhang, X.; Yosief, H. O.; Miao, H.; Que, J.; Kobie, J. J.; Bradner, J.; Santoso, N. G.; Zhang, W.; Zhu, J., A Novel Bromodomain Inhibitor Reverses HIV-1 Latency through Specific Binding with BRD4 to Promote Tat and P-TEFb Association. *Front Microbiol* **2017,** 8, 1035, 10.3389/fmicb.2017.01035.
8. Cao, S. L.; Guo, Y. W.; Wang, X. B.; Zhang, M.; Feng, Y. P.; Jiang, Y. Y.; Wang, Y.; Gao, Q.; Ren, J., Synthesis and Cytotoxicity Screening of Piperazine-1-carbodithioate Derivatives of 2-Substituted Quinazolin-4(3*H*)-ones. *Archiv Der Pharmazie* **2010,** 342, (3), 182-189, 10.1002/ardp.200800148.
9. Hayakawa, M.; Kaizawa, H.; Moritomo, H.; Koizumi, T.; Ohishi, T.; Okada, M.; Ohta, M.; Tsukamoto, S.; Parker, P.; Workman, P., Synthesis and biological evaluation of 4-morpholino-2-phenylquinazolines and related derivatives as novel PI3 kinase p110alpha inhibitors. *Bioorg Med Chem* **2006,** 14, (20), 6847-6858, 10.1016/j.bmc.2006.06.046.
10. Wu, X.-F.; Oschatz, S.; Block, A.; Spannenberg, A.; Langer, P., Base mediated synthesis of 2-aryl-2,3-dihydroquinazolin-4(1H)-ones from 2-aminobenzonitriles and aromatic aldehydes in water. *Org Biomol Chem* **2014,** 12, (12), 1865-1870, 10.1039/C3OB42434K.
11. Shiri, L.; GhorbaniâChoghamarani, A.; Kazemi, M., Cu(II) immobilized on Fe_3O_4â“diethylenetriamine: A new magnetically recoverable catalyst for the synthesis of 2,3â��dihydroquinazolinâ��4(1*H*)â��ones and oxidative coupling of thiols. *Appl Organomet Chem* **2017,** 31, (5), e3596, 10.1002/aoc.3596.

~~12~~
